# Inhibition of pyrimidine biosynthesis targets protein translation in acute myeloid leukemia

**DOI:** 10.15252/emmm.202115203

**Published:** 2022-05-06

**Authors:** Joan So, Alexander C Lewis, Lorey K Smith, Kym Stanley, Rheana Franich, David Yoannidis, Lizzy Pijpers, Pilar Dominguez, Simon J Hogg, Stephin J Vervoort, Fiona C Brown, Ricky W Johnstone, Gabrielle McDonald, Danielle B Ulanet, Josh Murtie, Emily Gruber, Lev M Kats

**Affiliations:** ^1^ The Peter MacCallum Cancer Centre Melbourne Vic. Australia; ^2^ The Sir Peter MacCallum Department of Oncology University of Melbourne Parkville Vic. Australia; ^3^ Human Oncology and Pathogenesis Program Memorial Sloan Kettering Cancer Center New York NY USA; ^4^ Australian Centre for Blood Diseases Monash University Melbourne Vic. Australia; ^5^ Servier Pharmaceuticals Boston MA USA

**Keywords:** acute myeloid leukemia, DHODH, leukemic stem cells, protein translation, Cancer, Haematology, Metabolism

## Abstract

The mitochondrial enzyme dihydroorotate dehydrogenase (DHODH) catalyzes one of the rate‐limiting steps in *de novo* pyrimidine biosynthesis, a pathway that provides essential metabolic precursors for nucleic acids, glycoproteins, and phospholipids. DHODH inhibitors (DHODHi) are clinically used for autoimmune diseases and are emerging as a novel class of anticancer agents, especially in acute myeloid leukemia (AML) where pyrimidine starvation was recently shown to reverse the characteristic differentiation block in AML cells. Herein, we show that DHODH blockade rapidly shuts down protein translation in leukemic stem cells (LSCs) and has potent and selective activity against multiple AML subtypes. Moreover, we find that ablation of CDK5, a gene that is recurrently deleted in AML and related disorders, increases the sensitivity of AML cells to DHODHi. Our studies provide important molecular insights and identify a potential biomarker for an emerging strategy to target AML.

## Graphical abstract

This study reports that AG636, an inhibitor of the metabolic enzyme DHODH, has excellent potency against acute myeloid leukemia (AML) in pre‐clinical models.

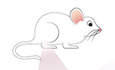

**The paper explained**

**Problem**

AML is a low survival cancer with a 5‐year overall survival rate of < 30%. Selective metabolic dependencies in leukemic cells offer significant promise for the development of new therapeutic strategies. We explored the molecular and cellular effects of targeting *de novo* pyrimidine synthesis using an inhibitor of the metabolic enzyme DHODH.

**Results**

We found that the DHODH inhibitor AG636 rapidly downregulates the protein translation pathway in LSCs. We identify YY1 and its associated INO80 chromatin remodeling complex as a key sensor that mediates this molecular response. We also demonstrate that loss of CDK5, a gene that is recurrently deleted in a subset of AML patients that have a poor prognosis and can be targeted by existing drugs, sensitizes cells to DHODHi treatment.

**Impact**

Our study adds to the growing body of evidence that DHODH inhibitors have potent and selective activity against AML cells *in vivo*. Our data provide novel insights into the molecular mechanism of action of DHODH inhibition and identify a potential biomarker for identifying patients that are most likely to respond to treatment.

## Introduction

AML is an aggressive malignancy with few effective treatment options and extremely poor outcomes in the majority of cases. The 5‐year overall survival rate is less than 30%, and for a large proportion of patients who have unfavorable prognostic factors, the median survival is < 1 year (Papaemmanuil *et al*, 2016). Hence, there is an urgent unmet need to develop novel therapeutic strategies, particularly those that engage different mechanisms of action compared with drugs in current clinical use (Nair *et al*, 2021).

Pyrimidine bases are components of many biological macromolecules including DNA and RNA and are essential for cell growth. Although mammalian cells can acquire pyrimidines from salvage pathways, most cells rely predominantly on *de novo* synthesis to meet their metabolic requirements. DHODH is a flavoprotein that is localized on the inner mitochondrial membrane and catalyzes the fourth step of *de novo* pyrimidine biosynthesis, the ubiquinone‐mediated oxidation of dihydroorotate to orotate. As an enzyme that is associated with the electron transport chain, DHODH links nucleotide synthesis with mitochondrial bioenergetics and ROS production (Vyas & Ghate, 2011; Sykes, 2018; Wang *et al*, 2021).

Inhibition of DHODH has been proposed as a therapeutic strategy in a range of human diseases from viral infection, to autoimmunity and cancer (Vyas & Ghate, 2011; Sykes, 2018; Wang *et al*, 2021). The FDA‐approved DHODHi leflunomide has been clinically utilized as an immune‐suppressant for the treatment of rheumatoid arthritis and multiple sclerosis, with its efficacy proposed to stem from selective antiproliferative effects on high‐affinity T‐cells (Klotz *et al*, 2019). Leflunomide and another DHODHi, brequinar, have been trialed in various cancers, and although occasional durable responses were observed, the initial data were insufficient to support further clinical development (Vyas & Ghate, 2011; Sykes, 2018; Wang *et al*, 2021). Notably, however, recent findings suggesting the selective efficacy of DHODHi in hematological malignancies have prompted renewed interest in DHODH as an anticancer target and have spurred extensive efforts to develop more potent and selective next‐generation inhibitors (Sykes *et al*, 2016; Cao *et al*, 2019; Christian *et al*, 2019; Zhou *et al*, 2020). Intriguingly, AML cells have been reported to undergo myeloid maturation in response to pyrimidine starvation (Sykes *et al*, 2016; Cao *et al*, 2019; Christian *et al*, 2019; Zhou *et al*, 2020), suggesting a link between nutrient availability and cell fate that can be exploited as a form of “differentiation therapy.”

To facilitate further development and clinical deployment of DHODHi, there remains a need for a comprehensive analysis of molecular and cellular responses, especially in rare leukemia stem cells (LSCs) that underpin disease progression and therapy resistance (Pollyea & Jordan, 2017). Herein, we used AG636, a novel potent DHODHi that has been extensively validated by biophysical studies and metabolic profiling (McDonald *et al*, 2020), to characterize the link between nucleotide metabolism and therapeutic efficacy. Our findings demonstrate that DHODH inhibition has antiproliferative and prodifferentiation activity *in vivo* and potent activity against multiple AML subtypes. By integrating data across a panel of syngeneic mouse models and human AML cells lines, we characterize molecular pathways that are directly triggered by DHODHi and identify a novel predictive biomarker and combination strategy.

## Results

### DHODH inhibition has potent antileukemic activity in MLL‐rearranged AML *in vivo*

**Figure EV1:**
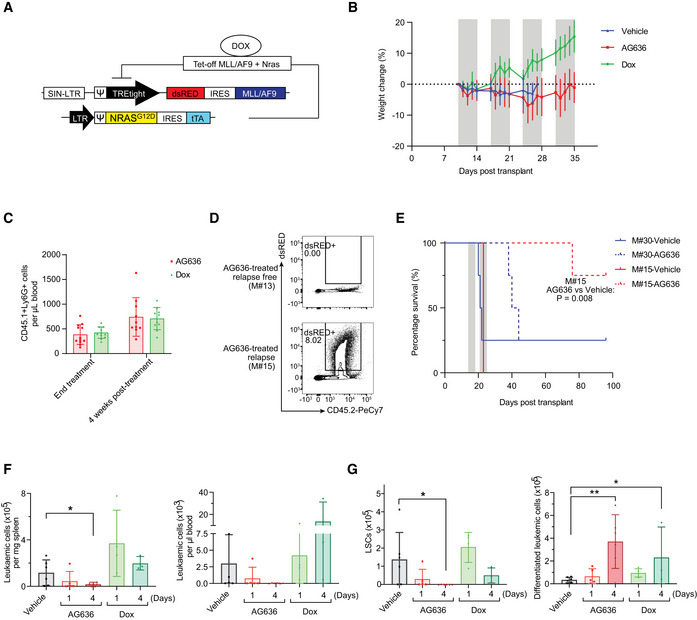
Efficacy of DHODH inhibition in the MN murine AML model Schematic of MN model.Body weight of MN tumor‐bearing mice treated with AG636. Gray bars denote treatment. Dotted line defines zero percent weight loss.Number of recipient‐derived myeloid cells (CD45.1^+^CD11b^+^Ly6G^+^) in the peripheral blood of AG636‐ or doxycycline‐treated recipients at the conclusion of therapy and after 4 weeks (*n = *8–10 mice/group).Representative FACS plots of the bone marrow from a mouse with no detectable disease (M#13) and a relapsed mouse (M#15).Kaplan–Meier survival curve of secondary recipients transplanted with leukemic cells from the relapsed donor (M#15) or a control donor from the vehicle group (M#30). Gray bars denote treatment (*n* = 4 mice/group, median survival is 21.5 for vehicle‐treated M#30, 42 for AG636‐treated M#30, 23 for vehicle‐treated M#15, and not reached for AG636‐treated M#15, the *P* value was calculated by log‐rank test).Number of MN cells in the spleen and peripheral blood quantified by flow cytometry (*n = *3–6 mice/group).Number of LSCs (CD11b^low^cKit^high^FcgR^+^) and differentiated leukemic cells (CD182^+^Ly6G^+^) in the bone marrow (*n = *3–6 mice/group). Data information: data in F‐G are presented as mean ± SD; *P* values were calculated using a one‐tailed Student’s unpaired *t*‐test. *P < 0.05, **P < 0.01, Dox—doxycycline. Source data are available online for this figure.

Inhibition of DHODH has been reported to induce differentiation of AML blasts *in vitro* and *in vivo*, but the pathways that underpin this phenotypic response remain unknown (Sykes *et al*, 2016; Cao *et al*, 2019; Christian *et al*, 2019; Zhou *et al*, 2020). To gain insights into the mode of action of DHODH‐targeting drugs we focused initially on an aggressive chemo‐refractory syngeneic murine AML model driven by doxycycline‐inducible expression of MLL‐AF9 and constitutive expression of oncogenic Nras^G12D^ (hereafter referred to as MN) (Fig EV1A) (Zuber *et al*, 2009, 2011). We and others have previously shown that leukemic cells are addicted to continued expression of the MLL‐AF9 oncoprotein and that silencing it drives terminal myeloid differentiation (Zuber *et al*, 2011; Ghisi *et al*, 2016). We reasoned that comparing MLL‐AF9 depletion with AG636 treatment may aid in distinguishing primary cellular and molecular changes triggered directly by DHODH inhibition from secondary changes that occur as a consequence of differentiation.

**Figure 1:**
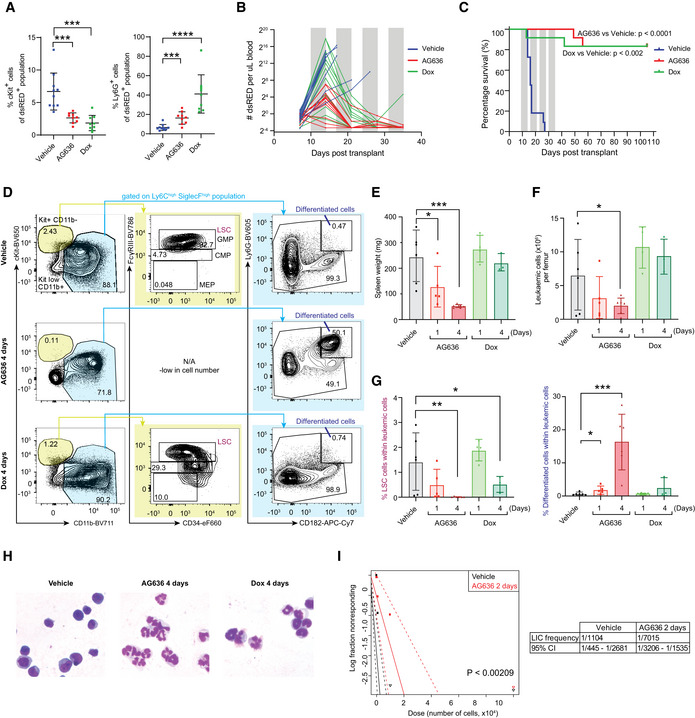
AG636 is an effective single‐agent therapy in MLL‐rearranged AML Frequency of MN leukemic cells expressing the immature marker cKit and mature myeloid marker Ly6G in the peripheral blood following 5 days of treatment (*n = *9–10 mice/group; mice with < 2% tumor burden in any condition were censored from the analysis).Absolute number of MN cells in the peripheral blood quantified by flow cytometry. Gray bars denote treatment (*n = *11–12 mice/group).Kaplan–Meier survival curve of leukemic mice (*n* = 11–12 mice/group, median survival is 16.5 for vehicle and not reached for AG636 and doxycycline, *P* < 0.0001 by log‐rank test).Representative FACS plots showing differentiation induced by AG636 and doxycycline.Spleen weights of MN tumor‐bearing mice (*n = *3–6 mice/group).Absolute number of MN cells in the bone marrow (*n = *3–6 mice/group).Frequency (as percentage of all MN cells) of LSCs (CD11b^low^cKit^high^FcgR^+^) and differentiated cells (CD182^+^Ly6G^+^) in the bone marrow (*n = *3–6 mice/group).May‐Grunwald‐Giemsa‐stained cytospins of sorted MN cells showing myeloid differentiation.Quantification of functionally defined LSCs using a limiting dilution assay calculated by ELDA (*n* = 4–8 mice/group). Data information: data in A and E‐G are presented as mean ± SD; *P* values were calculated using a one‐tailed Student’s unpaired *t*‐test. *P < 0.05, **P < 0.01, ***P < 0.001, ****P < 0.0001; Dox—doxycycline. Source data are available online for this figure.

We first performed a longitudinal study monitoring disease progression and differentiation status of leukemic cells at weekly intervals by flow cytometric analysis of peripheral blood. We administered AG636 at a dose of 100 mg/kg body weight b.i.d. on days 1–5 of a 7‐day cycle, a regimen that was well‐tolerated (Fig EV1B). AG636 marginally reduced disease progression after one cycle of treatment, and we observed evidence of differentiation as indicated by reduced expression of cKit and increased expression of Ly6G (Fig 1A and B). Excitingly, following cycle two, we observed disease regression in the AG636 group, and four cycles of treatment were sufficient to induce clinical complete remission with no detectable MN cells in the peripheral blood of 10 out of 12 mice (Fig 1B and C).

After four cycles, treatment was withdrawn and we continued monitoring surviving animals. To assess the impact of AG636 on normal myelopoiesis recovery, we quantified recipient‐derived myeloid cells (Fig EV1C). We found no differences between AG636 and doxycycline‐treated mice either at the conclusion of therapy or after 4 weeks. These data suggest that AG636 did not significantly exacerbate myelosuppression over and above that caused by 3.5 Gy whole‐body irradiation (used for conditioning prior to tumor engraftment) and tumor development itself. The majority of animals remained tumor free for more than 8 weeks and extensive FACS analysis of the bone marrow at the endpoint failed to detect any minimal residual disease in 9 survivors. Two mice did relapse, however, (M#15 and M#16) and one other animal required euthanasia, although no tumor cells were detected in its blood or bone marrow (Fig EV1D). To determine whether relapsed MN cells were resistant to DHODH inhibition, we transplanted these cells into a cohort of secondary recipients. We also transplanted a separate control cohort with MN cells from a vehicle‐treated animal (M#30) that had never been exposed to AG636. In both cohorts, drug treatment (using a reduced 2‐week regimen) was equally effective, demonstrating that even though leukemic cells could occasionally persist during 4 weeks of therapy, they retained a high degree of AG636 sensitivity at relapse (Fig EV1E). The observation that 2 weeks of treatment in the secondary transplant for the M30 leukemia was less effective than 4 weeks of treatment in the primary transplant suggests that the extended treatment duration may provide additional benefit.

To characterize the cellular response of MN cells that underpins the therapeutic efficacy of DHODH inhibition, we next performed additional short‐term treatments. Mice bearing MN tumors were treated with doxycycline or AG636 for 1 or 4 days and detailed immunophenotyping was performed on the peripheral blood, spleen, and bone marrow compartments (Figs 1D–G and EV1F and G). As expected, doxycycline rapidly silenced MLL‐AF9 expression and induced myeloid differentiation of MN cells, evidenced by a reduction in the frequency and absolute number of phenotypically defined LSCs (CD11b^low^cKit^high^FcγR3^+^)(Krivtsov *et al*, 2006) and a concomitant increase in differentiated granulocyte‐like cells (CD182^+^Ly6G^+^) (Figs 1G and EV1G). DHODH inhibition similarly induced myeloid differentiation, but notably, the phenotype was different from that induced by genetic depletion of MLL‐AF9. In particular, AG636 rapidly reduced splenomegaly and the total number of leukemic cells in the peripheral blood, spleen, and bone marrow of drug‐treated animals (Figs 1E–G and EV1F and G). Consistent with the flow cytometric data, morphological analysis of sorted MN cells revealed the presence of myeloid maturation in both doxycycline‐ and AG636‐treated mice (Fig 1H). Importantly, the viable MN cells remaining after 2 days of AG636 therapy possessed reduced leukemia‐initiating capacity in limiting dilution re‐transplant experiments, demonstrating functional loss of LSCs (Fig 1I). Overall, AG636 has excellent potency against MLL‐rearranged AML, effectively targets LSCs, and induces rapid disease regression through a combination of cell death and differentiation.

### Inhibition of *de novo* pyrimidine synthesis is broadly effective against multiple AML subtypes

**Figure 2:**
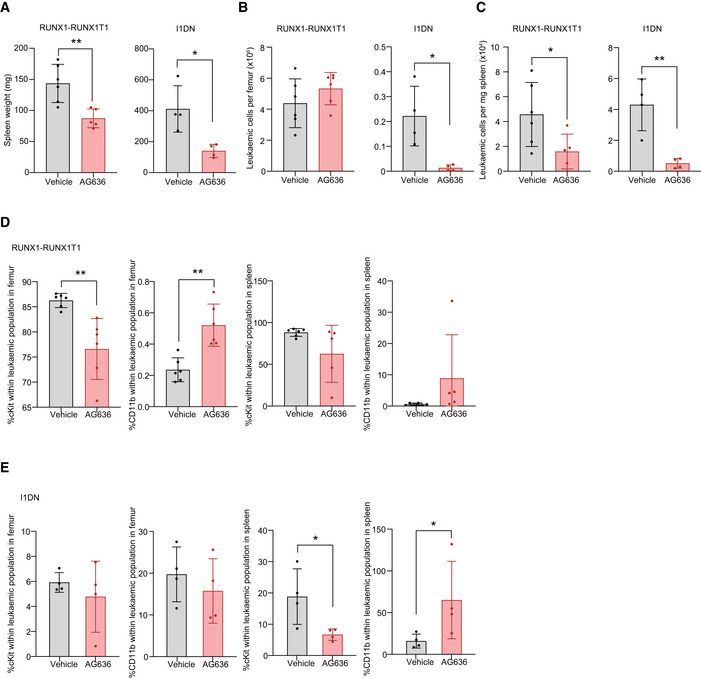
DHODH inhibition induces differentiation and inhibits proliferation in multiple AML subtypes ASpleen weights of RUNX1‐RUNX1T1 or I1DN tumor‐bearing mice treated with AG636 or vehicle for 4 days.B, CAbsolute number of leukemic cells in the bone marrow (B) and spleens (C) of RUNX1‐RUNX1T1 or I1DN tumor‐bearing mice treated with AG636 or vehicle for 4 days.D, EFrequency of leukemic cells expressing the immature marker cKit and mature myeloid marker CD11b in the bone marrow and spleens of RUNX1‐RUNX1T1 (D) or I1DN (E) tumor‐bearing mice treated with AG636 or vehicle for 4 days (*n = *6 mice/group). Data information: *n = *5–6 mice/group for RUNX1‐RUNX1T1 model, *n* = 4 mice/group for I1DN model; data are presented as mean ± SD; *P* values were calculated using a one‐tailed Student’s unpaired *t*‐test. *P < 0.05, **P < 0.01. Source data are available online for this figure.

To determine whether AG636 has efficacy in other subtypes of AML we tested two additional murine models that carry distinct combinations of AML driver genes using an endpoint of tumor burden following 4 days of treatment. Recipient mice were engrafted with established tumors expressing the core‐binding factor fusion protein RUNX1‐RUNX1T1 (also known as AML1‐ETO) (Bots *et al*, 2014) or the combination of mutant alleles of *IDH1^R132H^*, *DNMT3A^R882H^*, and *Nras^G12D^* (hereafter referred to as I1DN, see Appendix Fig S1A and Methods for details) and randomized to receive vehicle or AG636 therapy. Both models co‐expressed fluorescent reporters, enabling precise compartment‐specific quantification of leukemic burden. In both contexts, we observed reduced tumor progression in the drug group (Fig 2A–C). In the RUNX1‐RUNX1T1 model, the spleen weights of AG636‐treated mice were significantly reduced, and although the overall number of leukemic cells in the bone marrow were comparable with controls, fewer cells expressed cKit and more cells expressed CD11b demonstrating differentiation (Fig 2A–D and Appendix Fig S1B). In the I1DN model, AG636 reduced the spleen weight and the total number of leukemic cells in both the spleen and bone marrow (Fig 2A–C). Unlike in the MN and RUNX1‐RUNX1T1 AMLs, there was minimal impact on the expression of cKit and CD11b in the bone marrow, suggesting that the predominant effect of AG636 in this context was to inhibit proliferation and/or trigger cell death (Fig 2E and Appendix Fig S1D). Interestingly, in both RUNX1‐RUNX1T1 and I1DN models, we observed increased surface expression of Sca1, a marker associated not only with stemness but also with Type I interferon (IFN) signaling (Essers *et al*, 2009; Appendix Fig S1C and E). Taken together, our data demonstrate that the therapeutic potential of DHODH inhibition in AML is not limited to individual disease subsets.

### Effects of DHODH inhibition on normal hematopoiesis

**Figure 3:**
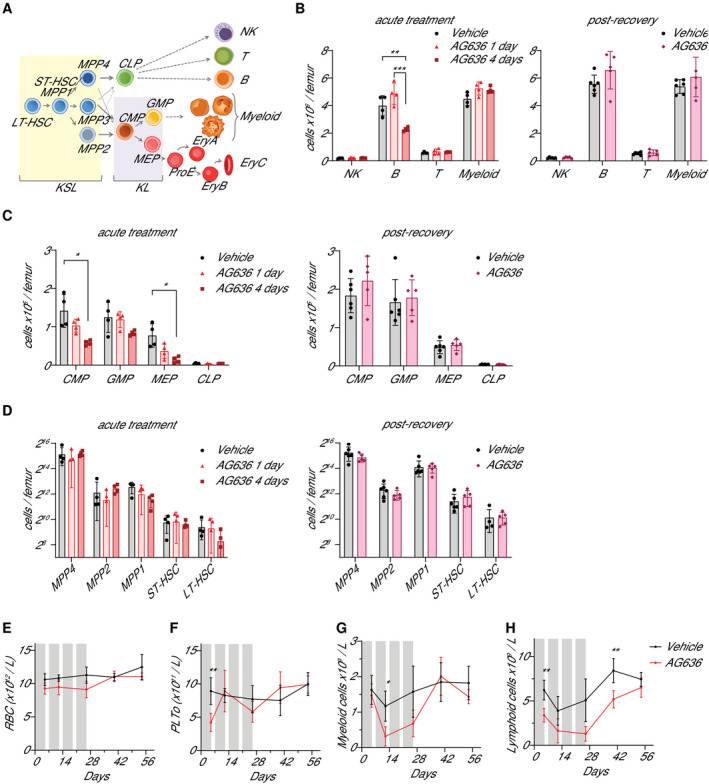
DHODH inhibition has a minor impact on normal blood development ASchematic of hematopoietic differentiation.B–DVarious bone marrow populations were quantified by flow cytometry in mice treated with AG636 or vehicle for 1 or 4 days (acute treatment) or for 4 cycles followed by 4 weeks off treatment (post‐recovery).E–HPeripheral blood red blood cells (E), platelets (F), myeloid cells (G), and lymphoid cells (H) were quantified in mice treated with AG636 or vehicle. Gray bars denote treatment. Data information: *n = *4 mice/group for acute treatment, *n = *5–6 mice for prolonged treatment and recovery; data are presented as mean ± SD; *P* values were calculated using a two‐tailed Student’s unpaired *t*‐test; only comparisons that meet the threshold of *P* < 0.05 are shown. *P < 0.05, **P < 0.01, ***P < 0.001; see Table EV1 for abbreviations and markers. Source data are available online for this figure.

The clinical utility of potential anticancer agents that target proteins that are expressed in nonmalignant cells is dependent on the existence of a therapeutic window. As DHODH is widely expressed in healthy hematopoietic cells, we sought to assess the impact of AG636 on normal blood development *in vivo*. We treated non‐tumor‐bearing mice with AG636 for 1 or 4 days, the same regimen that had a significant impact on leukemia development, and quantified stem, progenitor, and mature cell populations from all three major hematopoietic lineages (lymphoid, myeloid, erythro‐megakaryocytic) (Fig 3A and Table EV1). After 4 days of drug treatment, there was a significant loss of B cells in the bone marrow and minor‐moderate reductions in common myeloid progenitors, megakaryocyte‐erythroid progenitors, and long‐term hematopoietic stem cells (Fig 3B–D and Appendix Fig S2). In the erythroid compartment the immature ProE, EryA, and EryB fractions were reduced by AG636 whereas the EryC fraction was increased suggesting a burst of erythroid differentiation (Appendix Fig S3A). Spleen size, mature myeloid, T and NK cells, multipotent progenitors, and short‐term hematopoietic stem cells were unaffected by 4 days of AG636 treatment (Fig 3B–D and Appendix Fig S3B). It should be noted that quantification of stem and progenitor populations is potentially confounded by the upregulation of Sca1 expression as noted above.

Although the overall consequences of acute DHODH inhibition on normal hematopoietic cells were minor in comparison with the effects observed on AML cells, they did indicate the potential for longer treatment regimens to cause therapy‐related toxicity including myelosuppression. We next treated mice for four cycles with AG636 and tracked the effects in peripheral blood over time. AG636 did not alter red blood cell counts but resulted in a small reduction in platelets and moderate reductions in circulating lymphoid and myeloid cells while mice remained on treatment (Fig 3E–H and Appendix Fig S3C). Notably, however, after treatment was withdrawn, all numbers progressively returned to baseline levels (Fig 3E–H and Appendix Fig S3C). Analysis of bone marrow populations after a 4‐week recovery period showed no differences between animals that had received AG636 and vehicle‐treated controls (Fig 3B–D). Collectively, these data are consistent with the observation that as many as four weekly cycles of AG636 treatment are well‐tolerated even in sublethally irradiated tumor‐bearing mice and point to a selective vulnerability of AML cells to pyrimidine starvation.

### AG636 downregulates protein synthesis pathways in LSCs

LSCs play a central role in AML pathogenesis, and their metabolic requirements and gene expression programs are distinct from those of “bulk” leukemic cells (Pollyea & Jordan, 2017; Yamashita *et al*, 2020). To gain insights into the molecular pathways that are perturbed by DHODHi in LSCs, we performed RNA sequencing (RNAseq). We initially focused on the MN model and compared the effects of AG636 with differentiation induced by genetic depletion of MLL‐AF9. To that end, we sorted and analyzed cKit^high^CD11b^low^ immature MN cells that are highly enriched in functional LSCs (Krivtsov *et al*, 2006) from mice treated with vehicle, AG636 for 1 day, or doxycycline for 1 or 4 days. We were unable to perform analysis following 4 days of AG636 treatment due to insufficient numbers of cKit^high^CD11b^low^ MN cells.

**Figure 4:**
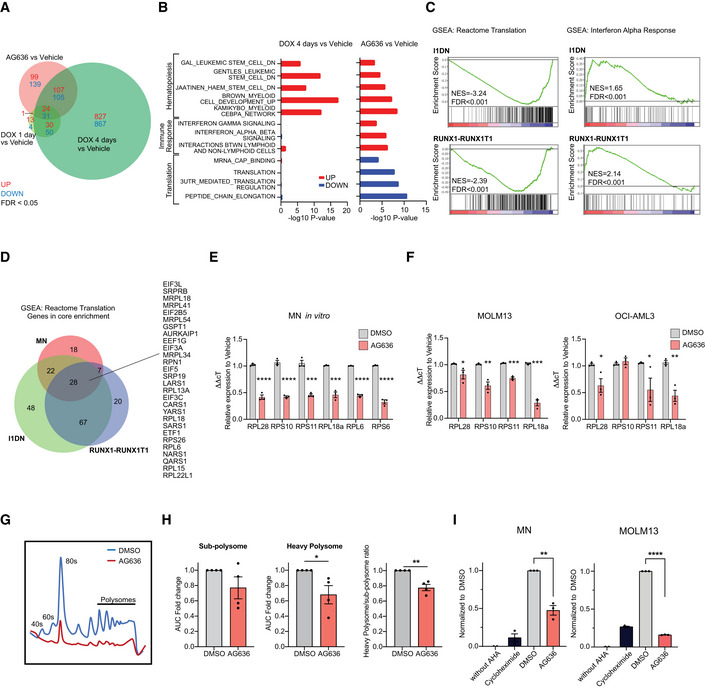
AG636 induces transcriptional downregulation of genes required for protein translation A, BRNA sequencing performed on cKit^high^CD11b^low^ MN cells sorted from AG636, doxycycline, or vehicle‐treated mice (*n = *3 mice/group). Venn diagram showing overlap in DEGs between different treatment conditions (A). Gene set enrichment analysis showing common and differential enrichment of biological pathways in gene expression data from AG636‐ or doxycycline‐treated animals. Gene sets are from C2:CGP and Reactome subcollections in MSigDB database (see methods for more information) (B).CRNA sequencing performed on cKit^+^CD11b^‐^ RUNX1‐RUNX1T1 or I1DN cells sorted from mice treated with AG636 or vehicle for 1 day (*n = *3 mice/group). Bar code plots showing enrichment of selected pathways.DVenn diagram showing the overlap of genes in the core enrichment within the Reactome Translation gene set in the MN, RUNX1‐RUNX1T1, and I1DN models.E, FqPCR showing downregulation of genes encoding ribosomal proteins in MN cells (E) and human AML cell lines (F) treated for 24 h with AG636 (*n = *3 biological replicates for each cell line).G, HPolysome profiling of MN cells treated *in vitro* with AG636 or vehicle for 24 h. Representative trace (G) and quantification of subpolysome and heavy polysome fractions (H) determined by measuring the area under the curve (AUC) (*n = *4 biological replicates).INascent protein synthesis quantified using the AHA incorporation assay in MN or MOLM13 cells treated with AG636 for 24 h or cycloheximide for 1 h. Cells that were cultured in the absence of AHA served as a negative control (*n = *3 biological replicates). Data information: data in E, F, H, and I are presented as mean ± SEM; *P* values were calculated using a one‐tailed Student’s unpaired *t*‐test; *P < 0.05, **P < 0.01, ***P < 0.001, ****P < 0.0001. Source data are available online for this figure.

Using stringent statistical cut‐offs, we identified differentially expressed genes (DEGs) in all treatment conditions (Dataset EV1). As expected, doxycycline induced robust transcriptional changes, particularly at the 4‐day time point. Consistent with previous studies, we observed perturbation of classic MLL‐AF9‐regulated genes including *Myb*, *Hoxa5*, *Hoxa9*, *Tcf4*, *and Id2* (Zuber *et al*, 2011; Ghisi *et al*, 2016). Altered gene expression induced by AG636 partially overlapped with changes induced by MLL‐AF9 depletion, although 47% of DEGs (99/231 genes upregulated by AG636 and 139/275 genes downregulated by AG636) were unique to DHODH inhibitor treatment (Fig 4A). We performed gene set enrichment analysis (Subramanian *et al*, 2005) to identify pathways that may be common or unique between the treatment conditions. Confirming our phenotypic observations, gene signatures associated with myeloid differentiation were highly positively enriched in both treatment conditions. In contrast, gene sets related to protein translation (downregulated) and type I interferon signaling (upregulated) were uniquely enriched in AG636‐treated cells (Fig 4B). Of note, inhibition of *de novo* pyrimidine synthesis has previously been reported to trigger the expression of interferon‐stimulated genes (Lucas‐Hourani *et al*, 2013), and type I interferons have been implicated in the differentiation of LSCs (Hemmati *et al*, 2017).

**Figure EV2:**
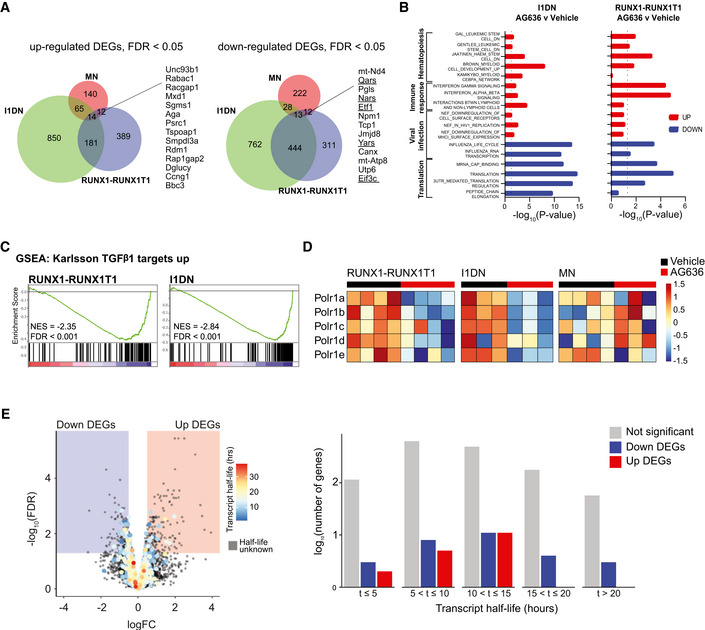
Comparison of transcriptional effects of DHODHi in different AML models Venn diagram showing the overlap in DEGs in the MN, RUNX1‐RUNX1T1, and I1DN models.Gene set enrichment analysis showing the enrichment of selected biological pathways in gene expression data from RUNX1‐RUNX1T1 and I1DN murine AML models following AG636 treatment. Gene sets are from C2:CGP and Reactome subcollections in MSigDB database (see methods for more information).Barcode plots showing downregulation of TGF‐β signaling in RUNX1‐RUNX1T1 and I1DN murine AML models following AG636 treatment.Gene expression heat map showing downregulation of genes encoding components of RNA polymerase I in RUNX1‐RUNX1T1 and I1DN murine AML models following AG636 treatment.Volcano plot of gene expression in MN cells, highlighting the average transcript half‐life of each gene (left) and bar chart of the number of genes with transcript half‐lives in the given interval for significant DEGs (right). Transcript half‐life was provided by (Schwanhäusser *et al*, 2011).

Recent studies have demonstrated that LSCs from different patients and model systems share many common properties despite their unique collection of driver mutations (Lagadinou *et al*, 2013; Jones *et al*, 2018, 2020; Pollyea *et al*, 2018). To determine whether the transcriptional effects of AG636 were conserved in non‐MN LSCs, we analyzed LSC‐enriched populations from the RUNX1‐RUNX1T1 and I1DN models (cKit^high^ and cKit^+^CD11b^‐^ cells, respectively). We found that DHODHi perturbed similar pathways in all three contexts, although there was some variation in the particular DEGs within those pathways that reached statistical thresholds (Fig 4C and EV2A and B, Datasets EV2 and EV3). We also detected some model‐specific changes, including transforming growth factor β (TGF‐β) signaling and RNA polymerase I components that were downregulated in RUNX1‐RUNX1T1 and I1DN LSCs, but not MN LSCs (Fig EV2C and D). Overall, the magnitude of change was greatest for transcripts encoding ribosomal proteins and translation initiation factors such as *Rpl15*, *Eif5*, and *Eif3A* (Fig 4D). Interestingly, mRNAs that encode these proteins have been shown to have a relatively long half‐life in mammalian cells (Schwanhäusser *et al*, 2011; Forrest *et al*, 2020). In contrast, transcripts with a short half‐life such as *Myc* were not affected by AG636 treatment. Moreover, unbiased analysis using a dataset of mRNA stability from murine NIH3T3 fibroblasts (Schwanhäusser *et al*, 2011) revealed no consistent trends in perturbation of short‐lived transcripts (Fig EV4E). Consistent with our *in vivo* data in LSCs, we found that ribosomal protein genes were downregulated in MN cells and human AML cell lines treated *in vitro* with AG636 (Fig 4E and F). Importantly these changes occurred within 24 h of drug administration and preceded the loss of viability of the cells (Appendix Fig S4A).

**Figure EV3:**
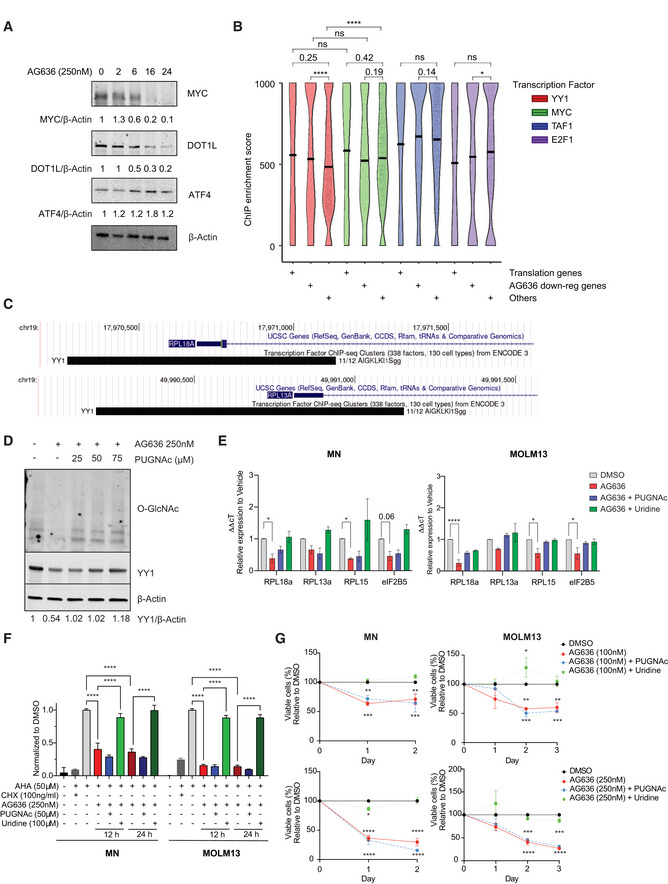
YY1 is a downstream target of AG636 in AML Western blot of ATF4, DOT1L, and MYC in MN cells treated with AG636 for 24 h.Violin plot of YY1 ChIPseq enrichment scores at the promoter regions (+1,000 bp to −50 bp from TSS) of genes within the indicated gene sets extracted from ENCODE 3 (*n* = 24 genes for translation genes, *n* = 707 genes for AG636 downregulated genes, *n* = 4,237 for the other genes).Screenshot from UCSC genome browser (http://genome.ucsc.edu/index.html) of YY1 enrichment at the promoter regions of translation genes RPL13A and PRL18A in 11 out of 12 cell lines in ENCODE3 data.Western blot for YY1 in MN cells co‐treated with AG636 and PUGNAc for 24 h.qPCR showing the expression of translation genes in MOLM13 (right) or MN cells (left) co‐treated with AG636 and PUGNAc or uridine for 24 h.Nascent protein synthesis quantified using the AHA incorporation assay in MN or MOLM13 cells co‐treated with AG636 and PUGNAc or uridine for 24 h (*n = *3 biological replicates).Proliferation assay in MN or MOLM13 cells co‐treated with AG636 and PUGNAc or uridine for 24 h (*n = *3 biological replicates). Data information: data in E, F, and G are presented as mean ± SEM; *P* values were calculated using a two‐tailed Student’s unpaired *t*‐test in B, a one‐tailed Student’s unpaired *t*‐test in E, a one‐way ANOVA with the Tukey’s test for multiple comparisons in F, and a 2‐way ANOVA with the Šídák's test for multiple comparisons in G; *P < 0.05, **P < 0.01, ***P < 0.001, ****P < 0.0001. Source data are available online for this figure.

To determine whether the transcriptional perturbations affected the assembly of ribosomal subunits we performed polysome profiling. As these experiments require a large amount of material, we treated MN cells *in vitro* with AG636. As expected, DHODH inhibition caused a profound reduction in heavy polysomes and the heavy polysome to subpolysome ratio (Fig 4G and H). Finally, we measured protein synthesis by feeding cells with L‐Azidohomoalanine (AHA), an amino acid analog of methionine that is incorporated into nascent proteins and can be detected using click chemistry and FACS. AG636 significantly dampened AHA incorporation in both MN and MOLM13 cells prior to the cells dying or initiating apoptosis as measured by Annexin V staining (Fig 4I and Appendix Fig S4B). Altogether, these findings suggest that downregulation of protein translation pathways is a specific and early response of AML cells to DHODH inhibition that occurs at the level of mRNA synthesis and is not attributable to global dampening of transcription caused by reduced availability of pyrimidine nucleotides.

### Analysis of chromatin accessibility identifies YY1 as a key transcription factor that modulates gene expression downstream of DHODH inhibition

**Figure 5:**
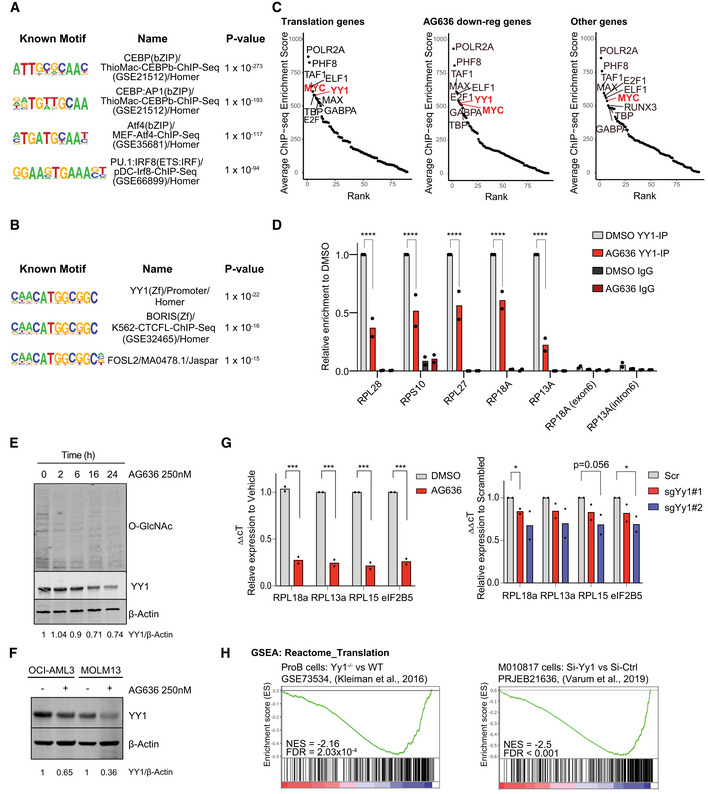
Downregulation of YY1 protein contributes to altered gene expression in AML cells following DHODH inhibition A, BATAC sequencing performed on cKit^high^CD11b^low^ MN cells sorted from mice treated with AG636 or vehicle for 2 days (*n = *3 mice/group). HOMER motif analysis showing transcription factor motifs enriched within regions of differential chromatin accessibility (A). HOMER motif analysis showing the enrichment of YY1 motifs within regions of accessible chromatin associated with translation genes. All other regions of open chromatin were used as the background (B).CRank plots of ChIPseq enrichment scores of transcription factors at the promoter regions (+1,000 bp to −50 bp from TSS) of genes within the indicated gene sets extracted from ENCODE 3 (*n* = 24 genes for translation genes, *n* = 707 genes for AG636 downregulated genes, *n* = 4,237 for the other genes).DChIPqPCR showing the binding of YY1 at the promoter regions of selected translation genes in MN cells treated with AG636 or DMSO. For each replicate, enrichment was normalized to YY1 pull‐down in DMSO‐treated cells (*n = *2 biological replicates).ETime‐course showing global downregulation of O‐GlcNAcylation and YY1 expression in MN cells treated with AG636. The experiment was repeated twice with similar results.FWestern blot of YY1 expression in human AML cell lines treated with AG636 for 24 h.GqPCR showing downregulation of genes encoding ribosomal proteins that are putative YY1 targets in Cas9‐expressing MN cells treated with AG636 or DMSO (left), or transduced with sgRNAs targeting YY1 or control sgRNAs (right) (*n = *2 biological replicates).HBarcode plots showing downregulation of the Reactome Translation gene set in YY1 knockout pro‐B cells and human melanoma cells upon YY1 knockdown (Kleiman *et al*, 2016; Varum *et al*, 2019). Data information: data in D and G are presented as mean ± SEM; *P* values were calculated using a two‐tailed Student’s unpaired *t*‐test in D and a one‐tailed Student’s unpaired *t*‐test in G; *P < 0.05, ***P < 0.001, ****P < 0.0001. Source data are available online for this figure.

To identify transcription factors (TFs) that drive altered gene expression in AML cells in response to pyrimidine starvation *in vivo* we mapped open chromatin using the assay for transposase‐accessible chromatin (ATACseq). We concentrated our analysis on the MN model and specifically on the cKit^high^CD11b^low^ population that is highly enriched in LSCs. We identified approximately 150,000 peaks that were distributed at or near transcription start sites (TSS), within gene bodies or intergenic regions (Appendix Fig S5A and B). As expected, TSS‐associated open regions were strongly correlated with gene expression (Appendix Fig S5C). AG636‐induced selective alterations to chromatin accessibility, with approximately 2% of all ATAC peaks showing differential signals between drug and vehicle‐treated cells (Appendix Fig S5A). Most of the differential peaks were present in intergenic regions, suggesting that AG636 treatment alters enhancer utilization (Appendix Fig S5D). Supporting our morphological and gene expression findings, regions of increased accessibility induced by DHODH inhibition were enriched in DNA motifs for known myeloid differentiation factors including PU.1 and members of the CEBP family (Fig 5A). These regions were also enriched for ATF4 motifs, suggesting that drug treatment activated the integrated stress response in LSCs as previously reported in colorectal cancer cells following mitochondrial complex III inhibition (Evstafieva *et al*, 2014). Concordantly, ATF4 protein was increased in MN cells treated with AG636 *in vitro* (Fig EV3A).

**Figure EV4:**
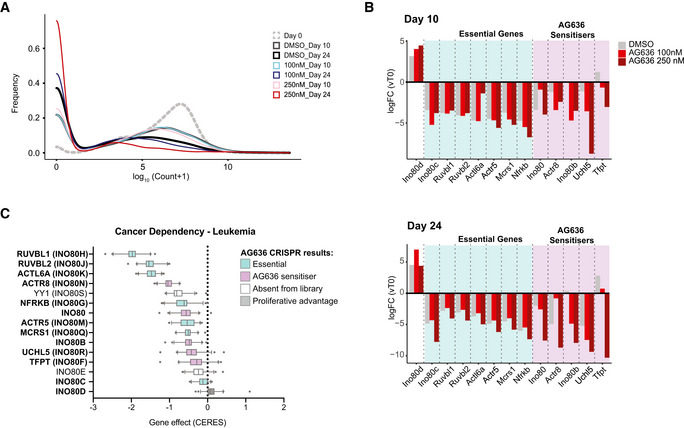
Identification of genes that increase or decrease the sensitivity of AML cells to DHODHi Distribution of sgRNA counts in the various conditions during the CRISPR screen.Average fold change in sgRNA counts at time point 10 (top) and at time point 24 (bottom) compared with time point 0 for all sgRNAs targeting components of the INO80 complex in the various conditions in the CRISPR screen.Gene dependencies in AML cell lines from the DepMap database (Meyers *et al*, 2017), center line; median; box limits, from the 25^th^ to 75^th^ percentiles; whiskers, from the 5^th^ to 95^th^ percentiles (*n* = 47 cell lines). Source data are available online for this figure.

We next focused our attention on genes involved in protein translation (hereafter “translation genes”), as the coordinated downregulation of this pathway represents the most striking and conserved phenotype triggered by AG636 that we observed in AML LSCs *in vivo*. We used the Pscan algorithm to identify over‐represented TF‐binding sites within the proximal promoters of translation genes (Zambelli *et al*, 2009). This analysis identified members of the YY and ETS families as the most highly enriched (Dataset EV4). YY motifs were also highly enriched in translation gene‐associated regions of open chromatin compared with other regions of open chromatin in MN LSCs (Fig 5B). YY1, but not YY2 was highly expressed in all three murine AML models, and in publicly available human AML gene expression data (Appendix Fig S5E) (Cancer Genome Atlas Research Network *et al*, 2013). Notably, YY1 has previously been implicated as a transcriptional activator of the translation pathway (Zurkirchen *et al*, 2019). As an orthogonal approach, we explored the ENCODE database, which contains > 1,200 ChIP sequencing (ChIPseq) datasets for ~340 chromatin‐associated factors (ENCODE Project Consortium, 2012). In an unbiased analysis (see Materials and Methods for details), YY1 was among the most enriched TFs localized at the promoters of translation genes, along with MYC (Figs 5C and EV3B and C). Notably, the enrichment of YY1 at translation genes, and a broader set of AG636 downregulated DEGs, was significantly greater than its enrichment at expressed genes not perturbed by AG636. To provide direct evidence that YY1 is present at the promoters of translation genes in MN cells we performed ChIP qPCR for a subset of putative YY1 targets. As expected, YY1 was enriched at the promoters of Rpl28, Rps10, Rpl27, Rpl18a, and Rpl13a and depleted upon drug treatment (Fig 5D).

YY1 activity is known to be regulated by O‐Linked N‐Acetylglucosaminylation (O‐GlcNAc), a common protein post‐translational modification that requires pyrimidine synthesis (Hiromura *et al*, 2003; Hart *et al*, 2007; Sykes *et al*, 2016). Confirming previous reports (Sykes *et al*, 2016; Christian *et al*, 2019), we found that DHODH inhibition rapidly reduced global O‐GlcNAc protein modification in MN cells (Fig 5E). Loss of O‐GlcNAc coincided with downregulation of YY1 protein (Fig 5E). AG636 treatment similarly caused downregulation of YY1 protein, but not a transcript, in human AML cell lines MOLM13 and OCI‐AML3 (Fig 5F and Appendix Fig S5F). Notably, the phenotype was more pronounced in MOLM13 cells, which are more sensitive to AG636‐induced killing compared with OCI‐AML3 cells where the effects of the inhibitor at early time points are predominantly cytostatic (Appendix Fig S4A). We also analyzed the expression of MYC and DOT1L, two other proteins with essential functions in AML that are stabilized by O‐GlcNAc (Sykes *et al*, 2016; Song *et al*, 2021). MYC and DOT1L were similarly downregulated by AG636, likely contributing to the antiproliferative effects of the drug (Fig EV3A).

We next used CRISPR to disrupt *YY1* in MN cells using two independent sgRNAs. YY1 is a common essential gene as defined by the Cancer Dependency Map (DepMap) (Meyers *et al*, 2017), and MN cells containing *YY1*‐targeting sgRNAs and co‐expressing a Crimson (Crim) fluorescent reporter were depleted over time in culture (Appendix Fig S5G). To determine the impact of YY1 deletion on the expression of translation genes, we sorted viable newly transduced cells on day 3 post‐viral infection. At this time point, *YY1* sgRNA‐expressing cells had reduced levels of YY1 protein and showed partial downregulation of translation genes that contained a YY1 motif within their promoter (Fig 5G and Appendix Fig S5H). To provide further evidence that YY1 is a transcriptional regulator of translation genes we analyzed two publicly available datasets in murine pro‐B cells and human melanoma cells generated using a conditional *YY1* allele and *YY1* siRNA, respectively (Kleiman *et al*, 2016; Varum *et al*, 2019). In both contexts, inactivation or silencing of YY1 led to the downregulation of translation genes (Fig 5H).

O‐GlcNAc modification is regulated by the reciprocal activity of two evolutionary conserved enzymes—O‐GlcNAc transferase (OGT), which deposits the mark, and O‐GlcNAcase (OGA), which removes it (Hart *et al*, 2007). We tested whether inhibition of OGA could rescue AG636‐mediated phenotypes. To that end, we co‐treated MN cells with AG636 and PUGNAc, an inhibitor of OGA. Concordant with our hypothesis, PUGNAc countered the loss of YY1 protein caused by AG636 and also partially reversed the transcriptional downregulation of translation genes in MOLM13 cells (Fig EV3D and E). However, PUGNAc could not restore nascent protein synthesis or the antiproliferative effects of AG636. In contrast uridine, a key metabolite downstream of DHODH that has been shown to compensate for *de novo* pyrimidine synthesis in hematological cell lines (Sykes *et al*, 2016; Cao *et al*, 2019; McDonald *et al*, 2020), abrogated the effects of AG636 on both cell growth and translation (Fig EV3F and G). Thus, our data suggest that the downregulation of YY1 protein downstream of DHODH inhibition, combined with additional factors, drives the downregulation of the protein translation pathway following drug treatment.

### Loss of CDK5 and INO80 complex sensitizes AML cells to inhibition of *de novo* pyrimidine synthesis

**Figure 6:**
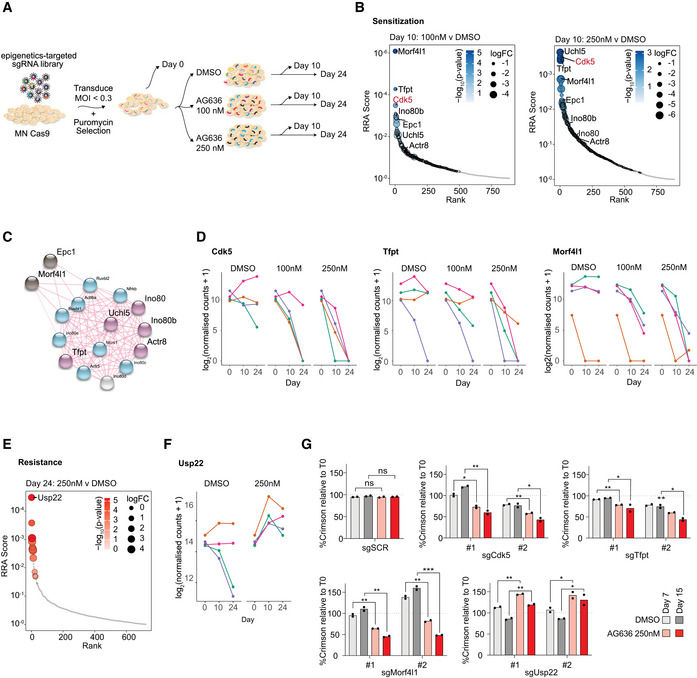
Pooled CRISPR screen identifies CDK5 and INO80 complex as modulators of AG636 sensitivity Schematic of the pooled CRISPR screen in MN AML cells.Rank plot of MAGeCK analysis showing genes that were negatively selected at day 10 in cells treated with 100 or 250 nM AG636.STRING network analysis showing known interactions between components of the INO80 complex and associated proteins. Blue—INO80 complex genes that are essential (negatively enriched in all conditions); light gray—INO80 complex gene that was positively enriched in all conditions; purple—INO80 complex genes that are AG636 sensitizers (negatively enriched in AG636 condition only); black—other AG636 sensitizers identified in the screen.Normalized counts for sgRNAs targeting Cdk5, Tfpt, and Morf4l1.Rank plot of MAGeCK analysis showing genes that were positively selected at day 24 in cells treated with 250 nM AG636.Normalized counts for sgRNAs targeting Usp22.Proliferative competition assays in MN cells transduced with individual sgRNAs and cultured in AG636 or DMSO (*n = *2 biological replicates). Data information: data in G represented as mean ± SEM; *P* values were calculated using one‐tailed Student’s unpaired *t*‐test; *P < 0.05, **P < 0.01, ***P < 0.001. Source data are available online for this figure.

Pooled CRISPR screens are a powerful approach to systematically determine key factors that mediate drug resistance and sensitivity. To complement our analyses of the molecular and cellular responses of AML cells to DHODHi, we performed a CRISPR knockout screen to uncover genes that increase or decrease treatment efficacy. As epigenetic regulators are frequently dysregulated in AML (Cancer Genome Atlas Research Network *et al*, 2013) and many are amenable to therapeutic targeting with existing molecules (Dawson, 2017), we focused specifically on this class of genes. MN cells were transduced with an epigenetics‐targeted sgRNA library, cultured in DMSO or increasing concentrations of AG636 and the relative distribution of sgRNAs at the beginning of the experiment, and after 10 and 24 days of culture quantified by sequencing (Fig 6A). We ranked positively and negatively selected sgRNAs and genes using the MAGeCK algorithm (Li *et al*, 2014) and integrated data across different conditions and time points.

To identify genes that were negatively selected in the presence of the drug, we concentrated on day 10 at which time the relative abundance of most sgRNAs compared with day 0 was unaffected (Fig EV4A). One of the most prominent genes in this analysis was Cdk5 with all 4 sgRNAs in the library being lost in a drug concentration and time‐dependent manner (Fig 6B and D). Additionally, reinforcing our findings implicating YY1 as a major effector of the transcriptional response to DHODH inhibition, many of the other top‐ranked sensitizer hits were components of the INO80 chromatin remodeling complex. This included core proteins Tfpt, Actr8, Ino80b, and Uchl5, and associated proteins Morfl1 and Epc1 (Figs 6B–D and EV4B). INO80 is physically associated with YY1 and is recruited to YY1 target loci to activate gene expression (Cai *et al*, 2007). The INO80 factors that did not synergize with AG636 treatment were essential for the proliferation of MN cells and analysis of DepMap data (Meyers *et al*, 2017) confirmed that these genes were broadly essential in AML (Fig EV4B and C).

Conversely, positive selection was most obvious on day 24 in the high AG636 concentration when many sgRNAs were depleted (Fig EV4A). The histone deubiquitinase Usp22 was the most significantly positively enriched gene in our library with the representation of all 4 guides being significantly increased in drug‐treated cells compared with both day 0 and DMSO conditions (Fig 6E and F).

We further validated the phenotype of a subset of genes identified in the screen using two sgRNAs per gene in individual competition assays. As expected, Usp22 targeting sgRNAs mediated resistance to AG636, whereas Cdk5, Tfpt, and Morfl1 guides synergized with drug treatment (Fig 6G). Thus, we have uncovered novel factors that have not previously been implicated in regulating the sensitivity of AML cells to DHODH inhibition.

### Inhibition of CDK5 and DHODH has synergistic activity in AML

**Figure 7:**
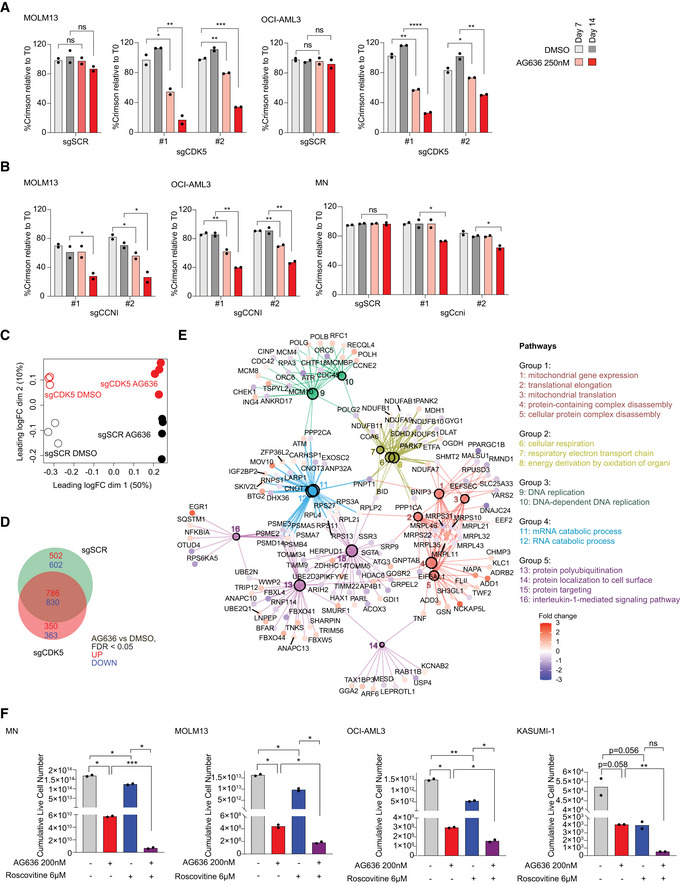
Genetic or pharmacological inhibition of CDK5 increases the sensitivity of AML cells to blockade of pyrimidine synthesis A, BProliferative competition assays in AML cells transduced with indicated sgRNAs and cultured in AG636 or DMSO (*n = *2 biological replicates).C–ERNA sequencing performed on MOLM13 cells transduced with CDK5 or control sgRNAs and treated with AG636 or DMSO (*n = *3–4 biological replicates). Multidimensional scaling (MDS) analysis showing the separation of samples by genotype and drug treatment (C). Venn diagram showing the overlap in DEGs between different treatment conditions (D). Network analysis showing enriched GO terms within CDK5 KO‐specific DEGs (see Materials and Methods for more information) (E).FAML cells were treated with AG636, Roscovitine, or the combination for 17 days. Cumulative live cell counts are shown (*n = *2 biological replicates). Data information: data represented as mean ± SEM; *P* values were calculated using a one‐tailed Student’s unpaired *t*‐test in A and B and the Brown–Forsythe and Welch ANOVA test in F; *P < 0.05, **P < 0.01, ***P < 0.001, ****P < 0.0001. Source data are available online for this figure.

**Figure EV5:**
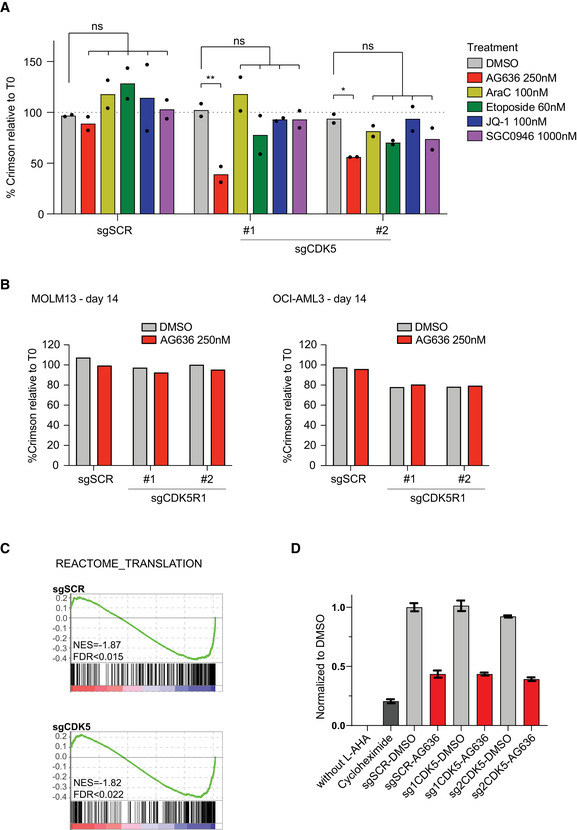
CDK5/CCNI expression affects the response to DHODHi in AML Proliferative competition assays in human AML cell lines transduced with CDK5‐targeting or scrambled sgRNAs and cultured in various inhibitors or DMSO (*n = *2 biological replicates). Dashed line defines no changes compared with time point 0.Proliferative competition assays in human AML cell lines transduced with CDK5R1 sgRNAs and cultured in AG636 or DMSO.Barcode plots showing downregulation of the Reactome Translation gene set in MOLM13 cells transduced with CDK5‐targeting or scrambled sgRNAs and treated with AG636 or DMSO for 24 h.Nascent protein synthesis quantified using the AHA incorporation assay in MOLM13 cells transduced with CDK5‐targeting or scrambled sgRNAs and treated with AG636 or DMSO for 24 h. MOLM13 cells treated with cycloheximide for 1 h or cultured in the absence of AHA served as controls (*n* = 3 biological replicates). Data information: data in A and D are presented as mean ± SD; *P* values were calculated using a one‐tailed Student’s unpaired *t*‐test in B; *P < 0.05, **P < 0.01. Source data are available online for this figure.

One of the top sensitization hits that were revealed on our screen was CDK5. In the human genome, CDK5 is localized on the long arm of chromosome 7 that is recurrently deleted in AML and myelodysplastic syndrome (MDS) and is associated with poor prognosis (Honda *et al*, 2015). Numerous clinical‐grade molecules that inhibit CDK5 have also been developed. These two factors provided a strong impetus to further explore the interaction between CDK5 loss and DHODH inhibition. Consistent with our observations in MN cells, CDK5‐targeting guides also synergized with AG636 treatment in human AML cell lines MOLM13 and OCI‐AML3 (Fig 7A). Notably, CDK5 was dispensable for cellular proliferation and did not mediate sensitization to cytarabine, etoposide, the DOT1L inhibitor SGC0946, or the BRD4 inhibitor JQ1, demonstrating the selective nature of the phenotype (Figs 7A and EV5A; Appendix Fig S6A).

CDK5 is an atypical member of the cyclin‐dependent kinase family the catalytic activity of which can be activated by Cyclin I (*CCNI*), but also non‐cyclin co‐activators p35 (*CDK5R1*) and p39 (*CDK5R2*) (Lowman *et al*, 2010; Sharma & Sicinski, 2020). We analyzed gene expression data from AML patients in The Cancer Genome Atlas dataset (Cancer Genome Atlas Research Network *et al*, 2013) and found that *CDK5*, *CCNI*, and *CDK5R1* are robustly expressed, whereas *CDK5R2* mRNA could not be detected in the majority of samples (Appendix Fig S6B). In competition assays, *CCNI*‐targeting sgRNAs similarly sensitized AML cells to AG636 treatment, whereas *CDK5R1* knockout had no effect (Figs 7B and EV5B). Thus, our data implicate CDK5/CCNI as synthetic lethal partners with DHODH in AML.

To explore how the loss of CDK5 altered the response of AML cells to AG636 we performed RNAseq in MOLM13 CDK5 knockout and control cells using a modified DRUG‐seq protocol (Ye *et al*, 2018). In multi‐dimensional scaling (MDS) analysis, samples were clustered by genotype and drug treatment. While all AG636‐treated samples were separated from vehicle‐treated samples in the first dimension, CDK5 knockout cells separated from control cells in the second dimension, indicating divergent transcriptional responses to DHODH inhibition (Fig 7C). AG636‐induced comparable downregulation of translation genes in both CDK5 knockout and control cells and AHA incorporation assays confirmed similar levels of inhibition of nascent protein synthesis (Fig EV5C and D). We then sought to identify other pathways that may explain the increased sensitivity of CDK5 knockout cells to drug treatment. There were 350 upregulated and 363 downregulated genes in CDK5 knockout cells that were not differentially expressed in control cells (Fig 7D). Network analysis revealed that these genes were highly enriched for multiple GO terms, including terms related to the electron transport chain, mitochondrial gene expression, DNA replication, and mRNA catabolism (Fig 7E). Of note, numerous transcripts encoding components of mitochondrial complex I and II including NDUFS1, NDUFB11, and SDHD were specifically downregulated in CDK5 knockout cells by AG636 treatment. Given the essential role of DHODH in the mitochondrial electron transport chain, it is tempting to speculate that the synthetic lethal relationship between DHODH and CDK5 is underpinned by synergistic mitochondrial targeting.

We then tested whether AG636 could synergize with CDK5 inhibitors. Most existing CDK5 inhibitors show poor selectivity for CDK5 over other cyclin‐dependent kinases due to their high sequence homology. We used Roscovitine, an ATP‐competitive inhibitor that has preferential activity against CDK5, but also blocks CDK1, 2, 7, and 9 (Meijer *et al*, 1997; Cicenas *et al*, 2015), and performed drug synergy assays in AML cell lines. Co‐treatment with AG636 and Roscovitine greatly increased cell death, especially in MLL‐rearranged MN and MOLM13 cells where Roscovitine alone had only mild effects (Fig 7F). Given that CDK5 expression is dispensable in these cells, whereas CDK1, 2, 7, and 9 are common essential genes required for the proliferation of almost all cancer cells lines (Appendix Fig S6C), the effects of Roscovitine are highly likely to be attributable to inhibition of CDK5. These results provide further evidence that CDK5 is a biomarker for the efficacy of DHODH inhibition in AML and provide proof of principle for a novel combinatorial strategy.

## Discussion

Many tumor cells reprogram their metabolism to meet altered demands for macromolecules and energy. Small molecules that interfere with DNA or RNA synthesis have been extensively used in cancer chemotherapy, demonstrating that although these processes are ubiquitous, they can nonetheless be targeted for therapeutic benefit. Critically, however, improvements to patient outcomes require a more sophisticated strategy. To that end, studies over the past decade have sought to identify selective metabolic vulnerabilities of cancer cells. Notably, an altered metabolic state appears to be a common feature of AML LSCs that is broadly conserved across different genetic contexts and is emerging as a potential Achilles’ heel in leukemia (Lagadinou *et al*, 2013; Jones *et al*, 2018, 2020; Pollyea *et al*, 2018).

The discovery linking pyrimidine starvation to cell fate in AML has reinvigorated interest in DHODH as an anticancer target and has spurred extensive efforts to develop more potent and selective next‐generation inhibitors (Sykes *et al*, 2016; Cao *et al*, 2019; Christian *et al*, 2019; McDonald *et al*, 2020; Zhou *et al*, 2020). In agreement with previous studies, we found that DHODH inhibition has excellent potency in different AML subtypes *in vivo*. The results were particularly striking in the highly aggressive MLL‐rearranged MN model, where most conventional or targeted agents induce only partial short‐lived responses (Zuber *et al*, 2009; Baker *et al*, 2016; Gilan *et al*, 2016). In contrast, AG636‐induced long‐lasting remission in almost all treated mice and these responses were sustained even after drug withdrawal. Drug treatment caused a mix of cell death and differentiation of leukemic cells leading to a rapid reduction in tumor burden and loss of LSC activity as confirmed by both morphological analysis and functional assays. The relative proportion of cell death and differentiation varied between the different AML subtypes and even between different compartments within individual animals, suggesting that phenotypic differentiation may not be an effective biomarker of drug response in all contexts.

The use of syngeneic tumor models enabled us to directly compare the sensitivity of malignant and nonmalignant hematopoietic cells to DHODHi *in vivo*. DHODH is widely expressed in healthy tissues and AG636 caused a rapid drop in myeloid progenitors and B cells in the bone marrow and progressive reduction in platelets, circulating lymphoid and myeloid cells. Notably, however, mice recovered after treatment was ceased, and within 4 weeks, there were no differences between AG636 or vehicle groups in any mature, progenitor, or stem populations. AG636 was also well‐tolerated in mice that received 3.5 Gy of whole‐body irradiation prior to tumor engraftment. Altogether, these findings confirm the existence of a therapeutic window for AG636 and suggest that DHODHi may be utilized sequentially after other cytotoxic therapies, although careful clinical management will likely be required to manage myelosuppression and/or thrombocytopenia.

Integrated analysis of gene expression data from genetically distinct LSCs identified downregulation of translation genes as a prominent transcriptional signature following DHODHi treatment. Further investigation revealed that AG636 had potent effects on ribosome biogenesis and protein synthesis. Mechanistically, our data suggest that the transcription factor YY1 and the associated INO80 chromatin remodeling complex play important roles in this molecular response. The response is triggered at least partly because inhibition of pyrimidine synthesis downregulates protein O‐GlcNAcylation leading to loss of YY1 protein. O‐GlcNAc is a common modification that regulates the stability, trafficking, and function of many proteins and OGT, the sole enzyme known to catalyze O‐GlcNAcylation, is indispensable for the proliferation of all 975 cancer cell lines in the DepMap database (Hart *et al*, 2007; Meyers *et al*, 2017). Indeed, DOT1L and MYC protein levels were also significantly depleted by AG636. Both DOT1L and MYC have been shown to be regulated by O‐GlcNAc and MYC has a well‐known role in ribosome biogenesis (van Riggelen *et al*, 2010; Sykes *et al*, 2016; Song *et al*, 2021). Hence, it is likely that the global loss of O‐GlcNAc, downstream of DHODHi exerts multiple antiproliferative effects. Moreover, other pathways including the ISR/ATF4 axis likely contribute to the potency of DHODHi in AML.

Our targeted CRISPR screen also uncovered a hitherto unknown relationship between inhibition of pyrimidine synthesis and CDK5. CDK5 has been most extensively characterized in the nervous system and has been implicated in the pathogenesis of Alzheimer’s and Parkinson’s diseases (Sharma & Sicinski, 2020). More recently, numerous studies have described a role for CDK5 in non‐neuronal cells including in AML where it was shown to regulate the pro‐apoptotic protein NOXA following glucose deprivation (Lowman *et al*, 2010). Our study demonstrates that genetic or pharmacological ablation of CDK5 increases DHODHi efficacy in AML. As loss of chromosome 7 (−7/7q) that encodes CDK5 is a poor prognostic marker in myeloid malignancies, our results open the door to prioritize clinical evaluation of DHODHi in a challenging patient population for which there are currently limited therapeutic options (Honda *et al*, 2015).

In conclusion, our study provides novel insights into the activity of DHODH inhibitors in AML and adds to the growing body of evidence supporting the further development of these compounds. Importantly, the response biomarkers that we have identified can aid these efforts and ensure that clinical trials are appropriately powered.

## Materials and Methods

### Animal experiments

All animal experiments were conducted in approved premises nominated on the Bureau of Animal Welfare Scientific Licence SPPL20183 (Agriculture Victoria, Australia) and were approved by the Peter MacCallum Cancer Centre Animal Experimentation Ethics Committee (Permit Number: E627). Congenic *Ptprca* (C57BL/6.SJL‐Ptprca) mice were purchased from the Animal Resource Centre (Western Australia) and maintained under specific pathogen‐free conditions. The MN and RUNX1‐RUNX1T1 AML models were described previously (Zuber *et al*, 2011; Bots *et al*, 2014). The I1DN model was generated by co‐transduction of fetal liver‐derived HSPCs with constructs encoding *IDH1^R132H^*, *DNMT3A^R882H^*, and *Nras^G12D^* (Gruber and Kats, unpublished). To generate murine leukemias, cryopreserved cells from the spleen or bone marrow of a moribund mouse were thawed and 5 × 10^5^ viable cells were transplanted into *Ptprca* recipients. For MN and I1DN experiments, animals were preconditioned by sublethal irradiation (3.5 Gy or 5.5 Gy, respectively) administered as a single dose prior to transplant. For drug treatment, AG636 (McDonald *et al*, 2020) was administered b.i.d. by oral gavage at a dose of 100 mg/kg body weight. For survival studies, the treatment took place on days 1–5 of a 7‐day cycle. Doxycycline was administered *ad libitum* via doxycycline‐supplemented food and water. Peripheral blood counts were performed on a Sysmex Cell Sorter (Sysmex). Mice were euthanized at predetermined time points or ethical endpoints based on clinical symptoms.

### Flow cytometry analysis and sorting

Single‐cell suspensions of whole blood, bone marrow, or spleen cells were incubated in ACK red cell lysis buffer (150 mM NH_4_Cl, 10 mM KHCO_3_, 0.1 mM EDTA) for 2 min and then washed in FACS buffer (PBS, 2% FBS). For blood, the lysis procedure was repeated a second time to ensure the efficient removal of mature red blood cells. Cells were then resuspended in FACS buffer and stained with fluorophore‐conjugated antibodies targeted against cell surface markers (detailed list of antibodies is provided in Table EV2). Normal mouse bone marrow cells were used as a comparator, and count beads were added to each sample after staining to enable quantification of cell numbers. Flow cytometric analysis was performed on FortessaX20 or Symphony flow cytometers (BD Biosciences), and data were analyzed using FlowJo software (Tree Star). Cell sorting was performed Fusion 5 flow cytometer (BD Biosciences).

### Cell culture

MN cells from the spleens of tumor‐bearing mice were cultured in Anne Kelso modified Dulbecco's modified Eagle's medium (Low glucose DMEM (Invitrogen), 4 g/l glucose, 36 mg/l folic acid, 116 mg/l l‐arginine HCl, 216 mg/l l‐glutamine, 2 g/l NaHCO_3_, and 10 mM HEPES), supplemented with 20% fetal bovine serum (FBS) (Invitrogen), 0.1 mM l‐Asparagine (Sigma‐Aldrich), and 1% penicillin/streptomycin (Invitrogen). Cells were maintained at 37°C and 10% CO_2_. OCI‐AML3 and MOLM13 were purchased from the DSMZ. HEK293T, MV4‐11, and Kasumi‐1 cells were purchased from ATCC. OCI‐AML3, MOLM13, and MV4‐11 were cultured in RPMI‐1640 (Gibco) supplemented with 20% FBS, 1% pen/strep, and 100 µM Glutamax (Invitrogen) at 37°C and 5% CO_2_. HEK293T cells were cultured in DMEM (Gibco) supplemented with 10% FBS and 1% pen/strep. All cell lines in this study were routinely tested for *Mycoplasma* contamination. Cells were treated as indicated with AG636, PUGNAc (Sigma‐Aldrich), Cytarabine (Hospira), JQ1 (Bradner Lab), SGC0946 (Sigma‐Aldrich), Roscovitine (Assay Matrix), or DMSO (Sigma‐Aldrich). For the combination therapy, experiment drugs were refreshed every second day and cells were passaged every 2–4. To quantify the viable cells, cells were stained with 0.2 µg/ml DAPI for 15 min and analyzed by flow cytometry with CountBright™ Absolute Counting Beads (ThermoFisher).

### Lentiviral constructs and transduction

Cas9‐mCherry (Addgene Plasmid #70182) was used to generate stable Cas9‐expressing cell lines. sgRNA sequences listed in Dataset EV5 were cloned into the lentiGuide‐Crimson backbone (Addgene Plasmid # 70683). Nonreplicating lentiviruses were generated by transient co‐transfection of the transfer plasmids into HEK293T together with the packaging plasmids pMDL (Addgene Plasmid #12251), pRSV‐REV (Addgene Plasmid #12253), and pVSVG (Addgene Plasmid #12259). Lentivirus containing supernatants were harvested and transduced into AML cell lines with 4 µg/ml sequabrene (Sigma‐Aldrich).

### Western blotting

Western blotting was performed according to standard laboratory protocols. Cells were lysed in RIPA lysis buffer (50 mM Tris–HCl pH 8, 150 mM NaCl, 1% NP‐40, 0.5% sodium deoxycholate, 0.1% SDS, 0.5 U benzonase, and protease inhibitors) for 30 min at 4°C. 30 µg protein lysates were resolved in 4–15% Mini‐PROTEAN TGX Precast Protein Gels (Bio‐Rad) and immunoblotted onto Immobilon‐P PVDF membrane (Millipore). Membranes were incubated with primary and secondary antibodies, and near‐infrared Western blot detection was performed by the Odyssey CLx imaging system (LI‐COR Biosciences). The following antibodies were used: YY1 (D5D9Z) Rabbit mAb (1:1,000, Cell Signaling #46395), O‐GlcNAc (CTD110.6) Mouse mAb (1:1,000, Cell Signaling # 9875), β‐actin (1:5,000, Sigma‐Aldrich #A2228), IRDye^®^ 680RD Goat anti‐Mouse IgG (H + L) (1:20,000–40,000, LI‐COR Biosciences #926‐68070), IRDye^®^ 800CW Goat anti‐Rabbit IgG (H + L) (1:20,000–40,000, LI‐COR Biosciences #926‐32211).

### Proliferation assays

1 × 10^6^ cells were seeded at a density of 5 × 10^5^ cells per ml and treated with 100 nM or 250 nM AG636, in combination with 50 μM PUGNAc (Sigma‐Aldrich, A7229) or 100 μM Uridine (Sigma‐Aldrich U3003). The viable cell number was determined by Trypan blue exclusion using Countess 3 Automated Cell Counter (Thermo Fisher Scientific).

### RNAseq and bioinformatic analysis

Sorted cKit^high^CD11b^low^ AML cells were lysed in Trizol reagent, and RNA was extracted using Direct‐zol RNA miniprep kit (Zymo Research) according to the manufacturer’s instructions. RNA sequencing was performed at the Peter MacCallum Cancer Centre Molecular Genomics core facility. The QuantSeq 3′ mRNA‐seq Library Prep Kit for Illumina (Lexogen) was used to generate libraries as per the manufacturer’s instructions. Libraries were pooled and sequenced with 75 bp single‐end sequencing to a depth of 10 × 10^6^ reads on a NextSeq500 (Illumina). Sequencing reads were demultiplexed using bcl2fastq (v2.17.1.14), and low‐quality reads Q < 30 were removed. The RNA sequencing reads were trimmed at the 5′ end using cutadapt (v1.14) to remove bias introduced by random primers used in the library preparation, and 3′ end trimming was performed to eliminate poly‐A‐tail derived reads. Reads were mapped to the reference genome (mm10) using HISAT2. Reads were counted using subread software package (v1.6.3). Differential gene expression analysis was performed using R package LIMMA (v6.5). R packages pheatmap (v1.0.12) and ggplot2 (v3.2.1) were used for figure generation. CAMERA (Wu & Smyth, 2012) was used for competitive testing of C2 curated gene sets from the Broad Institute’s MSigDB for the mouse. GSEA (v4.0.1) was used for analyzing the enrichment of gene sets and gene ontology (GO) analysis was performed using metascape (http://metascape.org).

### qPCR

RNA was extracted as outlined above. Reverse transcription was performed with the High‐Capacity cDNA Reverse Transcription Kit (ThermoFisher) using 1 µg total RNA. qPCR was performed using SensiFast SYBR Hi‐ROX kit (Bioline) on CFX96 Touch Real‐Time PCR System (Bio‐Rad). Results were analyzed using the 2^ΔΔC(t)^ method using β‐actin or β‐2‐microglobulin for normalization. Sequences of primers used are listed in Dataset EV5.

### ATACseq and bioinformatic analysis

ATACseq was performed as described previously using (Buenrostro *et al*, 2013) using sorted 5 × 10^4^ cKit^high^CD11b^low^ MN cells. Briefly, cells were permeabilized using 10 mM Tris–HCl pH 7.4, 10 mM NaCl, 3 mM MgCl_2_, 0.1% IGEPAL CA‐630, 0.1% Tween‐20, and tagmentation was performed using the Tagment DNA TDE1 Enzyme and Buffer kit (Illumina). Tagmented chromatin was purified using the ChIP DNA Clean & Concentrator kit (Zymo Research). Libraries were pooled and sequenced with 75bp single‐end sequencing to a depth of 20 × 10^6^ reads per sample on a NextSeq500 (Illumina). Raw sequencing reads were demultiplexed using bcl2fastq (v2.17.1.14) and quality control performed with FastQC (v0.11.5). Adaptor sequences were removed using Trim Galore! (v0.4.4) and reads aligned to the mouse genome (mm10) using Bowtie2 (v2.3.3). Samtools (v1.4.1) was used for processing SAM and BAM files including the removal of duplicate reads. Peaks were called using Macs2 (v2.1.1) with no lambda and no model settings. In R (v 4.0.2), CSAW (v1.18.0) was used to count reads in windows specified by the union of vehicle‐ and drug‐treated Macs2 peaks, filter blacklisted regions (ENCODE), and perform loess normalization, then edgeR (v3.32.1) was used for differential accessibility analysis (Reske *et al*, 2020). ChIPseeker (v1.26.0) and TxDB. Mmusculus.UCSC.mm10.knownGene (v3.10.0) was used for annotation of peaks to gene features. HOMER (v4.8) was used for motif discovery. Bamtools (v2.4.1) was used to merge replicate BAM files. BAM files were converted into BigWig files using the bamCoverage function (Deeptools, v3.5.0) using the following settings (‐‐normalizeUsing CPM ‐‐smoothLength 150 ‐‐binSize 50 ‐e 200). Average profile plots were generated by computing read average read density (from BigWig files) across defined genomic intervals using computeMatrix and visualized using plotProfile (Deeptools, v3.5.0).

### PSCAN analysis

PSCAN was used to identify statistically over‐represented transcription factor motifs in the proximal promoters of translation genes (Zambelli *et al*, 2009). The analysis was performed with a window of 500 bp (−450 and +50 bp relative to TSS), using Jaspar 2018_NR TFBSs matrices. PSCAN can be accessed at: http://159.149.160.88/pscan/

### Ribosome profiling

1 × 10^7^ MN cells were seeded at 5 × 10^5^ per ml and treated with AG636 (250 nM) for 24 h. 1 × 10^7^ viable MN cells were used per biological replicate. Polysome profiling experiments were performed in cells pretreated with 100 µg/ml cycloheximide (Sigma‐Aldrich, C‐1988) for 5 min. Cells were washed with ice‐cold PBS followed by hypotonic wash buffer (5 mM Tris–HCl (pH 7.5), 2.5 mM MgCl_2_, 1.5 mM KCl) containing 100 µg/ml cycloheximide. Cells were lysed in a hypotonic lysis buffer (5 mM Tris–HCl (pH 7.5), 2.5 mM MgCl_2_, 1.5 mM KCl, 100 µg/ml cycloheximide, 2 mM DTT, 0.5% Triton X‐100, and 0.5% sodium deoxycholate) on ice for 10 min and lysates were precleared by centrifugation to remove nuclei. The cytoplasm was collected and loaded onto a 10–40% linear sucrose density gradient (containing 20 mM Tris–HCL (pH 7.6), 100 mM KCl, 5 mM MgCl_2_) and centrifuged at 36,000 rpm [SW40 Ti rotor (Beckman Coulter, inc)] for 2.15 h at 4°C. Gradients were fractionated (14 fractions per sample), and optical density was continuously recorded at 260 nm using a sensitivity of 1 on an ISCO Tris and UA‐6 UV/VIS detector (Teledyne).

### AHA incorporation assay

Nascent protein synthesis was quantified using the Click‐iT AHA Alexa Fluor 488 Protein Synthesis Assay (Thermo Fisher, C10289) according to the manufacturer’s instructions with modifications. Briefly, 1 × 10^6^ MN cells were seeded in 1 ml media and treated with 250 nM AG636, 50 µM PUGNAc, 100 µM uridine or DMSO for 23 h, or 100 ng/ml cycloheximide for 1 h. Cells were then centrifuged, washed with PBS, and cultured in AHA‐supplemented methionine free media for 1 h. Cells cultured without AHA served as a negative control. Cells were then fixed in 4% paraformaldehyde at room temperature for 15 min and permeabilized in 0.5% Triton X‐100 for 15 min. Cells were stained according to the manufacturer’s instructions and analyzed by FACS.

### ChIPseq enrichment analysis

ENCODE ChIPseq data are processed using the ENCODE Transcription Factor ChIPseq processing pipeline to generate binding peaks for each chromatin‐associated factor (ENCODE Project Consortium, 2012). ChIPseq enrichment scores for transcription factors expressed in all three AML models at the promoter regions of selected genes (+1,000 bp to −50 bp from TSS) were extracted using the UCSC genome browser (http://genome.ucsc.edu/index.html) based on the ENCODE data. If more than two scores for a TF at a particular promoter region were present, only the highest score was included for subsequence calculation. Average enrichment score was calculated for all genes within a particular gene set: translation genes (*n* = 24), AG636 downregulated genes (*n* = 707), and other genes (*n* = 4237).

### ChIP‐qPCR

ChIP was performed as described previously with modifications (Fan *et al*, 2020). Briefly, cells were resuspended in PBS and crossed‐linked with formaldehyde solution (50 mM Hepes‐KOH pH 7.5, 100 mM NaCl, 1 mM EDTA, and 10% (v/v) formaldehyde) at room temperature for 10 min. Excess formaldehyde was quenched by the addition of glycine to 125 mM for 5 min. Cross‐linked cells were washed once with ice‐cold PBS and were then washed three times with nuclear extraction buffer (20 mM Tris–HCl pH 8, 10 mM NaCl, 2 mM EDTA, and 0.5% (v/v) IGEPAL CA‐630) containing protease inhibitors (Merck, 04693159001). Nuclear extracts were resuspended in sonication buffer (20 mM Tris–HCl pH7.5, 150 mM NaCl, 2 mM EDTA, 1% (v/v) IGEPAL CA‐630, and 0.3% (w/v) SDS) containing protease and phosphatase inhibitors (Thermo Fisher Scientific, A32957) and were sonicated at maximum power for 12 min using the Covaris S220 Focused Ultrasonicator. Sonicated lysates were diluted with one volume of ChIP dilution buffer (20 mM Tris–HCl pH 8, 150 mM NaCl, 2 mM EDTA, and 1% (v/v) Triton X‐100) containing protease and phosphatase inhibitors. 5% of the lysates were collected as input. Protein A and protein G Dynabeads were washed in blocking buffer (20 mM Tris–HCl pH 8, 150 mM NaCl, 2 mM EDTA, 1% (v/v) Triton X‐100, 0.15% (w/v) SDS, and 0.1% (w/v) bovine serum albumin) containing protease inhibitors at 4°C. Protein A/G beads were resuspended in ChIP IP buffer (20 mM Tris–HCl pH 8, 150 mM NaCl, 2 mM EDTA, 1% (v/v) Triton X‐100, 0.15% (w/v) SDS) and added to nuclear lysate with 3 μg of anti‐YY1 or IgG control antibodies and 0.5% BSA. Samples were incubated overnight at 4°C with rotation. Beads were then washed twice with ChIP IP buffer before washing with ChIP wash buffer 1 (20 mM Tris–HCl pH 8, 500 mM NaCl, 2 mM EDTA, 1% (v/v) Triton X‐100, 0.1% (w/v) SDS), and wash buffer 2 (20 mM Tris–HCl pH 8, 250 mM LiCl, 2 mM EDTA, 0.5% (v/v) IGEPAL CA‐630, 0.1% (w/v) SDS, 0.5% (w/v) sodium deoxycholate), each containing protease and phosphatase inhibitors, and washing twice with Tris–EDTA buffer (10 mM Tris–HCl pH 7.5 and 1 mM EDTA). Washed beads were incubated with reverse crosslinking buffer (1% (w/v) SDS, 100 mM NaHCO_3_, and 200 mM NaCl) containing 300 μg of proteinase K (Sigma‐Aldrich, P2308) at 55°C for 1 h before incubation of the supernatant at 65°C overnight. DNA was isolated using the ChIP DNA Clean & Concentrator Kit (Zymo Research, D5205). qPCR was performed using SensiFast SYBR Hi‐ROX kit (Bioline) on CFX96 Touch Real‐Time PCR System (Bio‐Rad). Results were analyzed using input for normalization, with percent input = 5% × 2^(Ct Input‐Ct IP)^, and further normalized to the DMSO condition. Primers used are listed in Dataset EV5.

### Epigenetics‐targeted CRISPR screen

The screen was performed as described previously with modifications (Doench *et al*, 2016). We used a custom sgRNA library containing guides targeting 859 epigenetic regulator (four sgRNAs/gene) genes and 100 nontargeting controls (guide sequences are provided in Dataset EV6). To generate the library, guide sequences were PCR amplified from a CustomArray Inc oligo pool and cloned into the lentiGuide‐Puro (Addgene #52963) backbone using Golden Gate cloning. For the screen, 6 × 10^6^ MN^Cas9^ cells were transduced with MOI < 0.3 to achieve single sgRNA integration per cell at an average 500‐fold representation. Two days post‐viral infection, transduced cells were selected by 2 µg/ml Puromycin for 3 days. A time point 0 pellet of 2 × 10^6^ live cells was harvested by centrifugation, snap‐frozen, and stored at −80°C until required. To identify resistance or sensitization to AG636, samples were divided into three groups at the 5 days (T0) post‐selection: Vehicle (1:1,000 DMSO), 100 nM AG636, and 250 nM AG636. Drug/vehicle was refreshed every second day and cells passaged every 2–3 days, maintaining at least 500× representation by seeding 2 × 10^6^ cells in 10 ml every passage. Samples were harvested at T10 and T24 and snap‐frozen. Genomic DNA was extracted using the DNeasy Blood & Tissue Kit (Qiagen). Libraries were prepared by nested PCR method as described previously (Doench *et al*, 2016), pooled, purified using AMPure XP beads (Beckman Coulter), and sequenced to a depth of two million reads with single‐end 75 bp sequencing on a NextSeq500 (Illumina). Sequencing reads were demultiplexed using bcl2fastq (v2.17.1.14), and low‐quality reads Q < 30 were removed. The reads were trimmed using cutadapt (v1.16.5)^7^ to extract the 20 bp targeting sequence and sgRNAs that were enriched or depleted in response to AG636 relative to T0 and DMSO determined using the MAGeCK algorithm (v0.5.8.1) (Li *et al*, 2014). R packages ggplot2 (v2.2.1) and ggrepel (v0.8.0) were used for figure generation.

### Competitive proliferation assay

sgRNAs constructs in the lentiGuide‐Crimson backbone were transduced into Cas9‐expressing AML cell lines at 30–70% efficiency. Cells were treated with vehicle (1:1,000 DMSO) or AG636 (250 nM or 1 µM) beginning on day 4 post‐transduction. The drug was refreshed every second day and cells passaged every 2–3 days. The percentage of sgRNA‐expressing (Crim^+^) cells were measured by flow cytometry and normalized to the initial transduction efficiency.

### DRUG‐Seq and bioinformatic analysis

Sequencing was performed using a modified version of DRUG‐Seq. Briefly, 1 × 10^6^ MN cells were seeded at 0.5 × 10^6^ per ml and treated with treated with 250 nM AG636 or DMSO for 24 h. Cells were then counted, and 20,000 cells were transferred into V‐bottom plate, centrifuged, washed with 200 µl ice‐cold PBS, centrifuged again, and removed washing. 17 µl lysis buffer were added into each well and incubated at room temperature for 15 min under agitation (900 rpm). 12.5 µl of cell lysate were transferred into each well of a new 96‐well plate previously prepared with 1 µl of 10 nM well‐specific DRUG‐Seq RT primer and 7.5 µl RT mix. The mixture was incubated for 2 h at 42°C to create well‐barcoded full‐length cDNA, and then, all the wells of a plate were combined into a single tube. Pooled cDNA was concentrated and purified with the DNA Clean and Concentrator kit (Zymo) followed by Ampure XP beads (Beckman Coulter) with each plate eluted in 22 µl nuclease‐free water. The purified cDNA was preamplified with KAPA HiFi HotStart ReadyMix (Roche) and DRUG‐Seq PreAmp PCR primer, and the quality was checked on a D5000 Screentape (TapeStation, Agilent). One barcoded library was prepared per plate by shearing the preamplified material on the Covaris S2 platform and then using the NEBNext Ultra II DNA Library Prep Kit (NEB) to end‐repair and ligate adapters to the fragmented molecules. The library was then amplified and indexed using NEBNext Ultra II Q5 Master Mix (NEB), i7 Index Primer (NEB), and DRUG‐Seq P5 PCR Primer. Libraries were purified with DNA Ampure XP beads (Beckman Coulter), quality checked on a DNA1000 tape (TapeStation, Agilent), and quantity verified by qPCR. Two indexed libraries were sequenced on a NextSeq 500 instrument (Illumina) using a custom sequencing primer (DRUG‐Seq Read primer) and a High Output Kit v2.5 75 Cycles (Illumina) with paired‐end configuration (26 base pairs for read 1 and 60 base pairs for read 2).

Reads were aligned using STARsolo (v 2.7.5b) (‐‐soloType CB_UMI_Simple ‐‐soloCBstart 1 ‐‐soloCBlen 10 ‐‐soloUMIstart 11 ‐‐soloUMIlen 10 ‐‐soloBarcodeReadLength 25 ‐‐soloCBwhitelist $whitelist) to a concatenated hg38/dm6 reference. The raw output file was imported into R (v 4.1.0) using the Read10× function from Seurat (v 4.1.0) and dm6 genes filtered out. This counts file is supplied in Dataset EV7. Lowly expressed counts were filtered using edgeR’s (v 3.34.0) function filterByExpr (min.count = 5, min.total.count = 10) and differential gene expression performed using Limma (v 3.48.3). edgeR was used to generate the MDS plot on the top 500 most variable genes after rlog transformation (DESeq2 v 1.32.0). clusterProfiler (v 4.0.5) and enrichplot (v 1.12.3) were used to generate the network plot of enriched GO terms. Specifically, DEGs induced by AG636 (FDR < 0.05) that were specific to sgCDK5 cells (and not sgSCR) were used for GO analysis with clusterProfiler (enrichGO with default parameters). The GO terms were then used as input for enrichplot functions pairwise_termsim (showCategory = 500) followed by treeplot (showCategory = 30, nCluster = 6, hclust_method = “ward.D2”) in order to identify the top clusters of GO terms. Network plots of the enriched GO terms were generated using the ggraph (v2.0.5) and enrichplot function cnetplot, with GO term nodes color coded according to their cluster.

### Statistical analysis

GraphPad Prism 9.0 and R version 3.6.2 software were used for statistical analysis. Statistical tests performed for each experiment are highlighted in the figure legends.

## Author contributions

**Lev M Kats:** Conceptualization; Data curation; Supervision; Funding acquisition; Methodology; Writing—original draft; Project administration; Writing—review & editing. **Joan So:** Data curation; Investigation; Methodology; Writing—original draft; Project administration; Writing—review & editing. **Alexander C Lewis:** Data curation; Investigation; Methodology; Writing—review & editing. **Lorey K Smith:** Investigation; Methodology. **Kym Stanley:** Investigation; Methodology. **Rheana Franich:** Investigation; Methodology. **Lizzy Pijpers:** Resources; Investigation. **Pilar M Dominguez:** Resources; Methodology. **Simon J Hogg:** Methodology. **Stephin Vervoort:** Methodology. **Ricky W Johnstone:** Supervision; Methodology; Writing—review & editing. **Gabrielle McDonald:** Methodology. **Danielle B Ulanet:** Methodology. **Josh Murtie:** Methodology. **David Yoannidis:** Investigation; Methodology. **Fiona C Brown:** Methodology. **Emily Gruber:** Formal analysis; Investigation; Methodology; Writing—review & editing.

In addition to the CRediT author contributions listed above, the contributions in detail are:

JS, ACL, LKS, KS, RF, EG, and LMK performed the experiments and analyzed the data. JS, ACL, EG, and LMK wrote the manuscript. LMK designed the study. DY, LP, PD, SJH, SJV, FCB, RWJ, GM, DBU, and JM provided essential reagents and technical expertise. All authors discussed the results and approved the manuscript.

## Disclosure and competing interests statement

LMK has received research funding and consultancy payments from Agios Pharmaceuticals Celgene Corporation and Servier Pharmaceuticals. GM is an employee of Servier Pharmaceuticals. DBU and JM were employees of Agios Pharmaceuticals.

## Supporting information



Appendix

Expanded View Figures PDF

Dataset EV1

Dataset EV2

Dataset EV3

Dataset EV4

Dataset EV5

Dataset EV6

Dataset EV7

Table EV1

Table EV2

Source Data for Expanded View and Appendix

Source Data for Figure 1

Source Data for Figure 2

Source Data for Figure 3

Source Data for Figure 4

Source Data for Figure 5

Source Data for Figure 6G

Source Data for Figure 7

## Data Availability

RNA‐, ATAC‐, and CRISPR‐sequencing data presented in this study have been deposited into the Gene Expression Omnibus hosted at the National Center for Biotechnology under the accession number GSE181666 (https://www.ncbi.nlm.nih.gov/geo/query/acc.cgi?acc=GSE181666). All other data are available from the corresponding author on request.
