# Supplementary material for: Inhibition of pyrimidine biosynthesis targets protein translation in acute myeloid leukemia
Source: EMBO Mol Med. 2022 May 6;14(7):e15203. doi: 10.15252/emmm.202115203 (PMC9260210; doi:10.15252/emmm.202115203)
Supplement: Supplementary file 1 — Appendix [file EMMM-14-e15203-s009.pdf]

Appendix

Table of contents

Appendix Figure S1

Appendix Figure S2

Appendix Figure S3

Appendix Figure S4

Appendix Figure S5

Appendix Figure S6

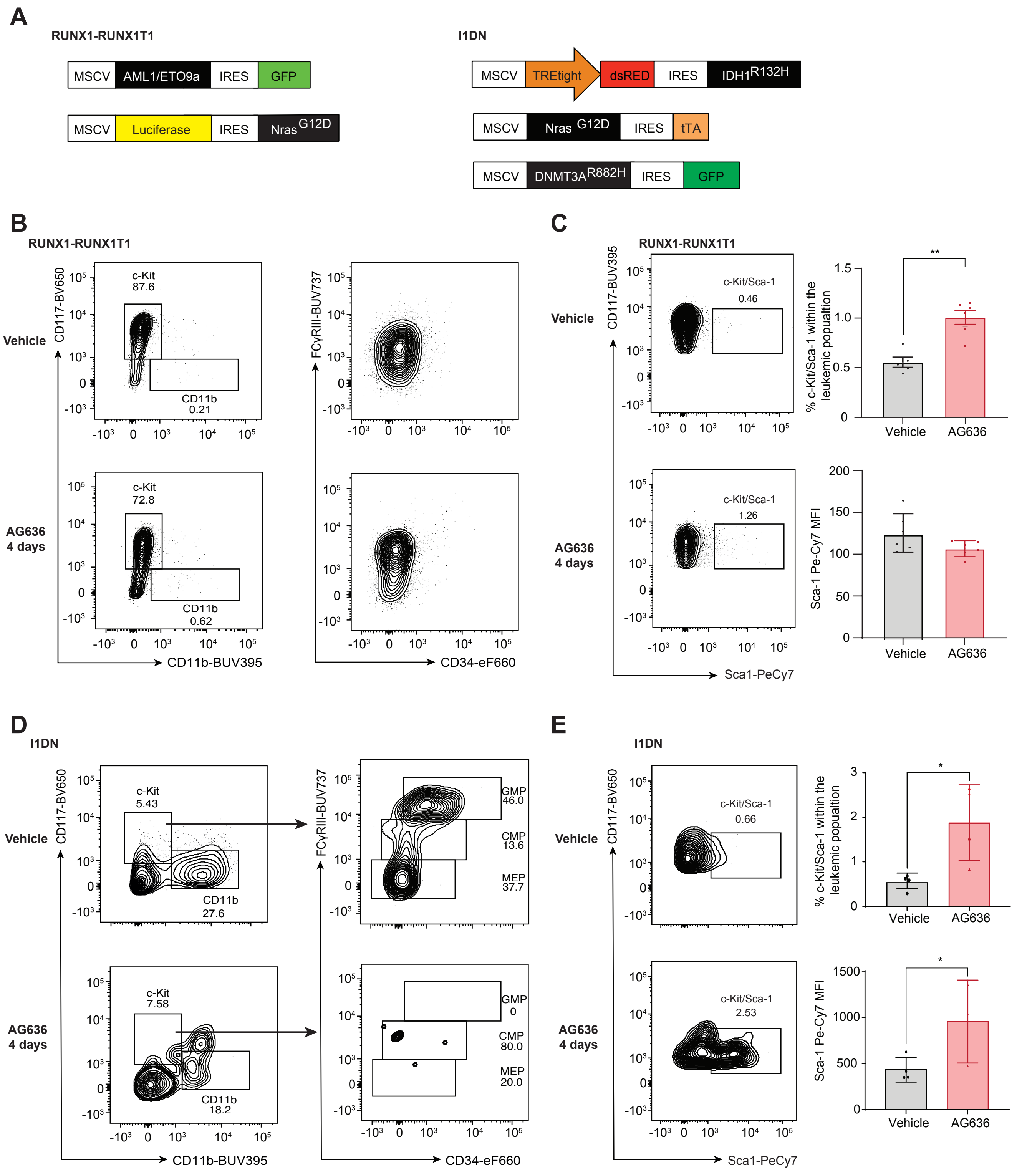

Appendix Figure S1. Efficacy of DHODH inhibition in the RUNX1-RUNX1T1 and I1DN murine AML models.

A Schematic of RUNX1-RUNX1T1 and I1DN models.

B-E Representative FACS plot of the bone marrow of RUNX1-RUNX1T1 (B and C) or I1DN (D and E) tumor bearing mice showing expression of the indicated cell surface markers. Quantification of Sca1 expression is shown.

Data information: data are presented as mean ± s.d.; *P* values were calculated using a one-tailed Student's unpaired t-test. $*P < 0.05$.

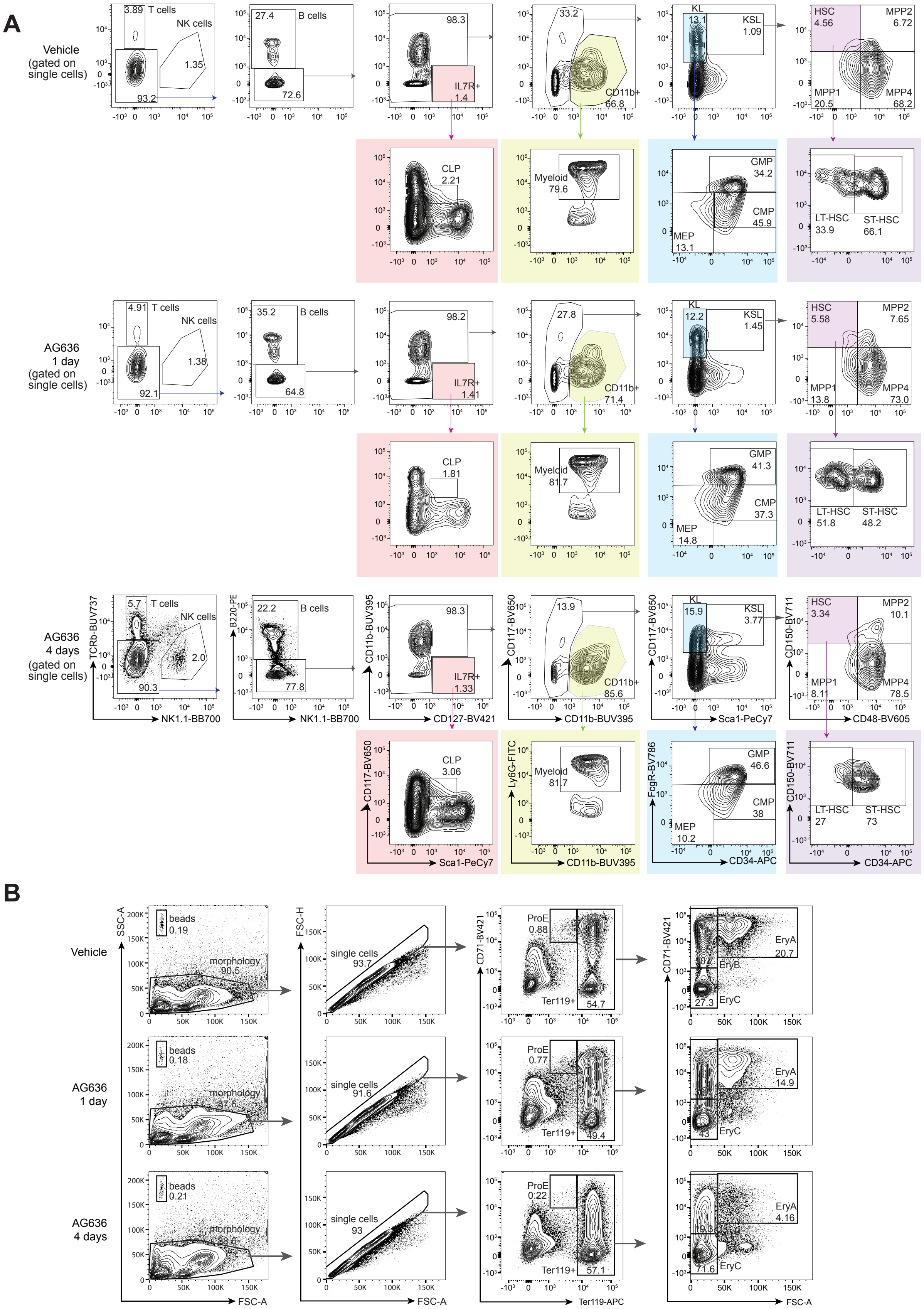

Appendix Figure S2. Identification of bone marrow sub-population by FACS.

A FACS gating strategy for bone marrow mononuclear cells (MNCs).

B FACS gating strategy for erythroid cells.

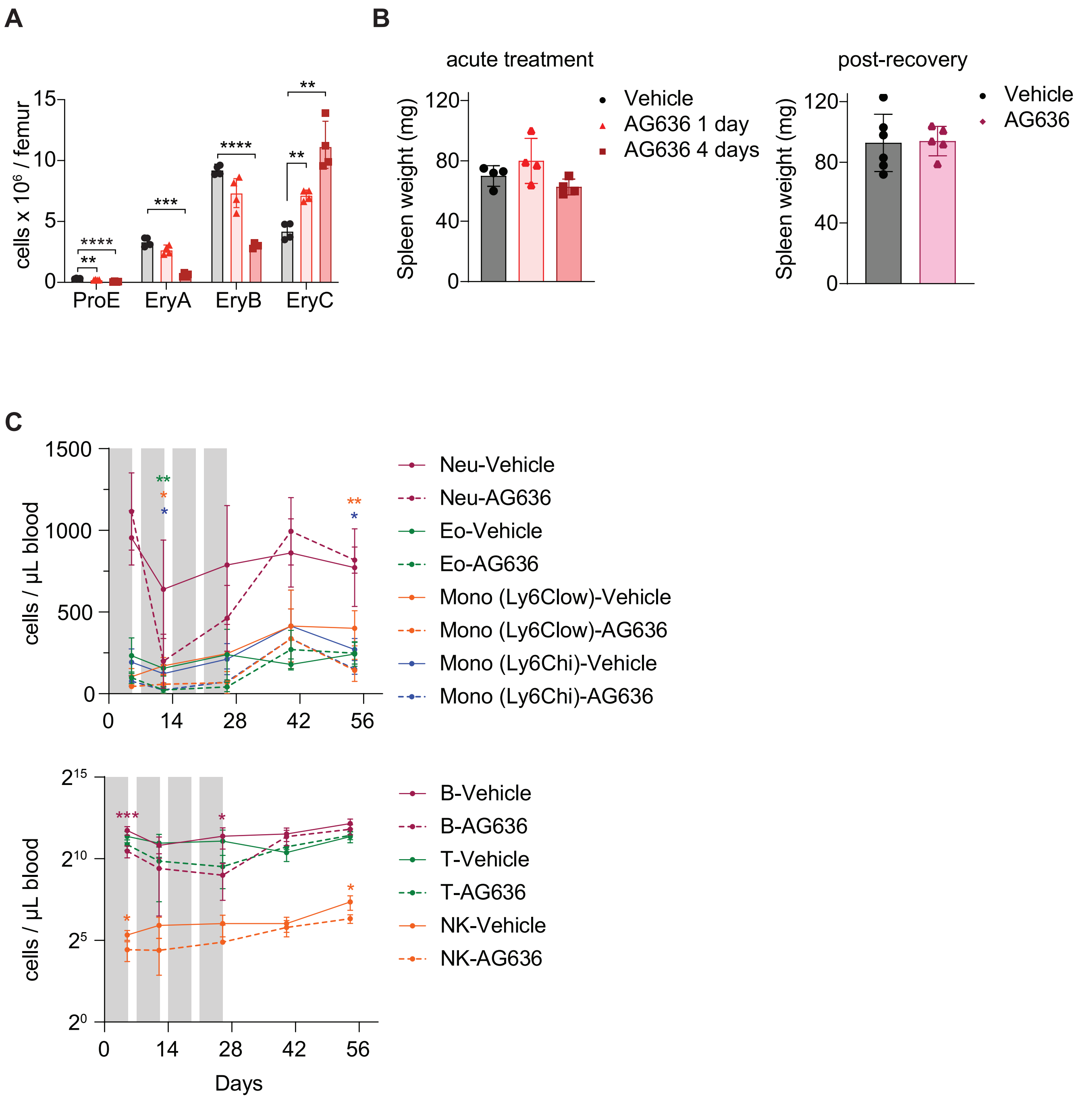

Appendix Figure S3. Effects of AG636 on normal hematopoiesis.

A Erythroid cells in the bone marrow quantified by flow cytometry in mice treated with AG636 or vehicle for 1 or 4 days.

B Spleen weight in mice treated with AG636 or vehicle for 1 or 4 days (acute treatment) or for 4 cycles followed by 4 weeks off treatment (post-recovery).

C Number of various myeloid (top) and lymphoid (bottom) cells in the peripheral blood of mice treated with AG636 or vehicle. Grey bars denote treatment.

Data information: n = 4 mice/group for acute treatment, n = 6 mice for prolonged treatment and recovery; data are presented as mean ± s.d.; P values were calculated using a two-tailed Student's unpaired t-test; only comparisons that meet the threshold of $P < 0.05$ are shown. *$P < 0.05$, **$P < 0.01$, ***$P < 0.001$; see Table EV1 for abbreviations and markers.

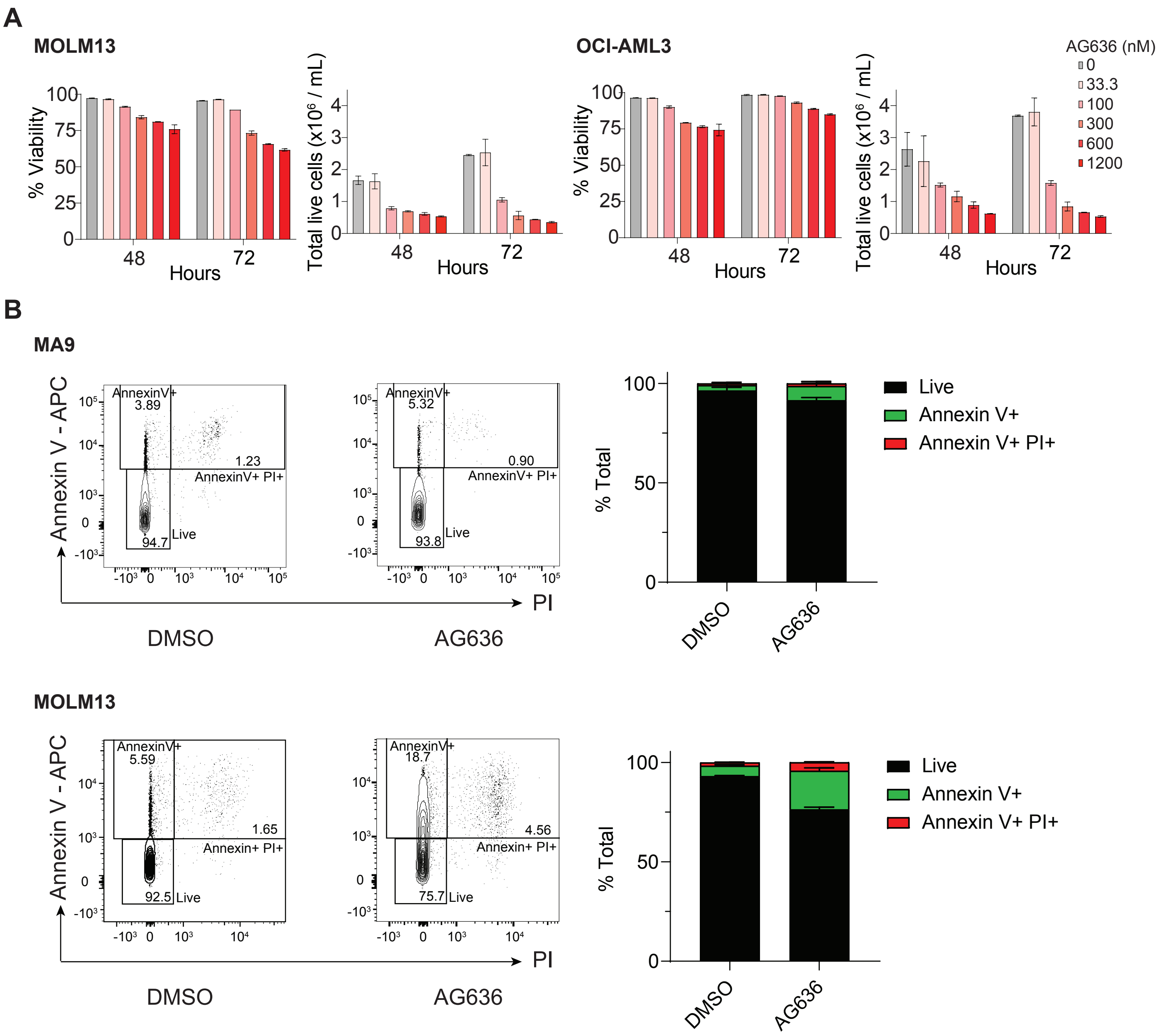

Appendix Figure S4. Effects of AG636 treatment on gene expression.

A Cell viability of human AML cell lines treated with AG636.

B Cells were treated with AG636 or vehicle for 24 hours, stained with Annexin V and propidium iodide (PI) and analyzed by FACS. Representative FACS plots and quantification are shown.

Data information: n = 2 biological replicates in B, n = 3 biological replicates in C; data are presented as mean ± s.d.

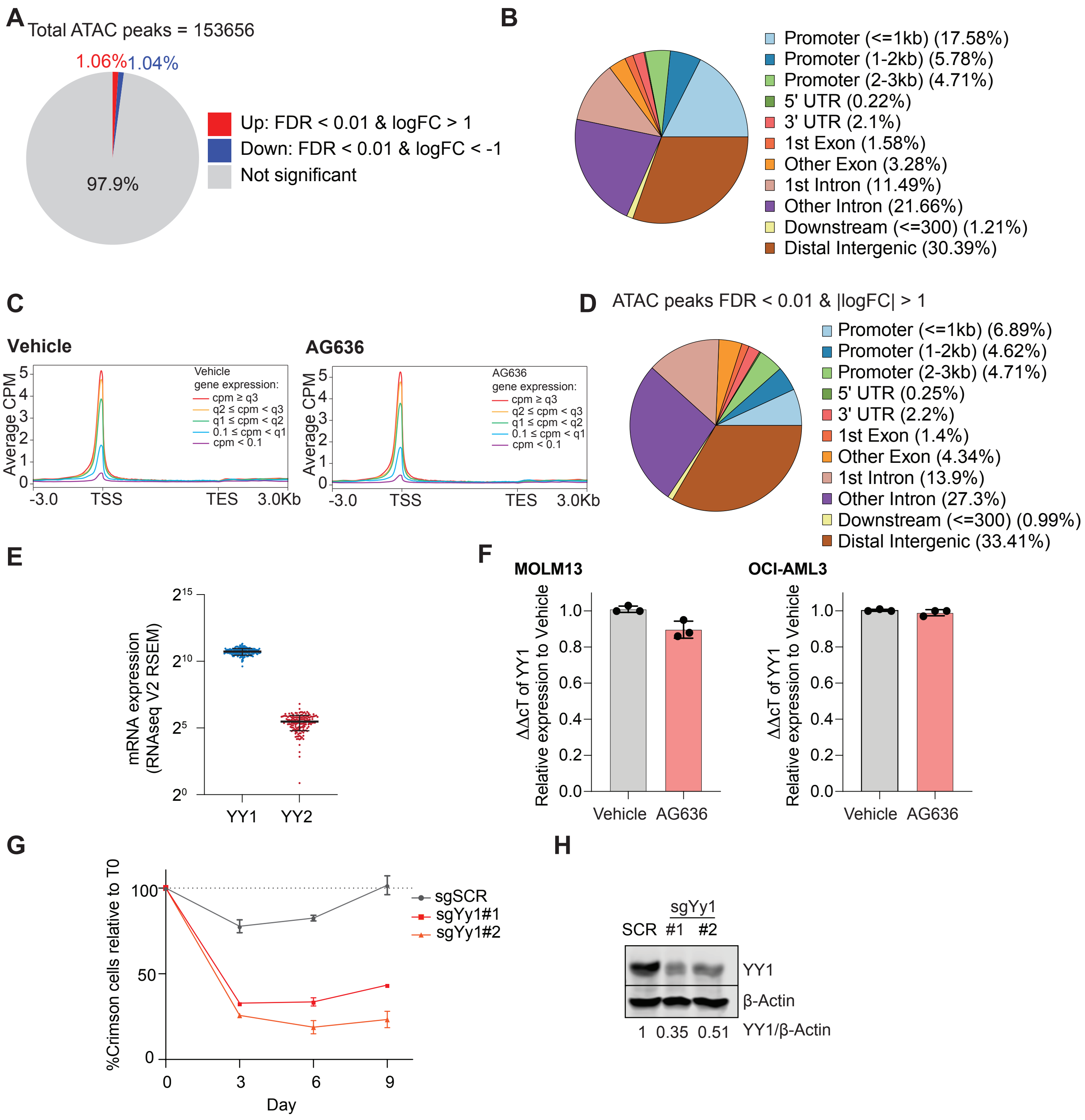

Appendix Figure S5. Identification of transcription factors that contribute to the AG636 transcriptional response.

A Pie chart showing the relative proportion of ATAC peaks where chromatin accessibility is increased, decreased or unchanged by DHODH inhibition.

B Pie chart showing association between regions of accessible chromatin and different genome features in untreated MN cells.

C Metaplot of ATACseq signal at genes grouped by gene expression. Quantile values were calculated on genes whose expression was ≥ 0.1 CPM.

D Pie chart showing association between regions of differential chromatin accessibility and different genome features.

E Expression of YY1 and YY2 in AML patient samples in the TCGA database (data represented as mean ± s.d.).

F qPCR of *YY1* expression in human AML cell lines treated with AG636 for 24 hours (n = 2 biological replicates; data represented as mean ± s.e.m).

G Proliferative competition assays in MN cells transduced with two independent YY1-targeting sgRNAs or a non-targeting control sgRNA (n = 2 biological replicates; data represented as mean ± s.e.m).

H Western blot of YY1 expression in MN cells transduced with two independent YY1-targeting sgRNAs or a non-targeting control sgRNA.

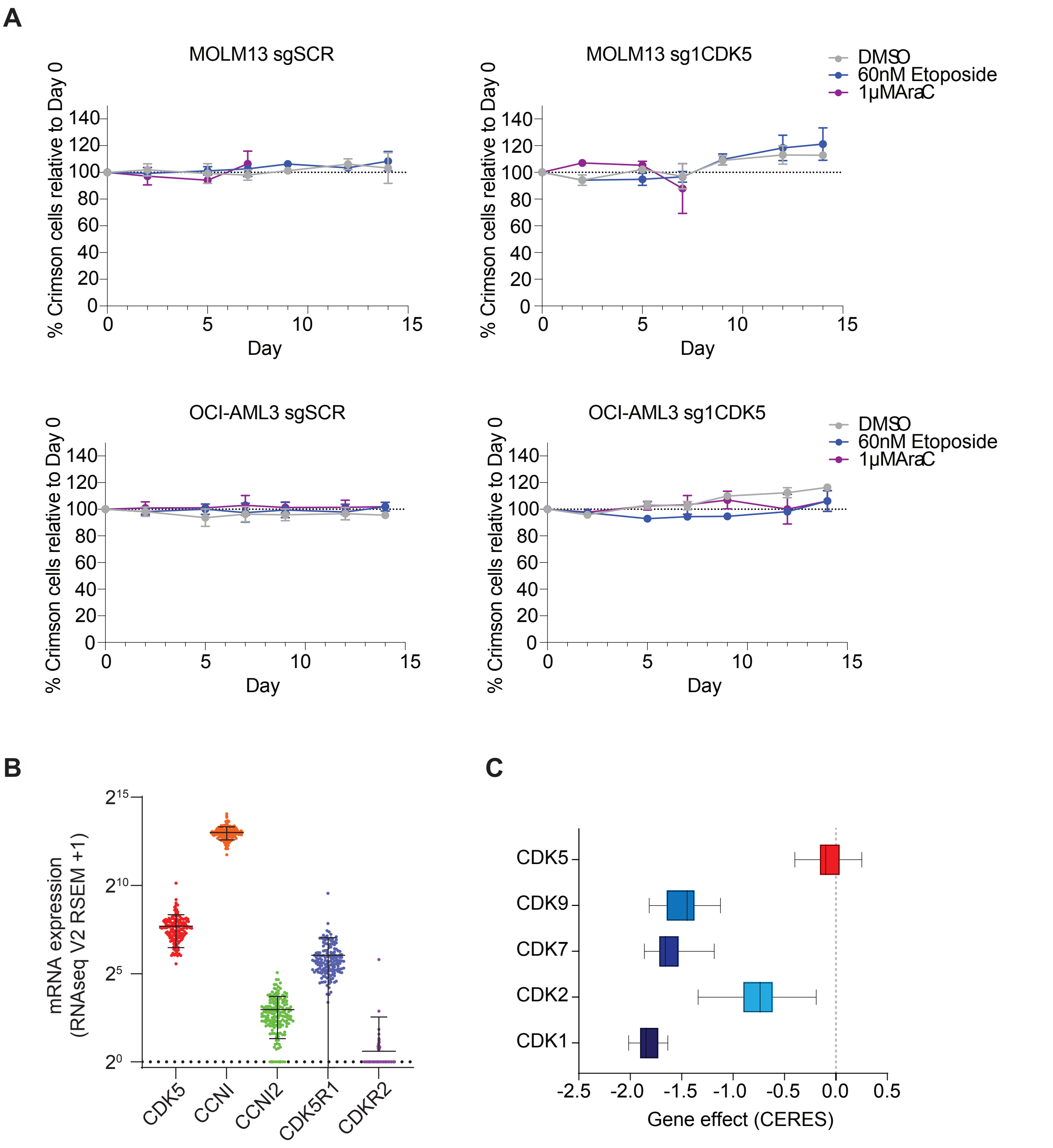

Appendix Figure S6.

A Proliferative competition assays in OCI-AML3 cells transduced with CDK5 targeting or scrambled sgRNAs and cultured in various inhibitors or DMSO (n = 2 biological replicates; data represented as mean ± s.e.m).

B Expression of *CDK5* and its activators in AML patient samples in the TCGA database (Cancer Genome Atlas Research Network *et al*, 2013) (data represented as mean ± s.d.).

C Gene dependencies in AML cell lines from the DepMap database (Meyers *et al*, 2017), Centre line; median; box limits, from the 25th to 75th percentiles; whiskers, from the 5th to 95th percentiles.
