# Supplementary material for: Inhibition of pyrimidine biosynthesis targets protein translation in acute myeloid leukemia
Source: EMBO Mol Med. 2022 May 6;14(7):e15203. doi: 10.15252/emmm.202115203 (PMC9260210; doi:10.15252/emmm.202115203)
Supplement: Supplementary file 2 — Expanded View Figures PDF [file EMMM-14-e15203-s013.pdf]

Expanded View Figures

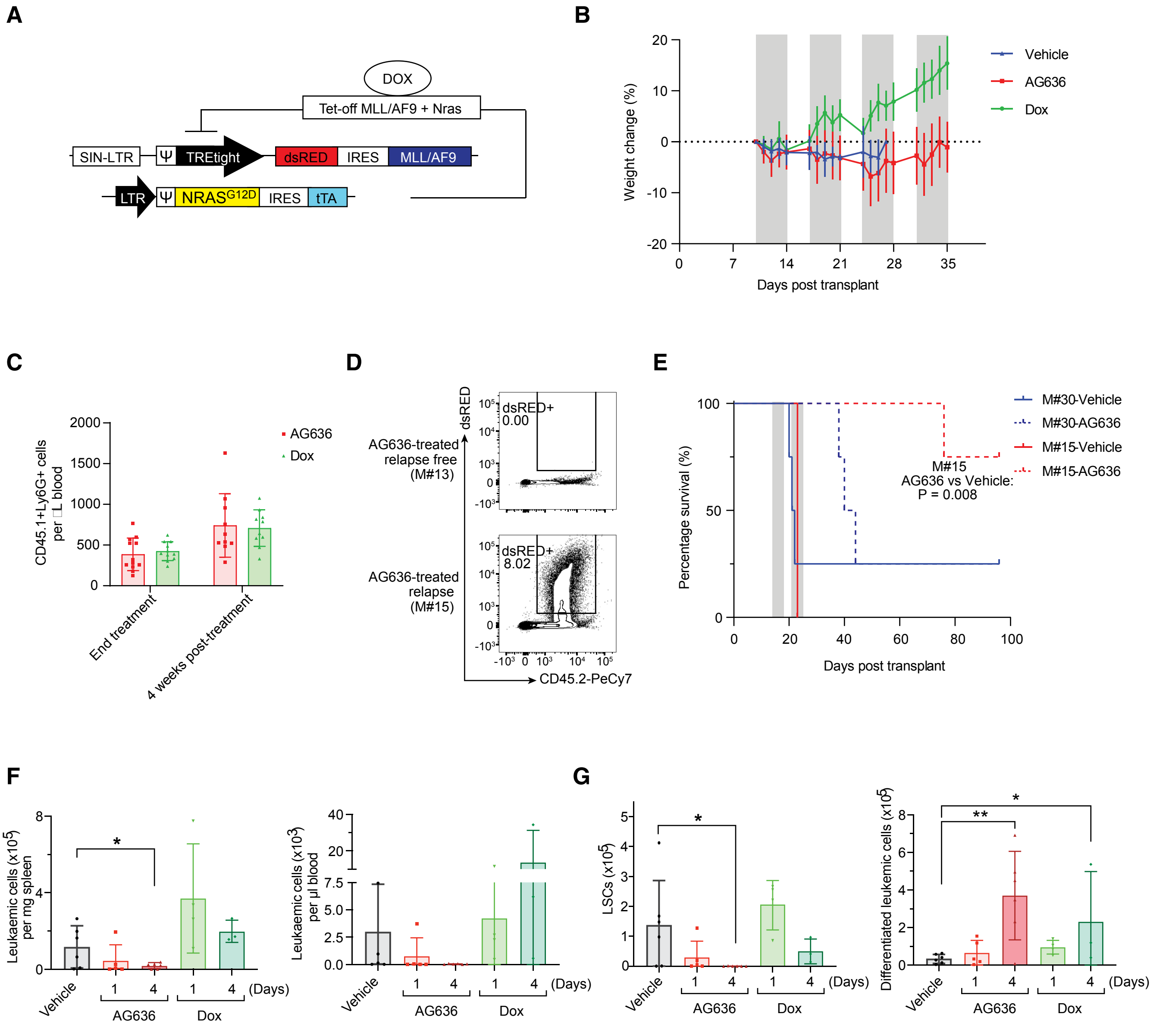

Figure EV1. Efficacy of DHODH inhibition in the MN murine AML model.

- A Schematic of MN model.
- B Body weight of MN tumor-bearing mice treated with AG636. Gray bars denote treatment. Dotted line defines zero percent weight loss.
- C Number of recipient-derived myeloid cells ($CD45.1^+CD11b^+Ly6G^+$) in the peripheral blood of AG636- or doxycycline-treated recipients at the conclusion of therapy and after 4 weeks (n = 8–10 mice/group).
- D Representative FACS plots of the bone marrow from a mouse with no detectable disease (M#13) and a relapsed mouse (M#15).
- E Kaplan–Meier survival curve of secondary recipients transplanted with leukemic cells from the relapsed donor (M#15) or a control donor from the vehicle group (M#30). Gray bars denote treatment (n = 4 mice/group, median survival is 21.5 for vehicle-treated M#30, 42 for AG636-treated M#30, 23 for vehicle-treated M#15, and not reached for AG636-treated M#15, the P value was calculated by log-rank test).
- F Number of MN cells in the spleen and peripheral blood quantified by flow cytometry (n = 3–6 mice/group).
- G Number of LSCs ($CD11b^{low}cKit^{high}FcgR^+$) and differentiated leukemic cells ($CD182^+Ly6G^+$) in the bone marrow (n = 3–6 mice/group).

Data information: data in F-G are presented as mean ± SD; P values were calculated using a one-tailed Student's unpaired t-test. *$P < 0.05$, **$P < 0.01$, Dox—doxycycline.

Source data are available online for this figure.

© 2022 The Authors

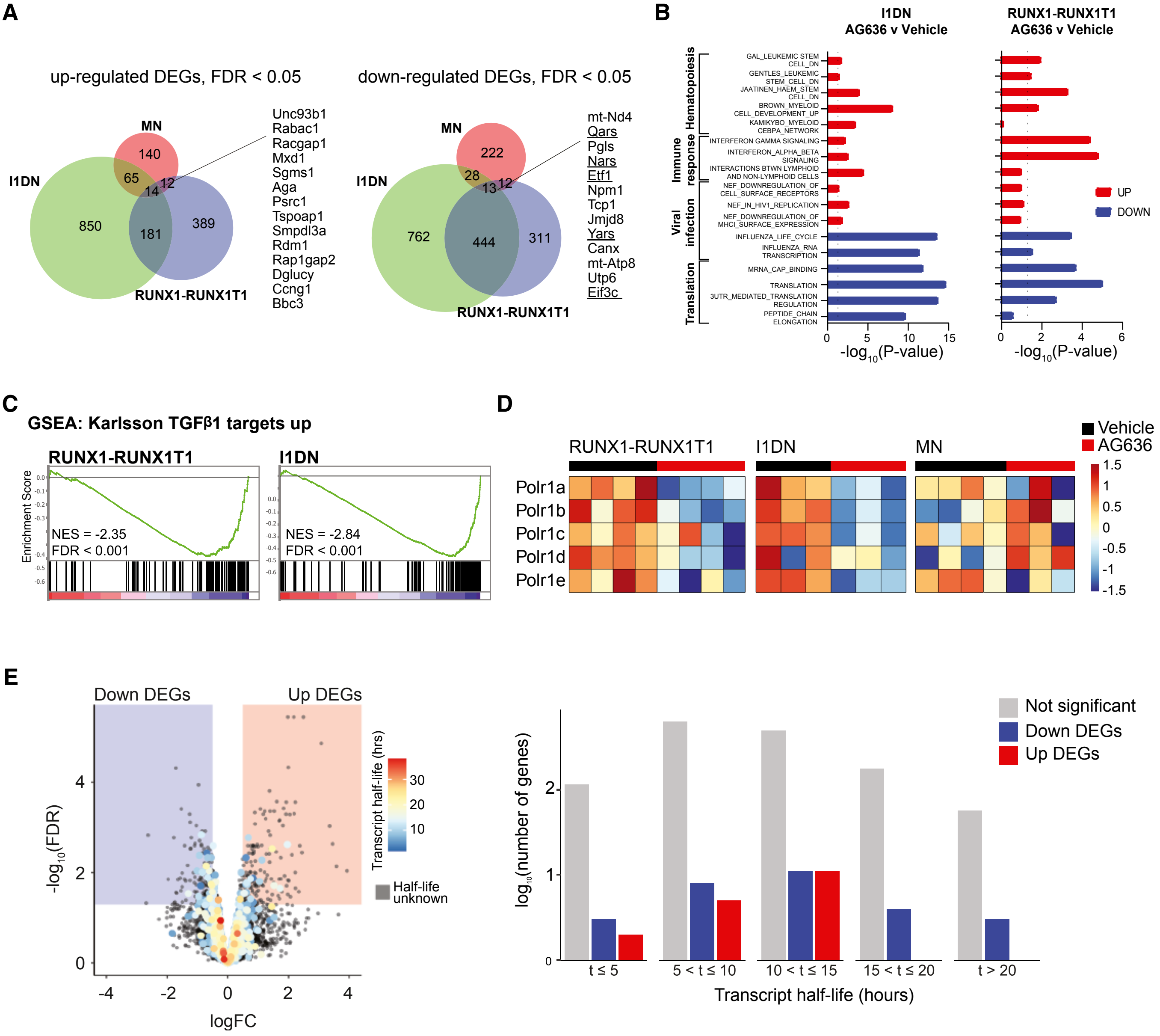

Figure EV2. Comparison of transcriptional effects of DHODHi in different AML models.

A Venn diagram showing the overlap in DEGs in the MN, RUNX1-RUNX1T1, and I1DN models.

B Gene set enrichment analysis showing the enrichment of selected biological pathways in gene expression data from RUNX1-RUNX1T1 and I1DN murine AML models following AG636 treatment. Gene sets are from C2:CGP and Reactome subcollections in MSigDB database (see methods for more information).

C Barcode plots showing downregulation of TGF-β signaling in RUNX1-RUNX1T1 and I1DN murine AML models following AG636 treatment.

D Gene expression heat map showing downregulation of genes encoding components of RNA polymerase I in RUNX1-RUNX1T1 and I1DN murine AML models following AG636 treatment.

E Volcano plot of gene expression in MN cells, highlighting the average transcript half-life of each gene (left) and bar chart of the number of genes with transcript half-lives in the given interval for significant DEGs (right). Transcript half-life was provided by (Schwanhäusser *et al*, 2011).

© 2022 The Authors

Figure EV3. YY1 is a downstream target of AG636 in AML.

A Western blot of ATF4, DOT1L, and MYC in MN cells treated with AG636 for 24 h.
B Violin plot of YY1 ChIPseq enrichment scores at the promoter regions (+1,000 bp to −50 bp from TSS) of genes within the indicated gene sets extracted from ENCODE 3 (n = 24 genes for translation genes, n = 707 genes for AG636 downregulated genes, n = 4,237 for the other genes).
C Screenshot from UCSC genome browser (http://genome.ucsc.edu/index.html) of YY1 enrichment at the promoter regions of translation genes RPL13A and PRL18A in 11 out of 12 cell lines in ENCODE3 data.
D Western blot for YY1 in MN cells co-treated with AG636 and PUGNAc for 24 h.
E qPCR showing the expression of translation genes in MOLM13 (right) or MN cells (left) co-treated with AG636 and PUGNAc or uridine for 24 h.
F Nascent protein synthesis quantified using the AHA incorporation assay in MN or MOLM13 cells co-treated with AG636 and PUGNAc or uridine for 24 h (n = 3 biological replicates).
G Proliferation assay in MN or MOLM13 cells co-treated with AG636 and PUGNAc or uridine for 24 h (n = 3 biological replicates).

Data information: data in E, F, and G are presented as mean ± SEM; P values were calculated using a two-tailed Student's unpaired t-test in B, a one-tailed Student's unpaired t-test in E, a one-way ANOVA with the Tukey's test for multiple comparisons in F, and a 2-way ANOVA with the Šídák's test for multiple comparisons in G; *$P < 0.05$, **$P < 0.01$, ***$P < 0.001$, ****$P < 0.0001$.

Source data are available online for this figure.

© 2022 The Authors

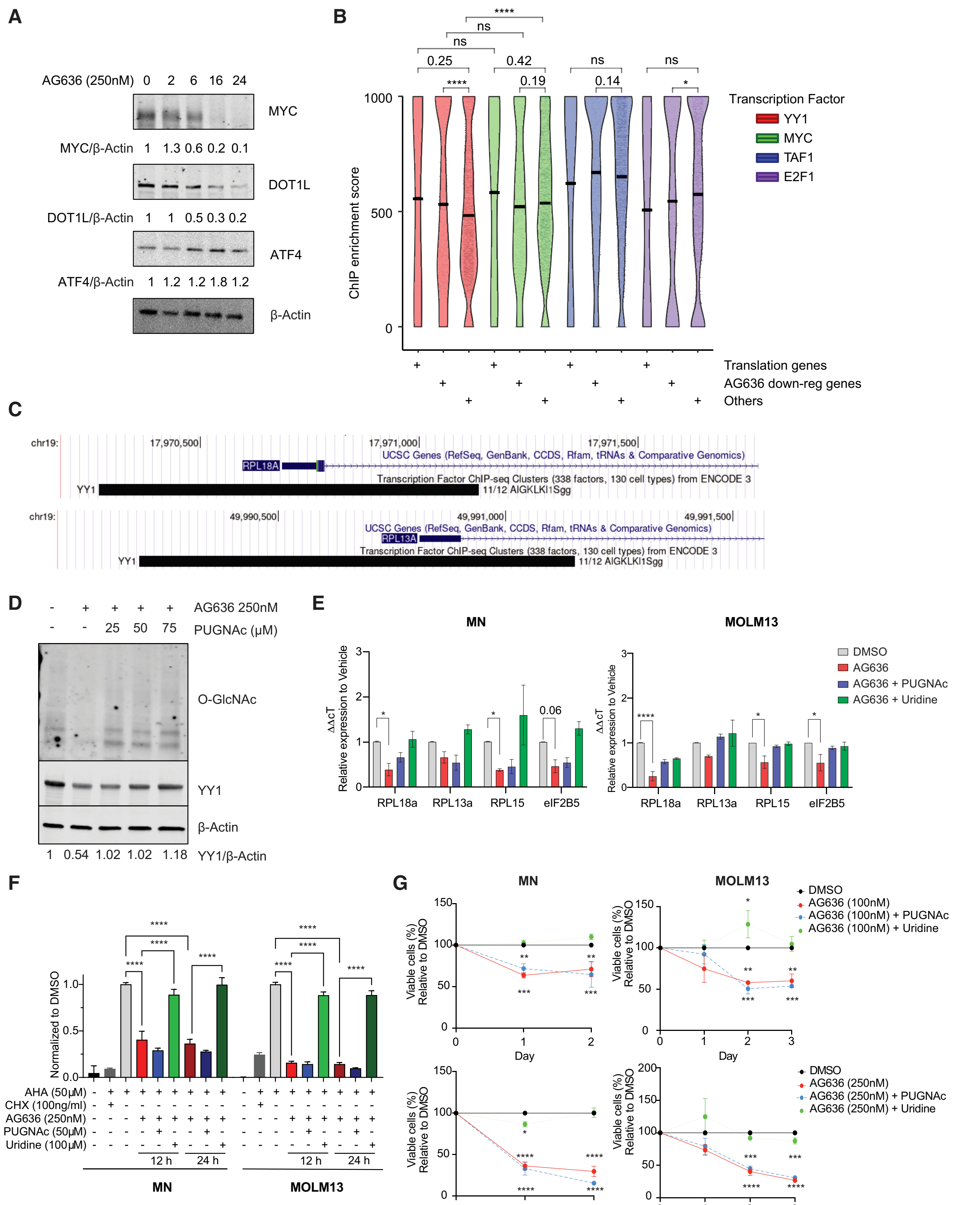

Figure EV3.

© 2022 The Authors

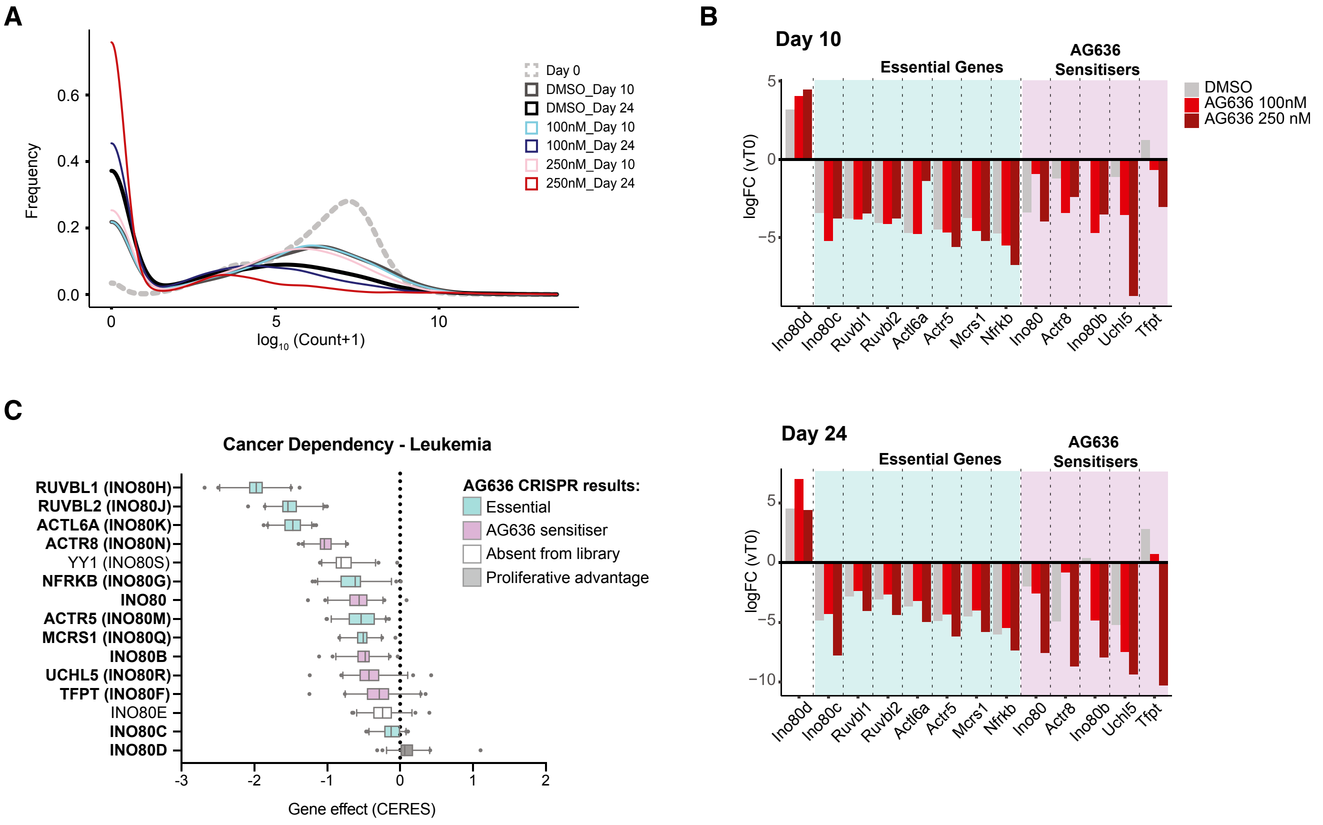

Figure EV4. Identification of genes that increase or decrease the sensitivity of AML cells to DHODHi.

A Distribution of sgRNA counts in the various conditions during the CRISPR screen.

B Average fold change in sgRNA counts at time point 10 (top) and at time point 24 (bottom) compared with time point 0 for all sgRNAs targeting components of the INO80 complex in the various conditions in the CRISPR screen.

C Gene dependencies in AML cell lines from the DepMap database (Meyers *et al*, 2017), center line; median; box limits, from the 25th to 75th percentiles; whiskers, from the 5th to 95th percentiles (n = 47 cell lines).

Source data are available online for this figure.

Figure EV5. CDK5/CCNI expression affects the response to DHODHi in AML.

A Proliferative competition assays in human AML cell lines transduced with CDK5-targeting or scrambled sgRNAs and cultured in various inhibitors or DMSO (n = 2 biological replicates). Dashed line defines no changes compared with time point 0.

B Proliferative competition assays in human AML cell lines transduced with CDK5R1 sgRNAs and cultured in AG636 or DMSO.

C Barcode plots showing downregulation of the Reactome Translation gene set in MOLM13 cells transduced with CDK5-targeting or scrambled sgRNAs and treated with AG636 or DMSO for 24 h.

D Nascent protein synthesis quantified using the AHA incorporation assay in MOLM13 cells transduced with CDK5-targeting or scrambled sgRNAs and treated with AG636 or DMSO for 24 h. MOLM13 cells treated with cycloheximide for 1 h or cultured in the absence of AHA served as controls (n = 3 biological replicates).

Data information: data in A and D are presented as mean ± SD; P values were calculated using a one-tailed Student's unpaired t-test in B; $*P < 0.05$, $**P < 0.01$.
Source data are available online for this figure.

© 2022 The Authors

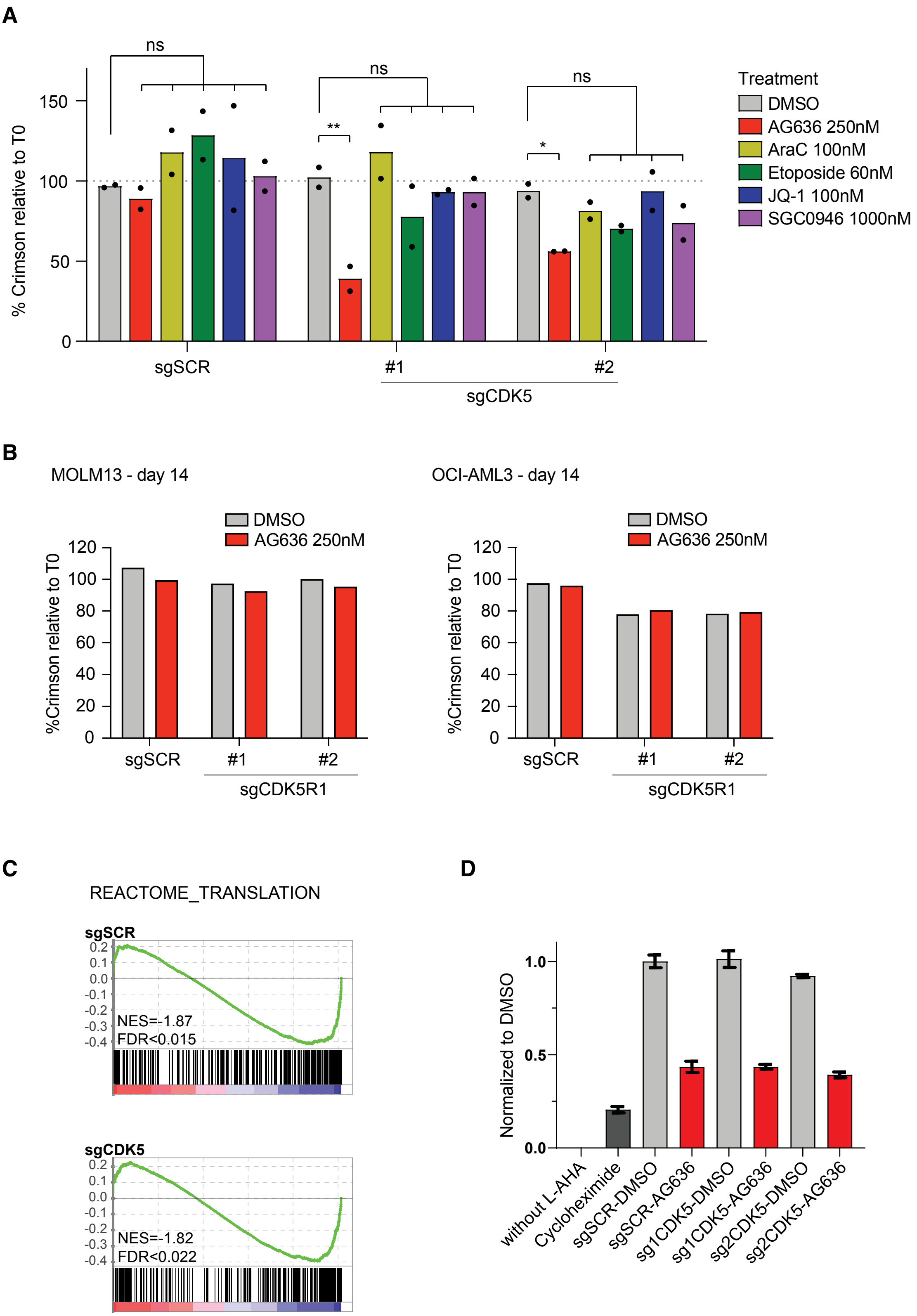

Figure EV5.

© 2022 The Authors
