# Supplementary material for: Inhibition of pyrimidine biosynthesis targets protein translation in acute myeloid leukemia
Source: EMBO Mol Med. 2022 May 6;14(7):e15203. doi: 10.15252/emmm.202115203 (PMC9260210; doi:10.15252/emmm.202115203)
Supplement: Supplementary file 20 [file EMMM-14-e15203-s006.pdf]

EMBO Molecular Medicine

Inhibition of pyrimidine biosynthesis targets protein translation in acute myeloid leukemia

Joan So, Alexander Lewis, Lorey Smith, Kym Stanley, Rheana Franich, David Yoannidis, Lizzy Pijpers, Pilar Dominguez, Simon Hogg, Stephin Vervoort, Fiona Brown, Ricky Johnstone, Gabrielle McDonald, Danielle Ulanet, Josh Murtie, Emily Gruber and Lev M. Kats
DOI: 10.15252/emmm.202115203

Corresponding author(s): Lev Kats (Lev.Kats@petermac.org)

Review Timeline:

Submission Date:	23rd Sep 21
Editorial Decision:	29th Oct 21
Revision Received:	29th Mar 22
Editorial Decision:	8th Apr 22
Revision Received:	12th Apr 22
Accepted:	13th Apr 22

Editor: Jingyi Hou

Transaction Report:

(Note: With the exception of the correction of typographical or spelling errors that could be a source of ambiguity, letters and reports are not edited. Depending on transfer agreements, referee reports obtained elsewhere may or may not be included in this compilation. Referee reports are anonymous unless the Referee chooses to sign their reports.)

Thank you for submitting your work to EMBO Molecular Medicine. We have now heard back from the three referees who evaluated your manuscript. As you will see from the reports below, the referees acknowledge the potential interest and relevance of the study. However, they also raise a series of concerns about your work, which should be convincingly addressed in a major revision of the present manuscript.

Referees shared overlapping concerns about the insufficient data supporting the conclusions regarding the impact of AG636 on translation and the role of YY1 in mediating the effects of AG636. We think that addressing these concerns is essential and would indeed significantly strengthen the conclusions of the manuscript.

All other issues raised by the referees need to be satisfactorily addressed as well. I understand that some of the issues raised by the reviewers require extensive additional analyses and we would be happy to extend the revision deadline, should you need additional time beyond the 90 days. Please note that EMBO Molecular Medicine strongly supports a single round of revision and that, as acceptance or rejection of the manuscript will depend on another round of review, your responses should be as complete as possible.

EMBO Molecular Medicine has a "scooping protection" policy, whereby similar findings that are published by others during review or revision are not a criterion for rejection. Should you decide to submit a revised version, I do ask that you get in touch after three months if you have not completed it to update us on the status.

We are aware that many laboratories cannot function at full efficiency during the current COVID-19/SARS-CoV-2 pandemic and have therefore extended our "scooping protection policy" to cover the period required for a full revision to address the experimental issues. Please let me know should you need additional time, and also if you see a paper with related content published elsewhere.

***** Reviewer's comments *****

Referee #1 (Remarks for Author):

The manuscript entitled "Inhibition of pyrimidine biosynthesis targets protein translation in AML" by Joan So and colleagues point to the possibility of limiting protein translation by inhibiting DHODH, and thus propose it as a potential new therapeutic target for the treatment of AML. The authors first demonstrated the effects of DHODH inhibition in leukemia progression in mouse models for MLL rearranged leukemia. They further validate the findings using models of non MLL rearranged leukemia. In addition, they tested the effect of DHODH inhibition in normal hematopoiesis. To understand the molecular mechanism of the observed therapeutic effects, they performed transcriptomic analyses and identified genes involved in protein translation that were downregulated after inhibition of DHODH, including YY1, a transcriptional regulator of protein translation. By performing a CRISPR targeted screen focused on epigenetic modifiers, they identified gene candidates which rescue DHODH inhibition as well as ones showing synthetic lethality.
The paper is well written and the experimental design as well as the data are very well presented. However, the paper is mainly focusing on the analysis of the transcriptional effects of DHODH inhibition rather than deeply characterizing protein translation as claimed in the title. Additional experimental data should be provided to directly (rather than indirectly by transcriptional analysis) examine the connection between DHODH inhibition and protein translation. To some degree, the novelty is affected as the role of DHODH as a potential therapeutic AML target was already reported (Sykes et al., Cell 2016; Christian et al., Leukemia 2019; Gaidano et al., Cancers 2021)). Nevertheless, this is an interesting, important and technically well performed study.

Prior to publication, the following issues should be addressed with the goal of further improving the manuscript:

(1) In extended Fig. 1 it is shown the overall survival of secondary recipients transplanted with either naïve (M30) or leukemia cells derived from primary mice showing leukemia relapse (M15). While M15 show high sensitivity to DHODH inhibition excluding the possibility of acquired resistance, DHODH inhibitor treated M30 with did not show prolonged survival as shown in Fig. 1. Please comment on this and if required, repeat the experiment by including more secondary recipients obtained from naïve primary leukemia mouse models.
(2) Fig. 3 shows the effect of DHODH inhibition on normal hematopoiesis. Data are shown after 1 or 4 days of treatment. In contrast, the treatment of leukemic mice followed a treatment scheme of 4 cycles of 5 days each. It would be important to assess the effect on normal hematopoiesis using the same treatment scheme. In addition, the authors should perform a time course experiment in order to assess the time needed for the normal hematopoietic system to recover from this treatment.
(3) Fig. 4 addresses the transcriptional changes induced by DHODH inhibition. Transcriptomic data are generated in all the leukemia mouse models. It would be highly interesting to determine the common DEGs between all these leukemic mouse models treated similarly.
(4) Differentially expressed genes important for protein translation are validated in vitro, as well as in AML cell lines and by

ribosome profiling. Based on the changes shown in the ribosome profiling, one would expect a massive block in protein translation. Is the amount of total proteins/cell different between AG636 and vehicle treated cells?
(5) The authors identified YY1 as the transcription factor candidate mediating the effect of AG636 and suggest an involvement of O-GlcNAc modifications. To confirm these data, the authors analyzed expression levels of genes involved in protein translation after YY1 KD. Among the many candidates, why was YY1, which is only marginally affected by AG636, was chosen to be followed up? The specific effect of YY1 in the control of protein translation gene expression is not convincing. Transcriptomic data after YY1 KD and comparison to AG636 treated cells should be provided. Furthermore, YY1 ChIP data proving the enrichment of YY1 binding on the promoter of genes involved in protein translation and eventual YY1 displacement after AG636 treatment could strengthen the data.
(6) The CRISPR screen is confirming the importance of YY1 in mediating the effects of AG636 treatment since components of INO80 remodeling complex were identified as AG636 specific genes. Nonetheless, authors do not follow up on the effect on protein translation after INO80 complex silencing. The authors focus on CDK5, another target identified as a top depleted sgRNA in response to AG636. The authors should provide additional data on CDK5 KO cells: can CDK5 KO be rescued by PUGNAc treatment? Is CDK5 synergizing with AG636 by acting on the translation step or are they acting on different pathways? What are the CDK5 targets?
(7) Finally, the title of the manuscript is partially misleading and not really supported by the data presented. Either include experiment directly addressing the effect of DHODH inhibition on protein translation (e.g. using OP-Puro) or revise the title of the manuscript.

Referee #2 (Comments on Novelty/Model System for Author):

Adequate use of mouse system and in vitro culture for pre-clinical testing.

Referee #2 (Remarks for Author):

The authors present results showing that AG636, an inhibitor of the pyrimidine synthesis enzyme DHODH, inhibits AML growth in mice and in vitro. They explore the mechanism of action of AG636 and show that it acts on protein synthesis, possibly via regulation of YY1. Probably the most important contribution is the authors' results from a screen to find genes whose loss potentiates or rescues the effect of DHODH inhibition. The authors uncover cdk5 and other genes as new modifiers of the effects of DHODH inhibitors. This finding may inform selection of AML patients who would benefit from DHODH inhibition. It also opens new directions for understanding the mechanisms of DHODH activity.
specific comments:
1. The effects of AG636 on inhibiting leukemia development and extending mouse survival are robust and striking.
2. I appreciate the authors' point that the effects of AG636 on hematopoietic cells are not as dramatic as its effects on leukemia cells. However, their results show some effects on normal hematopoiesis that could lead to therapy-related toxicities. For example, effects on hematopoietic stem cells are difficult to interpret without assessing the impact of the drug during hematopoietic injury and reconstitution. Given the reduction in HSC and myeloid progenitor frequency it is possible that AG636 impairs myelopoiesis. While I agree with the conclusion that a good therapeutic window for AG636 exists in the mouse, the impact on normal hematopoiesis isn't negligible and should be discussed a bit more in the text. Alternatively, if the authors deem it appropriate, reconstitution experiments could be done to rule out a significant impact on HSCs or myelopoiesis. Note that calculations of HSC, LSK and myeloid progenitor frequencies are complicated by the upregulation of the surface marker Sca-1 in treated animals.

3. A main conclusion is that AG636 downregulates translation. Can the authors directly measure the impact of the drug on the rate of translation using an established method? E.g. by measuring the rate of AHA or HPG methionine analogue or OP-Puromycin incorporation into proteins.

4. A main conclusion is that DHODH inhibition blocks translation via downregulation of YY1. That conclusion is supported by gene expression analysis and by findings that YY1 inhibition reduces AML cell growth. The authors should provide some evidence that YY1 inhibition mediates the effects of AG636 on translation or AML growth. E.g. does PUGNAc, which rescues YY1 levels after AG636, also rescue AML growth or protein synthesis rate after AG636?

Referee #3 (Remarks for Author):

The striking effect of dihydroorotate dehydrogenase inhibition on AML development was serendipitously discovered already a few years ago by Sykes and colleagues in an unbiased small molecule screen. This was done essentially in the same leukemia

model as it is used in this manuscript.

It is nice that the authors can confirm the earlier results in figures 1 and 2, yet, this is not new. The merit of the current work rather lies in the attempt to characterize the underlying molecular mechanism.

Unfortunately, the strategy to pick a single feature of a very pleiotropic biological response as likely explanation for the complex phenotype has a major drawback. More likely than not this will not do justice to the real nature of the multi-layered phenomenon.

To pick the extremely pleiotropic transcription factor YY1 solely based on motif discovery and to attribute a reduction of ribosome biogenesis to a relative reduction of O-GlcNAc stabilized YY1 seems to be premature at this stage.

Specific points:

1. Figure 3: Rather that the drop of B-cells, the severe reduction of CMP and MEP is more concerning. What happens to myeloid numbers in healthy mice after longer treatment? Do you see myelosuppression? This seems to be more important than the discussion about lymphocytes.

2. Can you complement the reduction in transcription of ribosomal components by addition of uridine?

3. Although shortly mentioned, the role of the ATF4 cellular alarm system is neglected. Can induction of ATF4 (e.g. by conditional over-expression) cause similar effects in AML cells? What about ATF4(phospho) levels after treatment with AG636. YY1 is involved in transcription of a multitude of genes. Maybe any severe perturbation of the cellular transcriptome will cause a reduction of ribosomal biogenesis. Is there any evidence (e.g. ChIP) data that these genes are really under direct control of YY1?

4. It is known that Myc is stabilized by O-GlcNAc. (Already mentioned in the original paper.) In contrast to YY1, it is well established that Myc controls transcription of ribosomal genes. How does Myc half-life/amount change after AG636 treatment? Likewise, it has been recently demonstrated that DOT1L, a histone methyltransferase particularly essential for MLL-AF9 induced leukemia is regulated by O-GlcNAc. What happens to Dot1L and/or H3K79 methylation in AG636 treated cells? In the light of these facts, it seems extremely unlikely that YY1 would be the sole decisive factor.

5. The dropout screen lacks any control for specificity. The design does not allow to discriminate for hits that sensitize AML cells towards any detrimental treatment or if this is anything specific for pyrimidine starvation. Quite logically, hits like a general transcriptional complex (Ino80) that is important for not only transcription but also for DNA repair and replication and a kinase (CDK5) that regulates cell survival and apoptosis may be expected. Yet, they are not that revealing with respect to the specific events triggered by dihydroorotate-dehydrogenase inhibition.

During our pre-decision cross-commenting process, Referee #2 made additional comments, which I have included below.

I largely agree with the other reviewers' comments. A minimum set of experiments I'd like to see are: a) measurement of translation rates after DHODH inhibition - it's sufficient to do this in culture although it can also be done in vivo by administering op-puro to mice. Important to do this early on after inhibition before general anti-growth effects kick in that might shut off translation a a secondary effect. 2) Assessment of the effect of DHODH inhibition on myelopoiesis recovery in vivo after a myeloablative insult such as 5-FU, cyclophosphamide or irradiation. It is particularly important to test this if the idea is to give this drug to AML patients in combination with chemotherapy, 3) Rescue experiments of the phenotypes of DHODH inhibition with PUGNAc or with some other way to restore YY1 levels. These can easily be done with current tools within 2-3 months. I agree with ref 3 re specificity of the sensitization screen, but its hard to see how this can be addressed without a lot more work to mechanistically tie cdk5 or other hits to DHODH inhibition (which in my view is beyond the scope of the current paper). The authors have already done a bit to address this by showing that cdk5 loss does not sensitize to AraC treatment and by showing that INO80 factors that are generally required in AML don't preferentially sensitize to DHODH inhibition. I would be content with the authors discussing the specificity issues of the screen in the discussion section.

Dear Dr Hou,
We thank the referees for their constructive feedback which has helped us to further improve our manuscript. Please find below a point-by-point response. In summary, the major new findings are:

(1) Myelopoiesis is moderately suppressed by AG636, but recovers after treatment is withdrawn
(2) AG636 inhibits nascent protein synthesis as measured by the AHA incorporation assay
(3) YY1 is physically present at the promoters of translation genes and is displaced after AG636 treatment
(4) CDK5 knockout cells have divergent transcription responses to DHODH inhibition

The revised manuscript has been formatted to comply with the journal guidelines and the changes are highlighted. Thank you again for your consideration and we look forward to hearing from you soon.

Best wishes,

Lev Kats, PhD
Cancer Biology and Therapeutics Program
Peter MacCallum Cancer Centre
Web: https://www.petermac.org/research/labs/lev-kats
Phone: +61-3-8559-5834
E-mail: lev.kats@petermac.org

Referee #1 (Remarks for Author):
The manuscript entitled "Inhibition of pyrimidine biosynthesis targets protein translation in AML" by Joan So and colleagues point to the possibility of limiting protein translation by inhibiting DHODH, and thus propose it as a potential new therapeutic target for the treatment of AML. The authors first demonstrated the effects of DHODH inhibition in leukemia progression in mouse models for MLL rearranged leukemia. They further validate the findings using models of non MLL rearranged leukemia. In addition, they tested the effect of DHODH inhibition in normal hematopoiesis. To understand the molecular mechanism of the observed therapeutic effects, they performed transcriptomic analyses and identified genes involved in protein translation that were downregulated after inhibition of DHODH, including YY1, a transcriptional regulator of protein translation. By performing a CRISPR targeted screen focused on epigenetic modifiers, they identified gene candidates which rescue DHODH inhibition as well as ones showing synthetic lethality. The paper is well written and the experimental design as well as the data are very well presented. However, the paper is mainly focusing on the analysis of the transcriptional effects of DHODH inhibition rather than deeply characterizing protein translation as claimed in the title. Additional experimental data should be provided to directly (rather than indirectly by transcriptional analysis) examine the connection between DHODH inhibition and protein translation.
Response:
We thank the reviewer for this comment and note that referee #2 also requested these data. Using the L-azidohomoalanine (AHA) incorporation assay we found that DHODH inhibition reduced nascent protein synthesis in MN and MOLM13 cells by 52-84% within 24 hours of treatment. These new findings are included in Fig 4I and Appendix Fig S4B and are concordant with our observations that AG636 causes transcriptional downregulation of translation genes and reduced polysomes in AML cells.

To some degree, the novelty is affected as the role of DHODH as a potential therapeutic AML target was already reported (Sykes *et al*., Cell 2016; Christian *et al*., Leukemia 2019; Gaidano *et al*., Cancers 2021). Nevertheless, this is an interesting, important and technically well performed study.
Response:
We agree with the reviewer that earlier studies have demonstrated the potential of DHODH as a drug target in AML. However, we believe that there are two aspects of our study that significantly advance the field:
(a) Most studies investigating DHODHi to date have largely focused on AML cell lines cultured *in vitro* or xenografted into immune-compromised mice. Although tractable, cell lines do not adequately model many features of human AML and in particular AML LSCs. Our use of syngeneic models and comprehensive analysis of the effects of AG636 on normal hematopoiesis provides important pre-clinical evidence of both efficacy and the existence of a therapeutic window for DHODHi that is a significant advance on previous work
(b) We are the first to identify a functional role for the YY1/INO80 axis in the response of LSCs to pyrimidine starvation and the synthetic lethal relationship between CDK5 and DHODH

Prior to publication, the following issues should be addressed with the goal of further improving the manuscript:
(1) In extended Fig. 1 it is shown the overall survival of secondary recipients transplanted with either naïve (M30) or leukemia cells derived from primary mice showing leukemia relapse (M15). While M15 show high sensitivity to DHODH inhibition excluding the possibility of acquired resistance, DHODH inhibitor treated M30 with did not show prolonged survival as shown in Fig. 1. Please comment on this and if required, repeat the experiment by including more secondary recipients obtained from naïve primary leukemia mouse models.
Response:
To clarify, in the experiment in Fig 1C mice were dosed with AG636 for four weeks, whereas in the experiment in Fig EV1E (previously Extended Data Fig 1d) mice were dosed with AG636 for two weeks. We used a reduced two-week regimen because we reasoned that it would be more likely to uncover partial resistance in the M15 relapsed leukemia (compared with the full four-week treatment that may overcome partial resistance). We believe that the following interpretation of the data is appropriate:
(a) By comparing the response of M15 and M30 leukemias in the secondary transplant, we conclude that leukemic cells that persist during four weeks of therapy retain a high degree of AG636 sensitivity

(b) The observation that two weeks of treatment in the secondary transplant for the M30 leukemia is less effective than four weeks of treatment in the primary transplant suggests that the extended treatment duration may provide additional benefit. We have added additional text in the results section (lines 123-125)

(2) Fig. 3 shows the effect of DHODH inhibition on normal hematopoiesis. Data are shown after 1 or 4 days of treatment. In contrast, the treatment of leukemic mice followed a treatment scheme of 4 cycles of 5 days each. It would be important to assess the effect on normal hematopoiesis using the same treatment scheme. In addition, the authors should perform a time course experiment in order to assess the time needed for the normal hematopoietic system to recover from this treatment.
Response:
We agree with the reviewer that this is an important question. To clarify, in our initial analysis we treated non-tumor bearing mice for one or four days, the same treatment regimen that was used in the cross-sectional analyses of tumor bearing mice (Figs 1D-I and 2). Following the reviewer's suggestion, we performed an additional experiment treating non-tumor bearing mice with AG636 for four cycles (100mg/kg b.i.d. on days 1-5 of a 7-day cycle) and then allowing mice to recover. We found that AG636 treatment caused progressive lymphocytopenia, but that both lymphoid and myeloid populations in the peripheral blood recovered to baseline levels within 2-4 weeks of treatment withdrawal. Analysis of bone marrow populations after four weeks found no differences between mice that had received AG636 treatment and vehicle treated controls. The new data are included in Figs 3B-H and Appendix Fig S3B-C (portions of the data presented in the original manuscript have been moved to Appendix Fig S3). Altogether, these data confirm the selective vulnerability of AML cells to DHODH inhibition.

(3) Fig. 4 addresses the transcriptional changes induced by DHODH inhibition. Transcriptomic data are generated in all the leukemia mouse models. It would be highly interesting to determine the common DEGs between all these leukemic mouse models treated similarly.
Response:
We thank the reviewer for this suggestion. We compared the impact of DHODH inhibition in all 3 models both at the level of individual genes and biological pathways. This analysis has been added to Figure EV2A-B. Overall, we find significant overlap in DEGs and pathways between LSCs isolated from the different models.

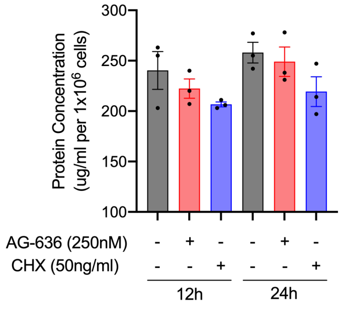

Rebuttal Figure 1. MN cells were treated with AG636 or cycloheximide for the indicated amount of time and protein concentration was quantified using the BCA assay. Dara are represented as mean ± s.e.m.

(4) Differentially expressed genes important for protein translation are validated in vitro, as well as in AML cell lines and by ribosome profiling. Based on the changes shown in the ribosome profiling, one would expect a massive block in protein translation. Is the amount of total proteins/cell different between AG636 and vehicle treated cells?
Response: As detailed above, using the AHA assay we found that AG636 treatment reduced nascent protein translation in MN and MOLM13 cells within 24 hours of AG636 treatment. For completeness, we also quantified total protein/cell, but found that both AG636 and cycloheximide only had a marginal effect on protein concentration as measured by the BCA assay. We have included these data as Rebuttal Fig 1.

(5) The authors identified YY1 as the transcription factor candidate mediating the effect of AG636 and suggest an involvement of O-GlcNAc modifications. To confirm these data, the authors analyzed expression levels of genes involved in protein translation after YY1 KD. Among the many candidates, why was YY1, which is only marginally affected by AG636, was chosen to be followed up?
Response:
There were 4 reasons that led us to explore the role of YY1 in the AG636 transcriptional response:
(a) We found that YY1 motifs were highly enriched in open chromatin associated with translation genes in MN cells, suggesting that YY1 was physically present at those chromatin regions in AML cells

(b) Members of the INO80 complex, which functions with YY1 to activate transcription, were identified as modulating the sensitivity of AML cells to AG636 in our CRISPR screen (as also pointed out by the reviewer)
(c) YY1 has been implicated in positively regulating transcription of protein translation genes and downregulation of the Reactome translation gene set has been reported as a prominent signature upon YY1 knockout (Kleiman *et al*, 2016; Varum *et al*, 2019; Zurkirchen *et al*, 2019).
(d) YY1 stability is known to be regulated by O-GlcNAc (Hiromura *et al*, 2003).
(e) We searched our RNAseq data for transcription factors that were downregulated by AG636 treatment (at the mRNA level) but could not identify any candidates with a known role in regulating translation genes.

Our additional analysis of ENCODE data and ChIP-qPCR confirms that YY1 is indeed enriched at promoters of translation genes as well as other AG636 downregulated genes (Figs 5C and D and EV3B and C in the revised manuscript). Altogether our data support a major role for YY1 in the AG636 transcriptional response, however, we agree that other factors also likely contribute. Following the suggestion of reviewer 3 we tested the impact of DHODH inhibition of expression of MYC, DOT1L and ATF4. MYC and DOT1L are downregulated by AG636 whereas ATF4 is upregulated (Fig EV3A in the revised manuscript, see also discussion below). We also modified the discussion section to better reflect the multifaceted nature of the transcriptional and cellular response to pyrimidine starvation (lines 488-493).

The specific effect of YY1 in the control of protein translation gene expression is not convincing. Transcriptomic data after YY1 KD and comparison to AG636 treated cells should be provided.

Response:

Unfortunately, we have found it technically challenging to measure the impact of YY1 on gene expression in AML cells because YY1 is highly essential in this context (Fig EV4C and Appendix Fig S5G). Transduction of YY1 targeting sgRNAs into MN cells resulted in low transduction efficiency and rapid dropout. We attempted to sort cells using the Crimson (Crim) reporter co-expressed from the sgRNA vector at various timepoints shortly after transduction, but these experiments lack high temporal resolution due to the asynchronous nature of gene inactivation within the Crim$^+$ pool. Hence, it is difficult to capture cells after significant loss of YY1 protein but prior to cell cycle arrest and/or apoptosis.

To address the reviewer's question in another way, we analyzed publicly available data from YY1 knockout murine pro-B cells generated using a conditional YY1 knockout allele (Kleiman *et al*, 2016). YY1 knockout pro-B cells are viable, providing an opportunity to analyze the impact of YY1 deletion on gene expression. Gene set enrichment analysis revealed that translation genes are indeed downregulated in YY1 knockout cells (Fig 5H in the revised manuscript). Moreover, in an independent dataset in human melanoma cells (Varum *et al*, 2019) siRNA mediated YY1 depletion similarly resulted in downregulation of protein translation genes (Fig 5H in the revised manuscript). Combined with our other observations, including that YY1 is enriched at the promoters of translation genes (see below) and that disruption of the INO80 complex sensitizes cells to AG636, these analyses support a model whereby the downregulation of YY1 plays a major role in transcriptional response of AML cells to AG636 treatment.

Furthermore, YY1 ChIP data proving the enrichment of YY1 binding on the promoter of genes involved in protein translation and eventual YY1 displacement after AG636 treatment could strengthen the data.

Response:

We thank the reviewer for this important suggestion. We first analyzed publicly available ChIPseq data in the ENCODE3 database (1264 ChIPseq datasets for 338 chromatin associated factors across 130 cell lines). This unbiased analysis revealed that:

(a) YY1 is physically present at most translation genes in the majority of the 16 cell lines for which YY1 ChIPseq data is available in ENCODE.
(b) YY1 is amongst the most enriched transcription factors present at translation genes in the ENCODE database.

Using ChIP-qPCR, we also confirmed that YY1 is enriched at the promoters of a subset of translation genes in MN cells and is displaced after AG636 treatment. These data have been included in Figs 5C and D and EV3B and C in the revised manuscript.

Peter MacCallum Cancer Centre
305 Grattan Street
Melbourne Victoria
3000 Australia
ABN 42 100 504 883
petermac.org

(6) The CRISPR screen is confirming the importance of YY1 in mediating the effects of AG636 treatment since components of INO80 remodeling complex were identified as AG636 specific genes. Nonetheless, authors do not follow up on the effect on protein translation after INO80 complex silencing. The authors focus on CDK5, another target identified as a top depleted sgRNA in response to AG636. The authors should provide additional data on CDK5 KO cells: can CDK5 KO be rescued by PUGNAc treatment? Is CDK5 synergizing with AG636 by acting on the translation step or are they acting on different pathways? What are the CDK5 targets?
Response:
We tested the impact of CDK5 deletion on gene expression at baseline and in response to AG6363 treatment by RNAseq. Downregulation of translation genes and nascent protein synthesis in response to AG636 was comparable in CDK5 knockout and control cells. However, we found that DHODH inhibition induced a different transcriptional response in CDK5 knockout cells including in genes involved in the electron transport chain, mitochondrial gene expression, DNA replication and mRNA catabolism. These new data have been included in Figs 7C-E in the revised manuscript and EV5C-D. We also found that co-treatment with PUGNAc did not rescue the proliferative disadvantage of CDK5 knockout cells in the presence of AG636 (Rebuttal Fig 2).

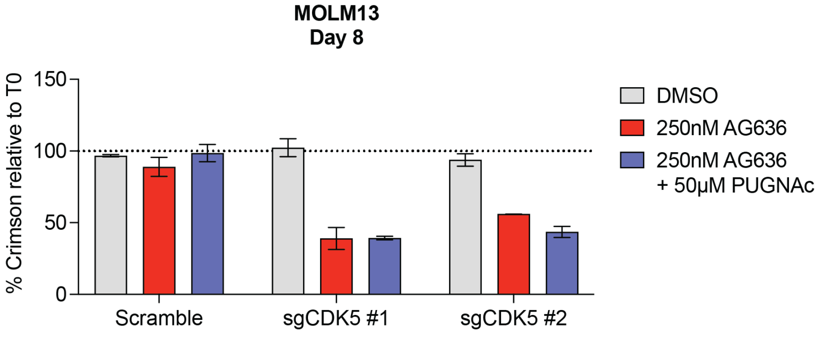

Rebuttal Figure 2. Proliferative competition assays in AML cells transduced with CDK5 or control sgRNAs and cultured in AG636, AG636 + PUGNAc or DMSO.

(7) Finally, the title of the manuscript is partially misleading and not really supported by the data presented. Either include experiment directly addressing the effect of DHODH inhibition on protein translation (e.g. using OP-Puro) or revise the title of the manuscript.
Response:
As detailed above, we now present direct experimental evidence that DHODH inhibition results in downregulation of protein synthesis in AML cells. Hence, we believe that the title is appropriate.

Referee #2 (Comments on Novelty/Model System for Author):
Adequate use of mouse system and in vitro culture for pre-clinical testing.

Referee #2 (Remarks for Author):
The authors present results showing that AG636, an inhibitor of the pyrimidine synthesis enzyme DHODH, inhibits AML growth in mice and in vitro. They explore the mechanism of action of AG636 and show that it acts on protein synthesis, possibly via regulation of YY1. Probably the most important contribution is the authors' results from a screen to find genes whose loss potentiates or rescues the effect of DHODH inhibition. The authors uncover cdk5 and other genes as new modifiers of the effects of DHODH inhibitors. This finding may inform selection of AML patients who would benefit from DHODH inhibition. It also opens new directions for understanding the mechanisms of DHODH activity.

Specific comments:
1. The effects of AG636 on inhibiting leukemia development and extending mouse survival are robust and striking.
Response:
We agree with the reviewer that AG636 has very potent activity in the pre-clinical models that we tested, especially in the MN model. We hope that our manuscript will promote further pre-clinical and clinical development of DHODH inhibitors for AML.

Peter MacCallum Cancer Centre
305 Grattan Street
Melbourne Victoria
3000 Australia
ABN 42 100 504 883
petermac.org

Peter Mac
Peter MacCallum Cancer Centre
Victoria Australia

2. I appreciate the authors' point that the effects of AG636 on hematopoietic cells are not as dramatic as its effects on leukemia cells. However, their results show some effects on normal hematopoiesis that could lead to therapy-related toxicities. For example, effects on hematopoietic stem cells are difficult to interpret without assessing the impact of the drug during hematopoietic injury and reconstitution. Given the reduction in HSC and myeloid progenitor frequency it is possible that AG636 impairs myelopoiesis. While I agree with the conclusion that a good therapeutic window for AG636 exists in the mouse, the impact on normal hematopoiesis isn't negligible and should be discussed a bit more in the text. Alternatively, if the authors deem it appropriate, reconstitution experiments could be done to rule out a significant impact on HSCs or myelopoiesis. Note that calculations of HSC, LSK and myeloid progenitor frequencies are complicated by the upregulation of the surface marker Sca-1 in treated animals.

Response:

We thank the reviewer for this suggestion. We treated mice with AG636 for four cycles and then followed them for an additional four weeks after treatment. We did indeed observe that AG636 impairs myelopoiesis, although the effects were reversed after drug was discontinued. The new data are included in Fig 3 and Appendix Fig S3 in the revised manuscript (portions of the data presented in the original manuscript have been moved to Appendix Fig S3). We also analyzed recipient-derived myeloid cells in mice that had been engrafted with MN cells and survived following treatment with AG636 or doxycycline. These mice had received 3.5Gy whole-body irradiation as a conditioning regimen prior to tumor engraftment. We found no differences in myeloid recovery between AG636 and doxycycline treated groups, suggesting that AG636 is tolerable in the context of other cytotoxic therapies (Fig EV1C in the revised manuscript). The discussion of our findings of the impact of DHODHi on normal hematopoiesis has been edited as suggested (lines 465-477). We also inserted a statement regarding the potential confounding effects of AG636-mediated Sca1 upregulation of quantification of stem and progenitor populations (lines 186-187).

3. A main conclusion is that AG636 downregulates translation. Can the authors directly measure the impact of the drug on the rate of translation using an established method? E.g. by measuring the rate of AHA or HPG methionine analogue or OP-Puromycin incorporation into proteins.

Response:

Using the AHA incorporation assay we found that DHODH inhibition reduced nascent protein synthesis in MN and MOLM13 cells and that this phenotype could be reversed by uridine supplementation. The new data are included in Figs 4I, EV3F and Appendix Fig S4B.

4. A main conclusion is that DHODH inhibition blocks translation via downregulation of YY1. That conclusion is supported by gene expression analysis and by findings that YY1 inhibition reduces AML cell growth. The authors should provide some evidence that YY1 inhibition mediates the effects of AG636 on translation or AML growth. E.g. does PUGNAc, which rescues YY1 levels after AG636, also rescue AML growth or protein synthesis rate after AG636?

Response:

We provide the following additional evidence in the revised manuscript to support a major role for YY1 in the AG636 phenotype:

(a) ChIP-qPCR analyses demonstrate that YY1 is physically present at the promoters of translation genes and is depleted following AG636 treatment in AML cells (Fig 5D). Publicly available ChIPseq data demonstrate that YY1 is among the most enriched transcription factors at translation genes and that YY1 is also enriched at other AG636 downregulated genes in a wide variety of cell lines (Fig 5C and EV3A and B). These new findings are in agreement with our ATACseq analysis presented in the original submission that identified enrichment of YY1 motifs in regions of open chromatin associated with translation genes.

(b) Transcriptional downregulation of translation genes by AG636 treatment is partially rescued by PUGNAc in MOLM13 cells. The observation that PUGNAc does not rescue downregulation of protein translation by AG636 suggests that other factors likely contribute to this phenotype. It also likely points to off target effects of PUGNAc, which inhibits a variety of N-acetylhexosaminidases.

We have included additional discussion in the text regarding the multifaceted nature of DHODH inhibition (lines 488-493). See also discussion for reviewers 1 and 3 regarding the impact of AG636 on MYC, DOT1L and ATF4.

Peter MacCallum Cancer Centre
305 Grattan Street
Melbourne Victoria
3000 Australia
ABN 42 100 504 883
petermac.org

Referee #3 (Remarks for Author):
The striking effect of dihydroorotate dehydrogenase inhibition on AML development was serendipitously discovered already a few years ago by Sykes and colleagues in an unbiased small molecule screen. This was done essentially in the same leukemia model as it is used in this manuscript. It is nice that the authors can confirm the earlier results in figures 1 and 2, yet, this is not new. The merit of the current work rather lies in the attempt to characterize the underlying molecular mechanism. Unfortunately, the strategy to pick a single feature of a very pleiotropic biological response as likely explanation for the complex phenotype has a major drawback. More likely than not this will not do justice to the real nature of the multi-layered phenomenon. To pick the extremely pleiotropic transcription factor YY1 solely based on motif discovery and to attribute a reduction of ribosome biogenesis to a relative reduction of O-GlcNAc stabilized YY1 seems to be premature at this stage.
Response:
In the revised manuscript we present additional data to support a major role for YY1 in the transcriptional response to AG636. YY1 has not previously been implicated as a regulator downstream of pyrimidine starvation, hence we believe that our findings make an important and novel contribution to the field. However, we agree with the reviewer that the effect of DHODH inhibition is likely to be multifaceted and we have modified the discussion section accordingly (see also above and below regarding MYC, DOT1L, ATF4/ISR).

Specific points:
1. Figure 3: Rather that the drop of B-cells, the severe reduction of CMP and MEP is more concerning. What happens to myeloid numbers in healthy mice after longer treatment? Do you see myelosuppression? This seems to be more important than the discussion about lymphocytes.
Response:
We thank the reviewer for this suggestion which was also noted by others. Please see response to reviewer 2 point 2 above.

2. Can you complement the reduction in transcription of ribosomal components by addition of uridine?
Response:
As suggested, we found that exogenous uridine complements downregulation of translation genes, nascent protein synthesis and proliferation *in vitro.* These new data are presented in Figs EV3F and G.

3. Although shortly mentioned, the role of the ATF4 cellular alarm system is neglected. Can induction of ATF4 (e.g. by conditional over-expression) cause similar effects in AML cells? What about ATF4(phospho) levels after treatment with AG636.
Response:
We found that ATF4 protein levels are increased following AG636 treatment (Fig EV3A in the revised manuscript) concordant with activation of the integrated stress response (ISR) as previously reported (Evstafieva *et al*, 2014) and the enrichment of ATF4 motifs observed in our ATACseq analysis (Fig 5A). Notably however, ATF4 motifs were not enriched within the promoter regions of translation genes downregulated by AG636. We also performed gene set enrichment analysis using RNAseq data from MN, RUNX1-RUNX1T1 and I1DN LSCs treated with AG636 and did not observe a significant transcriptional upregulation of ATF4 target genes (Rebuttal Fig 3). Collectively this suggests that ATF4 may contribute to

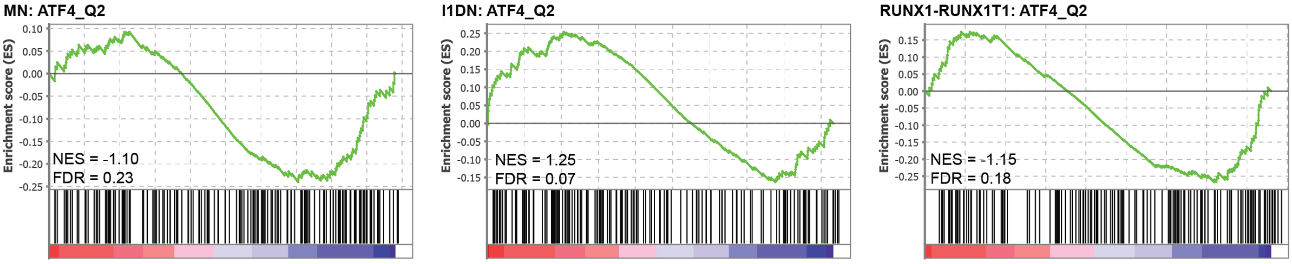

Rebuttal Figure 3. Barcode plots showing variable impact of AG636 on ATF4 target genes in LSCs from the three different AML models.

Patron: The Honourable Linda Dessau, AM – Governor of Victoria

Peter MacCallum Cancer Centre
305 Grattan Street
Melbourne Victoria
3000 Australia
ABN 42 100 504 883
petermac.org

the molecular response to DHODH inhibition but does not play a defining role. We have included additional discussion regarding the potential contribution of ATF4/ISR at line 492-493.

YY1 is involved in transcription of a multitude of genes. Maybe any severe perturbation of the cellular transcriptome will cause a reduction of ribosomal biogenesis. Is there any evidence (e.g. ChIP) data that these genes are really under direct control of YY1?
Response:
We agree with the reviewer that this is an important point. Please see detailed responses to reviewer 1 point 5 and reviewer 2 point 4. Briefly, using ChIP-qPCR we found that YY1 is physically present at the promoters of translation genes and is depleted following AG636 treatment. Furthermore, analysis of publicly available ChIPseq data revealed that YY1 is enriched at translation genes and other AG636 downregulated genes in a wide variety of cell lines. These new findings are included in Figs 5C and D and EV3B and C in the revised manuscript. We also analyzed gene expression data from AML cell lines treated with inhibitors of MEN1 and DOT1L (Dafflon *et al*, 2017) and found that these compounds do not drive transcriptional downregulation of translation genes providing further evidence of the specificity of the AG636 transcriptional response (Rebuttal Fig 4).

Normalized gene count in REACTOME_Translation
GSE63664 (Dafflon et al., 2016)

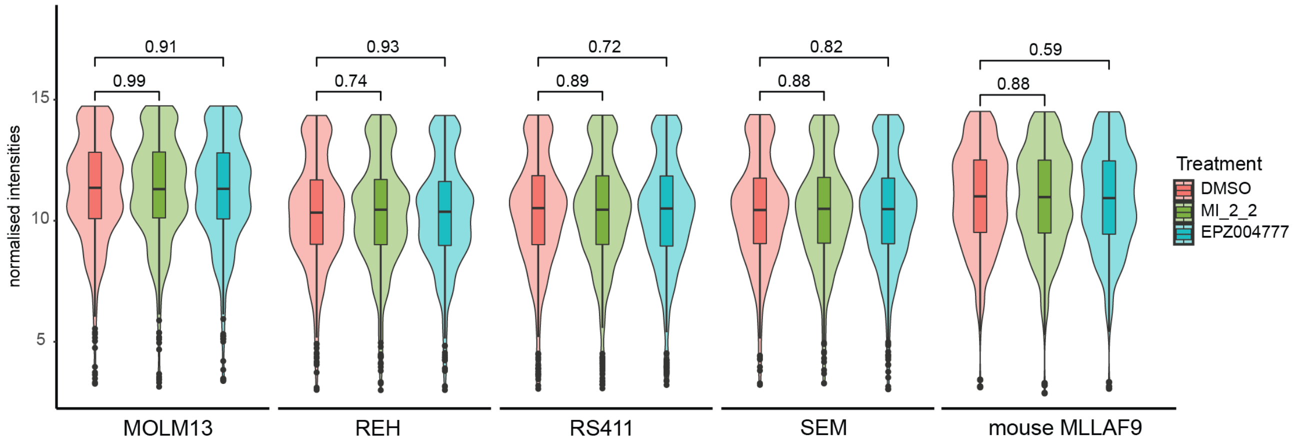

Rebuttal Figure 4. Violin plots showing the normalized counts of the genes in the Reactome_Translation gene set in human and murine AML cells following MI_2_2 (Menin inhibitor) and EPZ004777 (DOT1L inhibitor) treatment.

4. It is known that Myc is stabilized by O-GlcNAc. (Already mentioned in the original paper.) In contrast to YY1, it is well established that Myc controls transcription of ribosomal genes. How does Myc half-life/amount change after AG636 treatment? Likewise, it has been recently demonstrated that DOT1L, a histone methyltransferase particularly essential for MLL-AF9 induced leukemia is regulated by O-GlcNAc. What happens to Dot1L and/or H3K79 methylation in AG636 treated cells? In the light of these facts, it seems extremely unlikely that YY1 would be the sole decisive factor.
Response:
We thank the reviewer for giving us an opportunity to clarify our conclusion. Our model is that YY1 is a major factor driving the AG636 response, though it is not the sole or decisive factor. As discussed in detail above (see response to reviewer 1 point 5) this model is supported by multiple lines of evidence including the finding that YY1 is physically present at AG636 downregulated genes and that components of the YY1 interacting INO80 complex modulate response to DHODH inhibition. As suggested by the reviewer we analyzed expression of MYC and DOT1L and found that both proteins are downregulated by AG636 treatment. These data are presented in EV3A. Overall, we believe that our revised manuscript strikes an appropriate balance between highlighting the previously unknown role of YY1 while also acknowledging the contribution of other pathways in the complex cellular response to DHODH inhibitors (see discussion at lines 488-493).

Peter MacCallum Cancer Centre
305 Grattan Street
Melbourne Victoria
3000 Australia
ABN 42 100 504 883
petermac.org

5. The dropout screen lacks any control for specificity. The design does not allow to discriminate for hits that sensitize AML cells towards any detrimental treatment or if this is anything specific for pyrimidine starvation. Quite logically, hits like a general transcriptional complex (Ino80) that is important for not only transcription but also for DNA repair and replication and a kinase (CDK5) that regulates cell survival and apoptosis may be expected. Yet, they are not that revealing with respect to the specific events triggered by dihydroorotate-dehydrogenase inhibition.

Response:

In the revised manuscript we present evidence that loss of CDK5 does not sensitize AML cells to cytarabine, etoposide, the DOT1L inhibitor SGC0946 or the BRD4 inhibitor JQ1, demonstrating the selective nature of the CDK5 knockout phenotype (Figs 7A and EV5A; Appendix Fig S6A in the revised manuscript). Conversely, many factors known to be essential for proliferation of MN cells (e.g. DOT1L or BRD4) did not score in our screen. Moreover, in >10 CRISPR drug screens performed in our laboratory using a range of compounds (hypomethylating agents, Menin inhibitors, etc) we have not identified CDK5 or INO80 components as sensitizer hits, nor did CDK5 or INO80 components score in published CRISPR screens that we reviewed. As an example, we include a screen that was specifically powered to identify sensitizer hits to the BRD4 inhibitor JQ1 in Rebuttal Fig 5 (Estoppey *et al*, 2021). Taken together, these analyses strongly support the conclusion that our CRISPR screen identified hits with a degree of specificity for DHODH inhibitors in AML.

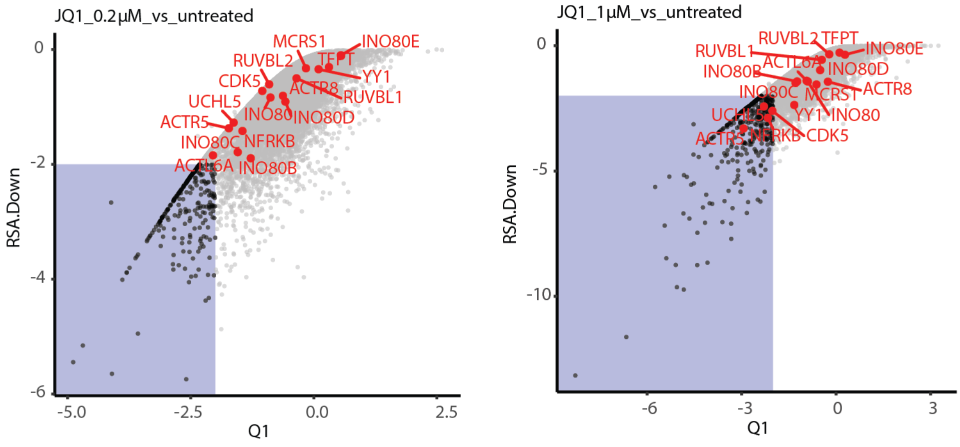

Rebuttal Figure 5. Genome-wide CRISPR-Cas9 screen showing genes that were negatively selected in HCT119 cells treated with JQ1. Authors define sensitization as Q1 < -2 and RSA.down < -2 (Estoppey *et al*, 2021).

During our pre-decision cross-commenting process, Referee #2 made additional comments, which I have included below.

I largely agree with the other reviewers' comments. A minimum set of experiments I'd like to see are:
1) measurement of translation rates after DHODH inhibition - it's sufficient to do this in culture although it can also be done in vivo by administering op-puro to mice. Important to do this early on after inhibition before general anti-growth effects kick in that might shut off translation as a secondary effect.

Response:

As discussed in detail above we present evidence in the revised manuscript that AG636 inhibits nascent protein synthesis as quantified using the AHA incorporation assay.

2) Assessment of the effect of DHODH inhibition on myelopoiesis recovery in vivo after a myeloablative insult such as 5-FU, cyclophosphamide or irradiation. It is particularly important to test this if the idea is to give this drug to AML patients in combination with chemotherapy

Response:

We quantified the impact of DHODH inhibition on myelopoiesis recovery in non-tumor bearing mice. Due to time constraints (as a new animal ethics application would have been required), it was not feasible to perform combination studies with AG636 and 5-FU or cyclophosphamide. However, we analyzed myelopoiesis recovery in tumor-bearing mice that had received 3.5Gy whole-body irradiation as a conditioning regimen. This analysis revealed no differences between mice that were treated with AG636 or doxycycline.

3) Rescue experiments of the phenotypes of DHODH inhibition with PUGNAc or with some other way to restore YY1 levels. These can easily be done with current tools within 2-3 months. I agree with ref 3 re specificity of the sensitization screen, but its hard to see how this can be addressed without a lot more work to mechanistically tie cdk5 or other hits to DHODH inhibition (which in my view is beyond the scope of the current paper). The authors have already done a bit to address this by showing that cdk5 loss does not sensitize to AraC treatment and by showing that INO80 factors that are generally required in AML don't preferentially sensitize to DHODH inhibition. I would be content with the authors discussing the specificity issues of the screen in the discussion section.

Response:

We performed rescue experiments using PUGNAc and uridine as suggested by the reviewers. We also provide additional evidence of the "specificity" of the CDK5 knockout phenotype with respect to DHODH inhibition and performed gene expression analysis that characterized divergent transcriptional responses to AG636 in CDK5 knockout AML cells.

Rebuttal References

Dafflon C, Craig VJ, Méreau H, Gräsel J, Schacher Engstler B, Hoffman G, Nigsch F, Gaulis S, Barys L, Ito M, Aguadé-Gorgorió J, Bornhauser B, Bourquin J-P, Proske A, Stork-Fux C, Murakami M, Sellers WR, Hofmann F, Schwaller J & Tiedt R (2017) Complementary activities of DOT1L and Menin inhibitors in MLL-rearranged leukemia. *Leukemia* **31:** 1269–1277

Estoppey D, Schutzius G, Kolter C, Salathe A, Wunderlin T, Meyer A, Nigsch F, Bouwmeester T, Hoepfner D & Kirkland S (2021) Genome-wide CRISPR-Cas9 screens identify mechanisms of BET bromodomain inhibitor sensitivity. *ISCIENCE* **24:** 103323

Evstafieva AG, Garaeva AA, Khutornenko AA, Klepikova AV, Logacheva MD, Penin AA, Novakovsky GE, Kovaleva IE & Chumakov PM (2014) A sustained deficiency of mitochondrial respiratory complex III induces an apoptotic cell death through the p53-mediated inhibition of pro-survival activities of the activating transcription factor 4. *Cell Death and Dis* **5:** e1511–e1511

Hiromura M, Choi CH, Sabourin NA, Jones H, Bachvarov D & Usheva A (2003) YY1 Is Regulated by O-Linked N-Acetylglucosaminylation (O-GlcNAcylation). *J Biol Chem* **278:** 14046–14052

Kleiman E, Jia H, Loguercio S, Su AI & Feeney AJ (2016) YY1 plays an essential role at all stages of B-cell differentiation. *Proc Natl Acad Sci USA* **113:** E3911–20

Varum S, Baggiolini A, Zurkirchen L, Atak ZK, Cantù C, Marzorati E, Bossart R, Wouters J, Häusel J, Tuncer E, Zingg D, Veen D, John N, Balz M, Levesque MP, Basler K, Aerts S, Zamboni N, Dummer R & Sommer L (2019) Yin Yang 1 Orchestrates a Metabolic Program Required for Both Neural Crest Development and Melanoma Formation. *Cell Stem Cell* **24:** 637–653.e9

Zurkirchen L, Varum S, Giger S, Klug A, Häusel J, Bossart R, Zemke M, Cantù C, Atak ZK, Zamboni N, Basler K & Sommer L (2019) Yin Yang 1 sustains biosynthetic demands during brain development in a stage-specific manner. *Nat Commun* **10:** 2192–16

Thank you for the submission of your revised manuscript to EMBO Molecular Medicine. We have now received the enclosed report from the three referees who agreed to re-assess it. As you will see, the referees are now supportive, and I am pleased to inform you that we will be able to accept your manuscript pending the following amendments:

1. Please address Referee #2's comments #1 and #3.

On a more editorial level.

***** Reviewer's comments *****

Referee #1 (Remarks for Author):

So and colleagues thoroughly addressed all the points/comments raised by the three reviewers performing new experiments as well as analyzing publicly available datasets. In summary they further demonstrated 1. the effect the transitory effect of DHODH inhibition on normal hematopoiesis, 2. the reduction of protein synthesis caused by AG636, 3. YY1 chromatin occupancy of genes involved in translation protein synthesis after AG636 treatment, 4. Transcriptional differences between CDK5 KO and DHODH inhibition.

Referee #2 (Comments on Novelty/Model System for Author):

n/a

Referee #2 (Remarks for Author):

The authors have addressed my concerns. Some suggestions are below:

1. The myelosuppressive effects of the drug appear significant. It is encouraging that these effects are quickly reversed after treatment as this suggests that HSCs and immature progenitors are unaffected. Nevertheless, the myelosuppression will be a concern in clinical trials, and it is appropriate that the authors emphasize this effect so that clinical development is informed by it. I would avoid characterizing the effects on normal hematopoiesis as 'minor' as the authors currently do in the discussion. It will be interesting in future research to understand why myeloid progenitors are so sensitive to inhibition of pyrimidine synthesis. Perhaps this is simply a consequence of their high proliferation rate but perhaps there are other reasons as well, e.g. a low pyrimidine salvage capacity which is then inherited by myeloid leukemia cells.
2. What is the difference between appendix figures and EV figures? All these data should be shown in the paper I think.
3. There is a mistake in EV1c- the number of neutrophils in blood is off by some orders of magnitude. Please fix.

Referee #3 (Remarks for Author):

The authors have robustly answered to most of the referees comments. As there was quite a good consensus between referees about the most crucial aspects and all of those have been answered satisfactorily I'd recommend publication at this point.

Referee #2 comments

1. The myelosuppressive effects of the drug appear significant. It is encouraging that these effects are quickly reversed after treatment as this suggests that HSCs and immature progenitors are unaffected. Nevertheless, the myelosuppression will be a concern in clinical trials, and it is appropriate that the authors emphasize this effect so that clinical development is informed by it. I would avoid characterizing the effects on normal hematopoiesis as 'minor' as the authors currently do in the discussion. It will be interesting in future research to understand why myeloid progenitors are so sensitive to inhibition of pyrimidine synthesis. Perhaps this is simply a consequence of their high proliferation rate but perhaps there are other reasons as well, e.g. a low pyrimidine salvage capacity which is then inherited by myeloid leukemia cells.

Response:

We agree that the dependence on DHODH activity does indeed appear to be an intrinsic feature of myeloid progenitors and that the effects of DHODH inhibition on normal myeloid development will need to be managed carefully in clinical trials. To avoid understating the potentially significant impact of AG636 treatment on normal hematopoiesis we have amended the discussion and removed the description “minor” as suggested (see line 462-472 in the revised manuscript).

3. There is a mistake in EV1c- the number of neutrophils in blood is off by some orders of magnitude. Please fix.

We apologize for this error, which appears to have been introduced during PDF conversion. The correct units on the graph are CD45.1^{+}Ly6G^{+} cells per µL blood (not per L blood as appeared in the PDF). This has been corrected in the .eps file.

We are pleased to inform you that your manuscript is accepted for publication and is now being sent to our publisher to be included in the next available issue of EMBO Molecular Medicine.

EMBO Press Author Checklist

Corresponding Author Name: Dr. Lev Kats	
Journal Submitted to: EMBO Molecular Medicine	
Manuscript Number: EMM-2021-15203	

USEFUL LINKS FOR COMPLETING THIS FORM
The EMBO Journal - Author Guidelines
EMBO Reports - Author Guidelines
Molecular Systems Biology - Author Guidelines
EMBO Molecular Medicine - Author Guidelines

Reporting Checklist for Life Science Articles (updated January 2022)
This checklist is adapted from Materials Design Analysis Reporting (MDAR) Checklist for Authors. MDAR establishes a minimum set of requirements in transparent reporting in the life sciences (see Statement of Task: 10.31222/osf.io/9sm4x). Please follow the journal's guidelines in preparing your manuscript.
Please note that a copy of this checklist will be published alongside your article.

Abridged guidelines for figures
1. Data
The data shown in figures should satisfy the following conditions:
- the data were obtained and processed according to the field's best practice and are presented to reflect the results of the experiments in an accurate and unbiased manner.
- ideally, figure panels should include only measurements that are directly comparable to each other and obtained with the same assay.
- plots include clearly labeled error bars for independent experiments and sample sizes. Unless justified, error bars should not be shown for technical replicates.
- if n<5, the individual data points from each experiment should be plotted. Any statistical test employed should be justified.
- Source Data should be included to report the data underlying figures according to the guidelines set out in the authorship guidelines on Data Presentation.

2. Captions
Each figure caption should contain the following information, for each panel where they are relevant:
- a specification of the experimental system investigated (eg cell line, species name).
- the assay(s) and method(s) used to carry out the reported observations and measurements.
- an explicit mention of the biological and chemical entity(ies) that are being measured.
- an explicit mention of the biological and chemical entity(ies) that are altered/varied/perturbed in a controlled manner.
- the exact sample size (n) for each experimental group/condition, given as a number, not a range;
- a description of the sample collection allowing the reader to understand whether the samples represent technical or biological replicates (including how many animals, litters, cultures, etc.).
- a statement of how many times the experiment shown was independently replicated in the laboratory.
- definitions of statistical methods and measures:
  - common tests, such as t-test (please specify whether paired vs. unpaired), simple χ2 tests, Wilcoxon and Mann-Whitney tests, can be unambiguously identified by name only, but more complex techniques should be described in the methods section;
  - are tests one-sided or two-sided?
  - are there adjustments for multiple comparisons?
  - exact statistical test results, e.g., P values = x but not P values < x;
  - definition of 'center values' as median or average;
  - definition of error bars as s.d. or s.e.m.

Please complete ALL of the questions below.
Select "Not Applicable" only when the requested information is not relevant for your study.

Materials

Newly Created Materials	Information included in the manuscript?	In which section is the information available? (Reagents and Tools Table, Materials and Methods, Figures, Data Availability Section)
New materials and reagents need to be available; do any restrictions apply?	Not Applicable	

Antibodies	Information included in the manuscript?	In which section is the information available? (Reagents and Tools Table, Materials and Methods, Figures, Data Availability Section)
For **antibodies** provide the following information:<br>- Commercial antibodies: RRID (if possible) or supplier name, catalogue number and or/clone number<br>- Non-commercial: RRID or citation	Yes	Materials and Methods

DNA and RNA sequences	Information included in the manuscript?	In which section is the information available? (Reagents and Tools Table, Materials and Methods, Figures, Data Availability Section)
**Short novel DNA or RNA including primers, probes**: provide the sequences.	Yes	Table EV7

Cell materials	Information included in the manuscript?	In which section is the information available? (Reagents and Tools Table, Materials and Methods, Figures, Data Availability Section)
**Cell lines:** Provide species information, strain. Provide accession number in repository **OR** supplier name, catalog number, clone number, and/**OR** RRID.	Yes	Materials and Methods
**Primary cultures:** Provide species, strain, sex of origin, genetic modification status.	Not Applicable	
Report if the cell lines were recently **authenticated** (e.g., by STR profiling) and tested for mycoplasma contamination.	Yes	Materials and Methods

Experimental animals	Information included in the manuscript?	In which section is the information available? (Reagents and Tools Table, Materials and Methods, Figures, Data Availability Section)
**Laboratory animals or Model organisms:** Provide species, strain, sex, age, genetic modification status. Provide accession number in repository **OR** supplier name, catalog number, clone number, **OR** RRID.	Yes	Materials and Methods
**Animal observed in or captured from the field:** Provide species, sex, and age where possible.	Not Applicable	
Please detail **housing and husbandry conditions**.	Yes	Materials and Methods

Plants and microbes	Information included in the manuscript?	In which section is the information available? (Reagents and Tools Table, Materials and Methods, Figures, Data Availability Section)
**Plants:** provide species and strain, ecotype and cultivar where relevant, unique accession number if available, and source (including location for collected wild specimens).	Not Applicable	
**Microbes:** provide species and strain, unique accession number if available, and source.	Not Applicable	

Human research participants	Information included in the manuscript?	In which section is the information available? (Reagents and Tools Table, Materials and Methods, Figures, Data Availability Section)
If collected and within the bounds of privacy constraints report on age, sex and gender or ethnicity for all study participants.	Not Applicable	

Core facilities	Information included in the manuscript?	In which section is the information available? (Reagents and Tools Table, Materials and Methods, Figures, Data Availability Section)
If your work benefited from core facilities, was their service mentioned in the acknowledgments section?	Yes	Acknowledgments

Design

Study protocol	Information included in the manuscript?	In which section is the information available? (Reagents and Tools Table, Materials and Methods, Figures, Data Availability Section)
If study protocol has been **pre-registered, provide DOI in the manuscript**. For clinical trials, provide the trial registration number **OR** cite DOI.	Not Applicable	
Report the **clinical trial registration number** (at ClinicalTrials.gov or equivalent), where applicable.	Not Applicable	

Laboratory protocol	Information included in the manuscript?	In which section is the information available? (Reagents and Tools Table, Materials and Methods, Figures, Data Availability Section)
Provide DOI OR other citation details if **external detailed step-by-step protocols** are available.	Yes	Materials and Methods

Experimental study design and statistics	Information included in the manuscript?	In which section is the information available? (Reagents and Tools Table, Materials and Methods, Figures, Data Availability Section)
Include a statement about **sample size** estimate even if no statistical methods were used.	Yes	Figures
Were any steps taken to minimize the effects of subjective bias when allocating animals/samples to treatment (e.g. **randomization procedure**)? If yes, have they been described?	Not Applicable	
Include a statement about **blinding** even if no blinding was done.	Not Applicable	
Describe **inclusion/exclusion criteria** if samples or animals were excluded from the analysis. Were the criteria pre-established?<br><br>If sample or data points were omitted from analysis, report if this was due to attrition or intentional exclusion and provide justification.	Not Applicable	
For every figure, are **statistical tests** justified as appropriate? Do the data meet the assumptions of the tests (e.g., normal distribution)? Describe any methods used to assess it. Is there an estimate of variation within each group of data? Is the variance similar between the groups that are being statistically compared?	Yes	Figures

Sample definition and in-laboratory replication	Information included in the manuscript?	In which section is the information available? (Reagents and Tools Table, Materials and Methods, Figures, Data Availability Section)
In the figure legends: state number of times the experiment was **replicated** in laboratory.	Not Applicable	Figures
In the figure legends: define whether data describe **technical or biological replicates**.	Yes	Figures

Ethics

Ethics	Information included in the manuscript?	In which section is the information available? (Reagents and Tools Table, Materials and Methods, Figures, Data Availability Section)
Studies involving **human participants**: State details of **authority granting ethics approval** (IRB or equivalent committee(s), provide reference number for approval.	Not Applicable	
Studies involving **human participants**: Include a statement confirming that **informed consent** was obtained from all subjects and that the experiments conformed to the principles set out in the WMA Declaration of Helsinki and the Department of Health and Human Services Belmont Report.	Not Applicable	
Studies involving **human participants:** For publication of **patient photos**, include a statement confirming that consent to publish was obtained.	Not Applicable	
Studies involving experimental **animals**: State details of **authority granting ethics approva**l (IRB or equivalent committee(s), provide reference number for approval. Include a statement of compliance with ethical regulations.	Not Applicable	
Studies involving **specimen and field samples:** State if relevant **permits** obtained, provide details of authority approving study; if none were required, explain why.	Not Applicable	

Dual Use Research of Concern (DURC)	Information included in the manuscript?	In which section is the information available? (Reagents and Tools Table, Materials and Methods, Figures, Data Availability Section)
Could your study fall under dual use research restrictions? Please check biosecurity documents and list of **select agents and toxins** (CDC): https://www.selectagents.gov/sat/list.htm	Not Applicable	
If you used a select agent, is the security level of the lab appropriate and reported in the manuscript?	Not Applicable	
If a study is subject to dual use research of concern regulations, is the name of the **authority granting approval and reference number** for the regulatory approval provided in the manuscript?	Not Applicable	

Reporting

The MDAR framework recommends adoption of discipline-specific guidelines, established and endorsed through community initiatives. Journals have their own policy about requiring specific guidelines and recommendations to complement MDAR.

Adherence to community standards	Information included in the manuscript?	In which section is the information available? (Reagents and Tools Table, Materials and Methods, Figures, Data Availability Section)
State if relevant guidelines or checklists (e.g., **ICMJE, MIBBI, ARRIVE, PRISMA**) have been followed or provided.	Not Applicable	
For **tumor marker prognostic studies,** we recommend that you follow the **REMARK** reporting guidelines (see link list at top right). See author guidelines, under 'Reporting Guidelines'. Please confirm you have followed these guidelines.	Not Applicable	
For **phase II and III randomized controlled trials**, please refer to the **CONSORT** flow diagram (see link list at top right) and submit the CONSORT checklist (see link list at top right) with your submission. See author guidelines, under 'Reporting Guidelines'. Please confirm you have submitted this list.	Not Applicable	

Data Availability

Data availability	Information included in the manuscript?	In which section is the information available? (Reagents and Tools Table, Materials and Methods, Figures, Data Availability Section)
Have **primary datasets** been deposited according to the journal's guidelines (see 'Data Deposition' section) and the respective accession numbers provided in the Data Availability Section?	Yes	Data Availability section
Were **human clinical and genomic datasets** deposited in a public access-controlled repository in accordance to ethical obligations to the patients and to the applicable consent agreement?	Not Applicable	
Are **computational models** that are central and integral to a study available without restrictions in a machine-readable form? Were the relevant accession numbers or links provided?	Yes	Data Availability section
If publicly available data were reused, provide the respective **data citations in the reference list**.	Yes	Main text, References
